# The H2Bub1-deposition complex is required for human and mouse cardiogenesis

**DOI:** 10.1242/dev.201899

**Published:** 2023-12-01

**Authors:** Syndi Barish, Kathryn Berg, Jeffrey Drozd, Isabella Berglund-Brown, Labeeqa Khizir, Lauren K. Wasson, Christine E. Seidman, Jonathan G. Seidman, Sidi Chen, Martina Brueckner

**Affiliations:** ^1^ Department of Genetics, Yale University School of Medicine, 333 Cedar Street, New Haven, CT 06510, USA; ^2^ Department of Pediatrics, Yale University School of Medicine, 333 Cedar Street, New Haven, CT 06510, USA; ^3^ Department of Genetics, Harvard Medical School, Boston, MA 02115, USA; ^4^ Division of Cardiovascular Medicine, Brigham and Women's Hospital, Boston, MA 02115, USA; ^5^ Howard Hughes Medical Institute, Harvard University, Boston, MA 02115, USA

**Keywords:** Cardiac morphogenesis, Cardiomyocyte, Congenital heart disease, Epigenetics

## Abstract

*De novo* variants affecting monoubiquitylation of histone H2B (H2Bub1) are enriched in human congenital heart disease. H2Bub1 is required in stem cell differentiation, cilia function, post-natal cardiomyocyte maturation and transcriptional elongation. However, how H2Bub1 affects cardiogenesis is unknown. We show that the H2Bub1-deposition complex (RNF20-RNF40-UBE2B) is required for mouse cardiogenesis and for differentiation of human iPSCs into cardiomyocytes. Mice with cardiac-specific *Rnf20* deletion are embryonic lethal and have abnormal myocardium. We then analyzed H2Bub1 marks during differentiation of human iPSCs into cardiomyocytes. H2Bub1 is erased from most genes at the transition from cardiac mesoderm to cardiac progenitor cells but is preserved on a subset of long cardiac-specific genes. When H2Bub1 is reduced in iPSC-derived cardiomyocytes, long cardiac-specific genes have fewer full-length transcripts. This correlates with H2Bub1 accumulation near the center of these genes. H2Bub1 accumulation near the center of tissue-specific genes was also observed in embryonic fibroblasts and fetal osteoblasts. In summary, we show that normal H2Bub1 distribution is required for cardiogenesis and cardiomyocyte differentiation, and suggest that H2Bub1 regulates tissue-specific gene expression by increasing the amount of full-length transcripts.

## Teaser

**Summary:** RNF20 and UBE2B monoubiquitylate H2Bub and are associated with human congenital heart disease. Mutations affecting the H2Bub1-deposition complex result in abnormalities in mice and human iPSC-derived cardiomyocytes.

## INTRODUCTION

Congenital heart disease (CHD), a structural abnormality of the heart and/or great vessels, is the most common cause of mortality from congenital malformations. Genetic variants affecting a broad spectrum of chromatin modifier genes contribute to at least 2.3% of cases (Homsy et al., 2015; Jin et al., 2017; Sifrim et al., 2016; Zaidi et al., 2013). These include genes affecting the poorly understood H2Bub1 (monoubiquitylation of histone H2B on K120) mark. Furthermore, there is significant enrichment in H2Bub1-deposition complex members in individuals with CHD compared with controls (Robson et al., 2019). However, how H2Bub1 affects structural cardiogenesis remains enigmatic.

The H2Bub1 machinery was first discovered in yeast; in mammals, deposition of H2Bub1 requires a complex consisting of the E3 ubiquitin ligases RNF20 and RNF40, and the ubiquitin-conjugating enzyme E2 B (UBE2B) in addition to interaction with the WW domain-containing adaptor with coiled-coil (WAC) (Gallego et al., 2020; Kim et al., 2018, 2009, 2005; Robzyk et al., 2000; Wood et al., 2003; Zhang and Yu, 2011). The H2Bub1 mark is located on gene bodies, where it is enriched at the 5′ UTR and gradually decreases towards the 3′ end (Jung et al., 2012). It is postulated to function in both activating and repressing gene expression (Lee et al., 2007; Xie et al., 2017), and the effect of H2Bub1 on transcriptional regulation may be context dependent. H2Bub1 has broad biological functions, including differentiation, tumor suppression and inflammation (Chen et al., 2012a; Fuchs et al., 2012; Karpiuk et al., 2012; Shema et al., 2011, 2008; Tarcic et al., 2016; Wang et al., 2013). In vertebrates, constitutive deletion of *Rnf20* in mouse leads to failure of preimplantation development (Ooga et al., 2015; Xu et al., 2016), conditional deletion in the mouse testes results in male infertility (Xu et al., 2016) and knockdown in *Xenopus* leads to abnormal embryonic left-right axis determination (Robson et al., 2019). The abnormal increase in H2Bub1 levels associated with mutations affecting deubiquitylation results in mid-embryonic lethality in mice (Wang et al., 2021), suggesting that development is sensitive to the H2Bub1 level.

Individuals with CHD show enrichment in *de novo* damaging variants affecting the RNF20 interactome, implicating H2Bub1 in human CHD (Jin et al., 2017; Robson et al., 2019). Knockdown of *Rnf20* and *Rnf40* in *Xenopus* leads to abnormal cilia at the left-right organizer and abnormal heart looping direction. However, individuals with variants affecting H2Bub1 have CHD that is not limited to laterality defects, suggesting that H2Bub1 affects cardiogenesis beyond determination of left-right asymmetry. In addition, postnatal maturation of mouse cardiomyocytes is affected by mosaic deletion of both *Rnf20* and *Rnf40* at postnatal day 0, leading to immature cardiomyocytes at day 28 that are associated with downregulation of adult-biased metabolic genes (VanDusen et al., 2021). However, this observation does not explain the structural cardiac defects resulting from abnormal prenatal development observed in individuals with CHD with variants affecting H2Bub1. This implies an additional essential role for H2Bub1 that is distinct from its function in post-natal cardiomyocyte maturation in the entirely separate process of cardiogenesis, which is under tight transcriptional control and largely completed in mouse by E15 (Brade et al., 2013).

Here, we examined H2Bub1 in cardiogenesis through cardiac-specific deletion of *Rnf20* in mouse embryos, and identify a role for H2Bub1 in cardiomyocyte development *in vivo*. We then examined the dynamic genome-wide distribution of H2Bub1 over time, as human iPSCs differentiate into cardiomyocytes. H2Bub1 was abundant in iPSCs, lost on most genes at the transition from cardiac mesoderm to cardiac progenitor cells, but selectively maintained on a subset of genes significantly enriched in sarcomeric calcium-signaling genes. Finally, we showed that the quantity of full-length transcripts is correlated with local H2Bub1 accumulation near the center of long genes with tissue-specific expression. The combination of mouse embryo and human iPSC-cardiomyocyte data indicates that H2Bub1 is essential for cardiac development through regulation of calcium-signaling and sarcomeric genes during cardiomyocyte differentiation and development.

## RESULTS

### *Rnf20* is expressed during mouse heart development

In embryonic mouse hearts, RNF20 protein demonstrated ubiquitous nuclear expression at E9.5 (Fig. S1A). At E11.5, RNF20 was expressed throughout the epicardium and endocardium, and formed an expression gradient in the myocardium with higher RNF20 at the myocardial surface compared with the lumen. Some cytoplasmic signal was observed at this stage, which could reflect a secondary, non-nuclear role of RNF20, as seen in a different RING finger protein, MURF-1 (Fig. S1B) (McElhinny et al., 2002). We next determined whether changes in levels of the H2Bub1-deposition complex components (RNF20-RNF40-UBE2B) are correlated with H2Bub1 levels during mouse heart development and showed dynamic levels of H2Bub1-deposition complex members between E9.5 and P0, along with increasing H2Bub1/H2B in mouse embryo hearts during cardiogenesis from E9.5 to E16.5 (Fig. S1C).

### Normal H2Bub1-deposition complex levels are required for mouse heart development

**Fig. 1.**
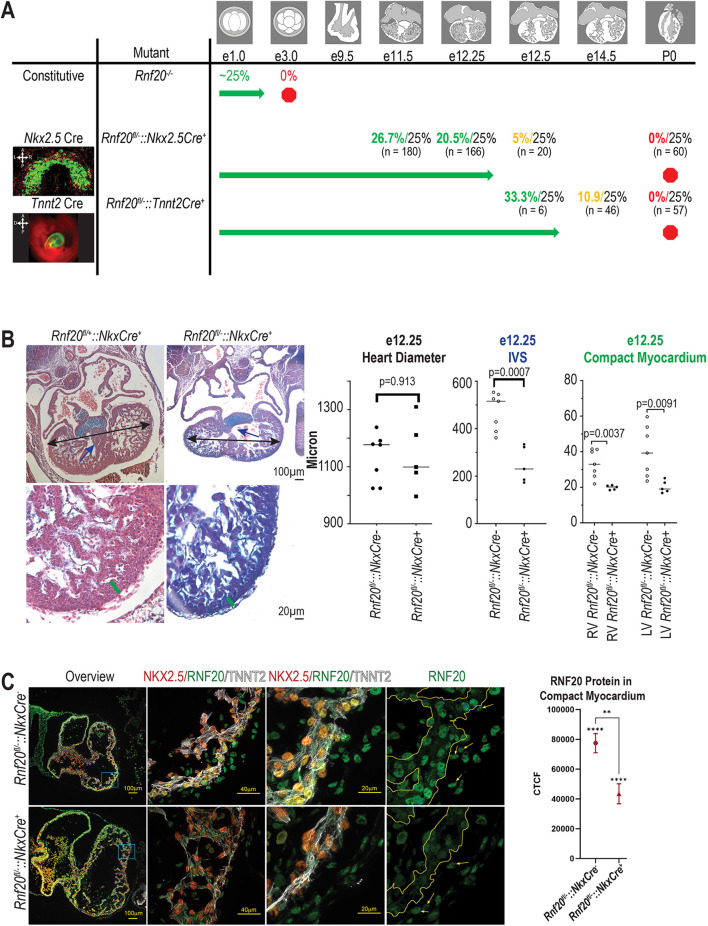
***Rnf20* is required for heart development.** (A) *Rnf20* mutant mouse survival chart for constitutive nulls, *Rnf20*^fl/−^::*Nkx2.5*Cre^+^ and *Rnf20*^fl/−^::*Tnnt2*Cre^+^. Colored percentages indicate the observed percentage of null or conditionally null mice (green indicates mendelian ratios, yellow indicates deviation from mendelian ratios and red indicates no mice). Percentages in black indicate the predicted percentages. The sample size is listed below each percentage. See Figs S2-S4 for more details on these crosses. Images are of the cross between a *Nkx2.5-*cre positive [E8.5 (cardiac crescent), signal in cardiac crescent] or a *Tnnt2*-cre positive [E9.5 (heart tube), signal in cardiomyocytes] with a ROSA^mt/mg^ mouse to illustrate the distribution of Cre-positive cells. Drawings indicate the expected morphology at each stage of mouse heart development. A, anterior; P, posterior; L, left; R, right. (B) Representative Hematoxylin and Eosin stained E12.25 wild-type (*Rnf20*^fl/+^::*Nkx2.5*Cre^+^) and mutant (*Rnf20*^fl/−^::*Nkx2.5*Cre^+^) mice. Quantification of heart diameter, interventricular septum (IVS) length, thickness of right ventricle (RV) compact myocardium and thickness of left ventricle (LV) compact myocardium are displayed as individual data points with a line representing the median (*n*=7 wild-type and *n*=5 mutant hearts). Unpaired two-tailed, heteroscedastic *t*-test. (C) Immunofluorescent staining for NKX2.5 (red), RNF20 (green) and TNNT2 (myocardium, white) in E10.5 wild-type (*Rnf20*^fl/−^::*Nkx2.5*Cre^−^) and mutant (*Rnf20*^fl/−^::*Nkx2.5*Cre^+^) mouse hearts. Embryos are reimaged for Fig. 2C. Corrected total cell fluorescence (CTCF) for RNF20 in the compact myocardium is given. **P≤0.01, ****P≤0.0001 (two-tailed one-sample *t*-test between replicates and two-tailed unpaired *t*-test with Welch's correction between groups).

**Fig. 2.**
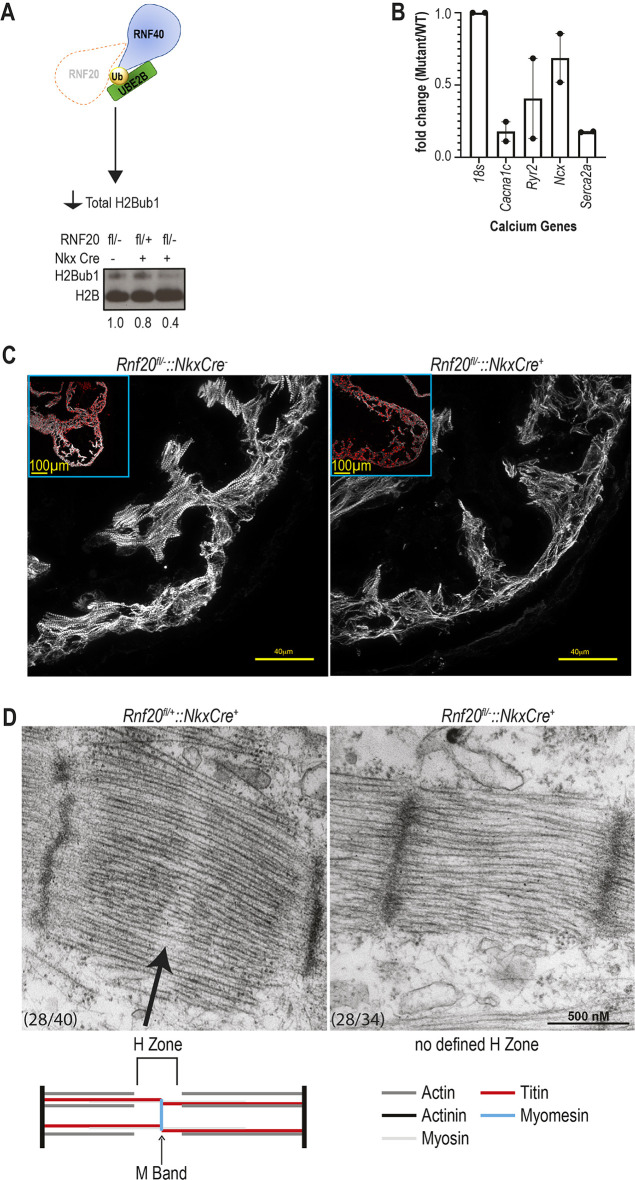
**Normal H2Bub1-depostion complex levels are required for normal cardiomyocytes *in vivo*.** (A) H2Bub1-deposition complex schematic illustrating the *Rnf20^fl/−^* mouse mutant, which leads to decreased total H2Bub1 levels. Below is a western blot for H2Bub1 in wild-type (*Rnf20*^fl/−^::*Nkx2.5*Cre^−^ and *Rnf20*^fl/+^::*Nkx2.5*Cre^+^) and mutant (*Rnf20*^fl/−^::*Nkx2.5*Cre^+^) mouse hearts at E12.25. The loading control is H2B. Numbers indicate quantification (imageJ) of H2Bub1 normalized to H2B. (B) Quantitative RT-PCR of calcium-signaling genes in E11.5 wild-type (*Rnf20*^fl/+^::*Nkx2.5*Cre^+^) and mutant (*Rnf20*^fl/−^::*Nkx2.5*Cre^+^) mouse hearts for *Cacna1c*, *Ryr2*, *Ncx* and *Serca2a*. Levels of expression are normalized to 18s (*Rn18s*) rRNA. Individual data points are shown as black dots. Data are mean±s.e.m. (*n*=2). (C) The embryos depicted in Fig. 1C are reimaged focusing on immunofluorescent staining for TNNT2 (myocardium, white) in E10.5 wild-type (*Rnf20*^fl/−^::*Nkx2.5*Cre^−^ and mutant (*Rnf20*^fl/−^::*Nkx2.5*Cre^+^) mouse hearts. (D) Transmission electron microscopy of E12.25 wild-type (*Rnf20*^fl/+^::*Nkx2.5*Cre^+^, *n*=2) and mutant (*Rnf20*^fl/−^::*Nkx2.5*Cre^+^, *n*=2) mouse heart sarcomeres. Arrow indicates the H zone.

To examine the function of H2Bub1 in cardiogenesis, we deleted *Rnf20*, a component of the H2Bub1-deposition complex, *in vivo* in mice. We chose an E3 ligase, RNF20, because the associated ubiquitylases, UBE2A and UBE2B, are 95% identical at the protein level, and could compensate for each other (Sarcevic et al., 2002). Because constitutive deletion of *Rnf20* in mouse is pre-implantation lethal (Fig. 1A, Fig. S2) (Ooga et al., 2015; Valenzuela et al., 2003; Xu et al., 2016), we generated mice with cardiac-specific deletion of *Rnf20* using a conditional *Rnf20*^fl^ allele containing loxP sites flanking exons 2-4 of the *Rnf20* gene (Xu et al., 2016) and two independent cardiac Cre drivers: *Nkx2.5*Cre (1st heart field, predominantly cardiac myocytes) (Moses et al., 2001) and *Tnnt2*Cre (exclusively cardiac myocytes) (Jiao et al., 2003) (Fig. S3A). ROSA^mt/mg^ matings were used to evaluate the timing and specificity of Cre expression (Fig. 1A). *Rnf20*^fl/+^::*Nkx2.5*Cre^+^ mice are found at Mendelian ratios through birth and appear phenotypically normal, indicating *Nkx2.5* haploinsufficiency resulting from the *Nkx2.5*Cre allele does not cause a phenotype (Fig. S3B). *Rnf20*^fl/−^::*Nkx2.5*Cre^+^ embryos are found at Mendelian ratios until E12.25, but none were recovered after E12.5 (Fig. 1A, Fig. S3B). At E11.5, the embryos appear externally normal and the heart appears normal by Hematoxylin and Eosin staining, including 100% normal heart looping direction (Fig. S3C). By E12.25, the compact myocardium is significantly thinned in *Rnf20*^fl/−^::*Nkx2.5*Cre^+^ embryos, and the ventricular septum is deficient (left ventricle compact myocardium thickness *P*=0.009, right ventricle compact myocardium thickness *P*=0.004, septum length *P*=0.0007) (Fig. 1B). We verified the *Nkx2.5*-specific deletion of *Rnf20* at E10.5; nuclear RNF20 protein is broadly expressed in myocardial, endocardial and epicardial cells in the *Rnf20*^fl/−^::*Nkx2.5*Cre^−^ embryos, but is deleted in myocardial cells and retained only in the NKX2.5-negative epicardial and endocardial cells in *Rnf20*^fl/−^::*Nkx2.5*Cre^+^ embryos (Fig. 1C, Fig. S3D). As RNF20 is responsible for H2Bub1 deposition, and we show that *Rnf20* deletion results in a reduction of H2Bub1 in the mouse heart (Fig. 2A), we suggest that the observed phenotype may be the result of altered H2Bub1, although this does not exclude the possibility that a non-enzymatic function of RNF20 contributes to some of the final cardiac phenotype.

As Nkx2.5-expressing cells primarily contribute to myocardial cells, but also to some endocardial cells, we tested whether the phenotype specifically results from RNF20 function in cardiomyocytes by investigating the effect of *Rnf20* deletion using *Tnnt2Cre* (Jiao et al., 2003), which is expressed exclusively in the cardiomyocytes. We verified the *Tnnt2*-specific deletion of *Rnf20* in myocardium at E11.5 (Fig. S4A). No live-born *Rnf20*^fl/−^::*Tnnt2*Cre^+^ embryos and only a few *Rnf20*^fl/−^::*Tnnt2*Cre^+^ necrotic embryos were recovered at E13.5-E15.5. Both *Rnf20*^fl/+^::*Tnnt2*Cre^−^ and *Rnf20*^fl/−^::*Tnnt2*Cre^+^ embryos were significantly underrepresented at the P0 stage (χ^2^test, *P*<0.00001) (Fig. S4B), suggesting linkage between the *Tnnt2* transgene insertion site and the *Rnf20* locus (Fig. 1A, Fig. S4B). The longest surviving *Rnf20*^fl/−^::*Tnnt2*Cre^+^ embryo had edema and a pericardial effusion at E14.0 (Fig. S4C), and the myocardium was abnormal at E10.5 (Fig. S4D). Together, these data point to a primary function for RNF20 during cardiogenesis in cardiomyocyte development (Fig. 1A, Figs S3 and S4) that results in defective compact myocardium and deficient ventricular septum, which is consistent with some of the structural CHD observed in humans with variants affecting H2Bub1-deposition complex components.

### Reduced H2Bub1-deposition complex levels result in abnormal sarcomeres in mice

The myocardial phenotype observed in mouse embryos led us to examine the effect of loss of RNF20 on cardiomyocytes by investigating sarcomere structure by microscopy. Immunofluorescent staining of heart sections from E10.5 in *Rnf20^fl/−^::Nkx2.5*Cre^+^ and *Rnf20^fl/−^::Tnnt2*Cre^+^ embryos before any other visible cardiac phenotype showed disorganized myocardium and altered distribution of the sarcomeric protein TNNT2 (Fig. 2C, Fig. S4D). We then analyzed embryonic calcium gene expression [*Cacna1c*, *Ryr2*, *Ncx* (*Slc8a1*) and *Serca2a* (*Atp2a2*)] at E11.5 in *Rnf20^fl/+^::Nkx2.5*Cre^+^ and *Rnf20^fl/−^::Nkx2.5*Cre^+^ embryonic hearts, before any visible cardiac defects. We observed that *Rnf20^fl/−^::Nkx2.5*Cre^+^ hearts had lower *Cacna1c* and *Serca2a* expression than *Rnf20^fl/+^::Nkx2.5*Cre^+^ siblings (Fig. 2B). Sarcomere structure was examined by transmission electron microscopy (TEM) of E12.25 *Rnf20^fl/−^::Nkx2.5*Cre^+^ mouse embryos, which revealed that sarcomeres are missing the H zone compared with E12.25 *Rnf20^fl+^::Nkx2.5*Cre^+^ littermates (Fig. 2D). The normal H zone is the region of the sarcomere devoid of actin filaments. The center of the H zone is the M band, which, at E12.25, consists mostly of myomesin, is not visible by TEM at E12.25 due to an elastic domain in the middle of the embryonic splice variant and functions to anchor the actin filaments to titin (Lange et al., 2020). An abnormal M band will lead to abnormal sarcomere organization, as observed in *Rnf20^fl/−^::Nkx2.5*Cre^+^ mice. Thus, the H2Bub1-deposition complex is necessary for embryonic cardiomyocyte development by regulating sarcomeric structure, as well as modulating the expression of a subset of calcium-signaling genes in embryonic mouse hearts.

### H2Bub1 is dynamically distributed during human cardiomyocyte differentiation

**Fig. 3.**
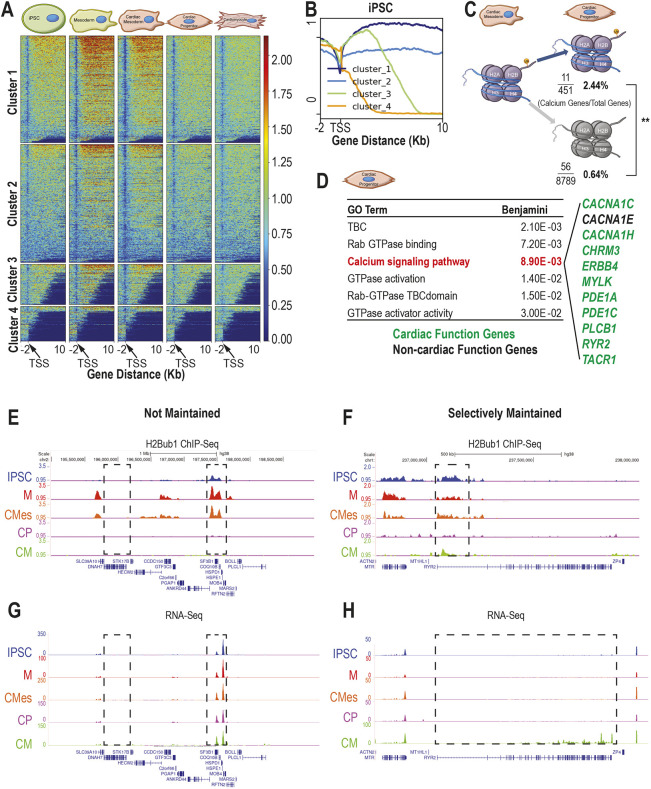
**H2Bub1 in iPSC-derived cardiomyocyte development shows selective maintenance of calcium-signaling genes.** (A) H2Bub1 surrounding the transcriptional start site (TSS) (−2 kb, +10 kb) genome-wide in five stages of CM differentiation [iPSC, mesoderm (M), cardiac mesoderm (CMes), cardiac progenitor (CP) and cardiomyocyte (CM) stages]. Genes are grouped by H2Bub1 occupancy into four clusters. The profile representing the average of three replicates, depicted using fold enrichment against random distribution values, across this region for each cluster in each stage is shown as a heatmap. (B) The cluster breakdown for iPSCs in A. Cluster 1 indicates high H2Bub1 levels, cluster 2 indicates low H2Bub1 levels, cluster 3 indicates moderate H2Bub1 levels and cluster 4 indicates no H2Bub1. (C) Proportion of genes that maintain H2Bub1 from CMes to CP stages that are calcium-signaling genes and the proportion of genes that have decreased H2Bub1 that are calcium-signaling genes. One-tailed, Z-score (**P<0.01). (D) The significant gene ontology terms from the genes near regions that maintain H2Bub1 between cardiac mesoderm (CMes) and cardiac progenitor (CP) stages. Genes associated with calcium-signaling pathways are listed and are in green if they are associated with cardiac function from patient variants and/or mouse models; they are in black if they are not. (E,F) Representative H2Bub1 occupancy, depicted using fold enrichment against random distribution across non-selectively maintained genes (E) and a selectively maintained gene (F) at five stages in cardiomyocyte (CM) differentiation [iPSC (blue), mesoderm (M) (red), CMes (orange), CP (purple) and CM (green) stages]. Gene structure is indicated below the H2Bub1 traces for each gene. The first box (E) highlights a gene that has no H2Bub1 signal at any stage. The second box (E) highlights a gene that has dynamic H2Bub1 signal across the stages. The selectively maintained region is indicated in the box (F). (G,H) Representative RNA-seq traces depicted in TPM across non-selectively maintained genes (G) and a selectively maintained gene (H), corresponding to the genes shown in E and F. Coloring is the same as in E and F, and the same regions are boxed. Gene structure is indicated below the H2Bub1 traces for each gene.

As normal levels of a H2Bub1-deposition complex component, RNF20, are required for cardiomyocyte development *in vivo* in mouse, we next investigated the H2Bub1 profile during cardiomyocyte differentiation using *in vitro* human iPSC-derived cardiomyocytes (CMs) instead of a heterogeneous organ (see Materials and Methods) (Lian et al., 2013). The genome-wide H2Bub1 profile and corresponding transcriptional changes during CM differentiation were analyzed by ChIP-seq of H2Bub1 and bulk RNA-seq at five stages of CM differentiation: iPSCs, mesoderm (M), cardiac mesoderm (CMes), cardiac progenitor (CP) and CM (Fig. S5, Tables S5 and S6). The general H2Bub1 profile is as previously reported, with very low occupancy at the transcription start site (TSS) and coverage over the entire gene body with gradual diminution from 5′ to 3′ (Jung et al., 2012) (Fig. 3B). Furthermore, genome-wide H2Bub1 occupancy increases between iPSC and M stages near the TSS, but remains constant between M and CMes stages. It then decreases between CMes and CP stages, and again remains constant between CP and CM stages (Fig. 3A, Fig. S6A, Table S5). Minimal H2Bub1 at the terminally differentiated CM stage agrees with previous work showing that the terminally differentiated muscle cells also had minimal H2Bub1 (Vethantham et al., 2012).

After qualitatively exploring H2Bub1 marks in the genic regions, we next checked for H2Bub1 marks on heterochromatic regions. Our first hint that H2Bub1 may be present in the heterochromatic regions was the lack of congruence between total H2Bub1 (measuring both genic and heterochromatic H2Bub1) and the overall pattern of H2Bub1 on the genic regions based on the ChIP-seq data (Fig. 3A, Fig. S6B). Total H2Bub1 during the transition from iPSC to M stages increases and stays stable from M to CMes stages (Fig. S6B), consistent with the genic ChIP-seq data. In contrast, total H2Bub1 increases from CMes to CP stages, whereas ChIP-seq shows a decrease in H2Bub1 around gene bodies (Fig. 3A, Fig. S6B). One explanation is that H2Bub1 is decreasing in occupancy around the gene body but increasing in occupancy in heterochromatic regions. In agreement with this hypothesis, we found significantly more H2Bub1 in the previously defined B compartment (heterochromatic compartment) than expected by chance at the CM stage (*P*-values<1×10^−5^) (see Materials and Methods) (Bertero et al., 2019) (Fig. S6C). Furthermore, between the iPSC and CM stages, the overlap between H2Bub1 and previously published active marks (H3K4me3) remains constant, whereas the overlap between H2Bub1 and heterochromatin marks (H3K27me3 and H3K9me3) significantly increases (H3K27me3 *P*=0.04, H3K9me3 *P*=0.00006) (see Materials and Methods) (ENCODE Project Consortium, 2012; Luo et al., 2020) (Fig. S6D,E, example tracks in Fig. S7). The constant overlap in H2Bub1 and H3K4me3 between the iPSC and CM stages (Fig. S6D) appears to be in contradiction to the dynamic H2Bub1 levels on the gene bodies (Fig. 3A). However, the genes marked by both H2Bub1 and H3K4me3 have H2Bub1 marks at distal intergenic regions more often than at promoter regions compared with the genes marked by both H2Bub1 and H3K4me3 at the iPSC stage (Fig. S6F,G). We next determined that H2Bub1 marks on genes in the A compartment tend to be on the gene (promoter and intronic regions), whereas marks on genes in the B compartment tend to be at distal intergenic locations (Fig. S6H). Furthermore, as we would predict based on expression of genes in the different compartments, genes with H2Bub1 marks in the B compartment are significantly less expressed than genes with H2Bub1 in the A compartment (Fig. S6I). Together, these data indicate that H2Bub1 increases in heterochromatic regions from CMes to CM stages, which accounts for the discrepancy between the observed increase in total H2Bub1 from CMes to CM stages, and the observed decrease in genic H2Bub1.

### H2Bub1 is selectively maintained on calcium-signaling genes *in vitro*

To determine what genes change in H2Bub1 occupancy over the progression from iPSC to CM stages, we performed a differential binding analysis on the ChIP-seq data (see Materials and Methods). We found 316 regions (corresponding to 256 ENSEMBL genes) that have increased H2Bub1 between iPSC and M stages, compared with 18 regions (corresponding to 11 ENSEMBL genes) with decreased H2Bub1. Between M and CMes stages, there are two regions (corresponding to 2 ENSEMBL genes) with decreased H2Bub1. The largest change in H2Bub1 happens upon transition from CMes to CP stages, with a decrease in 25,748 regions (corresponding to 8414 ENSEMBL genes), whereas no gene-specific changes in H2Bub1 between CP and CM were observed. The genes with changing H2Bub1 occupancy during iPSC-CM differentiation cover a wide-range of general cell functions (Figs S8 and S9, Table S5). Next, we performed differential expression analysis on the bulk RNA-seq data and found stage-specific gene expression at each transition (Figs S8 and S9, Table S6). Finally, we compared the differentially occupied genes with the differentially expressed genes at each stage. When investigating downregulated and decreased occupancy genes, there is one gene in common at the iPSC to M transition, 0 genes in common at the M to CMes transition and there are 711 genes in common at the CMes to CP transition. The CP to CM transition has no genes with decreased H2Bub1 (Fig. S8). Twenty-one genes are shared between upregulated and increased occupancy genes at the iPSC to M transition (Fig. S9). The overlapping genes also have a range of general cell functions (Figs S8 and S9).

The largest decrease in H2Bub1 occupancy occurs during the transition from CMes to CP, so we analyzed the 451 genes that maintain the mark at the CP stage (Fig. S10). Genes that maintain H2Bub1 at the CP stage are significantly enriched for calcium-signaling genes, and 10 of the 11 calcium-signaling genes that maintain H2Bub1 are associated with cardiac function through patient variants and/or mouse models (Fig. 3D) (Boczek et al., 2015; Kepenek et al., 2020; Knight et al., 2016; Mazzarotto et al., 2021; Tang et al., 2012; Vasilescu et al., 2018; Vasti and Hertig, 2014; Wang et al., 2016, 2017; Xu et al., 2018). These include *CACNA1C* and *RYR2*, which have been linked to left ventricular non-compaction (LVNC) (Mazzarotto et al., 2021; Vasilescu et al., 2018). Furthermore, a significantly higher proportion of calcium-signaling genes was identified within the genes that have maintained H2Bub1 compared with genes with downregulated H2Bub1 (Z score=4.3996, *P*=1.1×10^−5^) (Fig. 3C). Calcium-signaling genes remain constant in H2Bub1 occupancy over time, unlike housekeeping genes, which are either not occupied by H2Bub1 at all or have increased H2Bub1 marks at the M stage compared with the iPSC stage, and fewer marks at the CP stage compared with the CMes stage. We show some representative examples of housekeeping genes that are not expressed during CM differentiation and housekeeping genes that are expressed at all observed stages of CM differentiation (Fig. 3E,G). In contrast, the sarcomeric calcium gene *RYR2* maintains H2Bub1 from iPSC through CM stage and becomes expressed between the CP and CM stages (Fig. 3F,H). Together, these data indicate that H2Bub1 is selectively maintained on tissue-specific genes at the CP stage.

### Reduced H2Bub1-deposition complex levels result in abnormal sarcomere and calcium-signaling gene expression *in vitro*

**Fig. 4.**
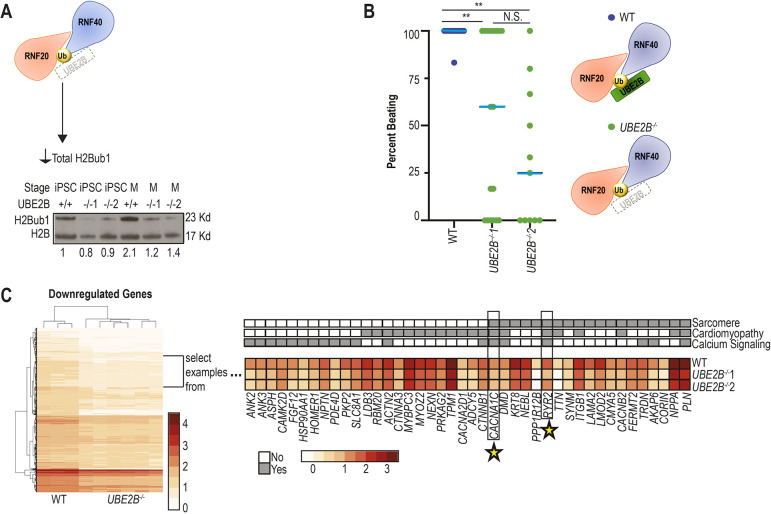
**Normal H2Bub1-deposition complex levels are required for normal cardiomyocyte differentiation *in vitro*.** (A) H2Bub1-deposition complex schematic illustrating the *UBE2B^−/−^* iPSC mutant, which leads to decreased total H2Bub1 levels. Below is a western blot for H2Bub1 in wild-type and mutant cells at the iPSC and mesoderm (M) stages. The loading control is H2B. Numbers indicate average quantification (imageJ) of H2Bub1 normalized to H2B over three replicates. (B) The percentage of each six-well plate (differentiation started on the same day and same strain) of iPSC-derived cardiomyocytes that beats by day 20 is indicated by colored dots [wild type (*n*=12) is blue and *UBE2B^−/−^* (mutant 1, *n*=18; mutant 2, *n*=11) is green]. Data are shown as individual data points with a blue line representing the median. Unpaired two-tailed, heteroscedastic *t*-test (**P<0.01; N.S., not significant). (C) A heatmap showing all the downregulated genes between wild type (three replicates) and *UBE2B^−/−^* (three replicates of both independent lines) at the cardiomyocyte stage from RNA-seq. We focus on a selection of these genes and show gene ontology analysis. Many of these genes are related to sarcomere, cardiomyopathy and/or calcium signaling. The expression levels of the most significant genes in each category are displayed. Stars indicate genes shared with maintained H2Bub1 marks upon transition to CPs shown in Fig. 3D. Gene ontology (upper panel) is in grayscale; mRNA expression data (lower panel) is shown as a heat map.

To determine how defective H2Bub1 impacts iPSC-CM differentiation, we created iPSCs with mutations in the H2Bub1 deposition complex. In an attempt to recapitulate the *Rnf20* deletion mouse model, we initially created *RNF20^+/−^* iPSCs. However, *RNF20^+/−^* iPSCs had variable total H2Bub1 levels, retained Brachyury expression, failed to express NKX2.5 and rarely formed beating cardiomyocytes, prohibiting analysis of CM differentiation (Figs S11 and S12, Movies 1-4). We therefore made mutations in *UBE2B*, which we predicted would be better tolerated due to compensation by the homologous UBE2A (Sarcevic et al., 2002). Two loss-of-function *UBE2B^−/−^* iPSC lines (*UBE2B^−/−^*1 and *UBE2B^−/−^2*) were created in two independent experiments; both contained gene-inactivating mutations. Given the similarity between UBE2B and UBE2A, these knockouts were validated by sequencing the cDNA (Fig. S13A, see Materials and Methods) (Sarcevic et al., 2002). The *UBE2B^−/−^* cell lines had decreased H2Bub1 (Fig. 4A), as observed in the *Rnf20* deletion mice, supporting their use to evaluate the effect of decreased H2Bub1 on iPSC to CM differentiation.

At the iPSC stage, both wild-type and *UBE2B^−/−^* cells express OCT4, indicating that a decrease in *UBE2B* does not affect pluripotency (Fig. S14A). At the mesoderm stage, both wild-type and *UBE2B^−/−^* cells express brachyury, but some *UBE2B^−/−^* cells retain OCT4 expression, suggesting the *UBE2B^−/−^* cells have variable ability to exit pluripotency (Fig. S14B). This variability is supported by the observation that only ∼33% of *UBE2B^−/−^* cells (*UBE2B^−/−^1*, 25/72; *UBE2B^−/−^2*, 24/69) beat at the CM stage (Fig. 4B, Movies 1, 5-8). Given the observed heterogeneity, we evaluated both beating (lactate selection) and non-beating (no lactate selection) cells at a time corresponding to the wild-type CM stage (see Materials and Methods). At the CM stage, both wild-type and *UBE2B^−/−^* cells lost OCT4 and express ISL1, whereas most *UBE2B^−/−^* cells retained brachyury. However, a substantial number of beating *UBE2B^−/−^* cells express NKX2.5 (*UBE2B^−/−^1*, 73.2%; *UBE2B^−/−^2*, 40.0%) and TNNT2 (*UBE2B^−/−^1*, 47.6%; *UBE2B^−/−^2*, 32.6%), compared with non-beating *UBE2B^−/−^* cells (NKX2.5: *UBE2B^−/−^1*, 1.7%; *UBE2B^−/−^2*: 3.0%; TNNT2: *UBE2B^−/−^1*, 5.6%; *UBE2B^−/−^2*, 0%) (Fig. S14C). These data indicate that *UBE2B^−/−^* iPSCs can differentiate into beating CMs but do so at a reduced efficiency compared with wild-type iPSCs, and demonstrate a requirement for UBE2B in normal CM differentiation.

To evaluate gene expression in iPSC-CMs with reduced H2Bub1, we performed RNA-seq on beating *UBE2B^−/−^* CMs and compared them with time-matched wild-type iPSC-derived CMs (Fig. S13B, Table S8). Differential expression analysis identified 1393 downregulated and 1555 upregulated transcripts that are shared between both independent *UBE2B^−/−^* cell lines compared with wild type (see Materials and Methods, Table S8). GO enrichment analysis demonstrated that about half of the significant GO terms (37/76, representing 80 of the downregulated genes) are related to calcium-signaling genes, sarcomere genes and/or cardiomyopathy genes. This correlates with our finding that the *Rnf20^fl/−^::Nkx2.5*Cre^+^ and *Rnf20^fl/−^::Tnnt2*Cre^+^ mice, at a comparable stage of cardiomyocyte development, also have decreased calcium-signaling gene expression and/or altered sarcomeric structure (Fig. 2, Fig. S4D). In addition, *UBE2B^−/−^* CMs have reduced expression of M-band components (myomesin and titin), proteins required for splicing M-band proteins (RBM20 and RBM24) and a protein responsible for creating the H-zone (MURF1), linking the *in vitro* findings with the abnormal H-zone observed by TEM *in vivo* in mouse embryos with decreased H2Bub1 (Fig. 2D) (Chen et al., 2012b; Higashikuse et al., 2019; McElhinny et al., 2002; Schneider et al., 2020; Yang et al., 2014). Two further genes with decreased expression, *CACNA1C* and *RYR2*, are also among the genes with selectively maintained H2Bub1 upon wild-type transition from CMes to CP stages (Figs 3D and 4C). The tissue-specific genes that are downregulated when H2Bub1 is decreased in embryonic cardiomyocytes are distinct from the metabolic genes identified when *Rnf20* and *Rnf40* were downregulated in postnatal cardiomyocytes (VanDusen et al., 2021), indicating that the H2Bub1-deposition complex has two different functions at these two distinct phases of heart development. We conclude that the H2Bub1-deposition complex coordinately regulates sarcomeric and calcium-signaling genes *in vitro* and *in vivo* to program normal embryonic cardiomyocyte development.

### Reduced H2Bub1-deposition complex levels result in reduced levels of full-length transcripts of tissue-specific genes

**Fig. 5.**
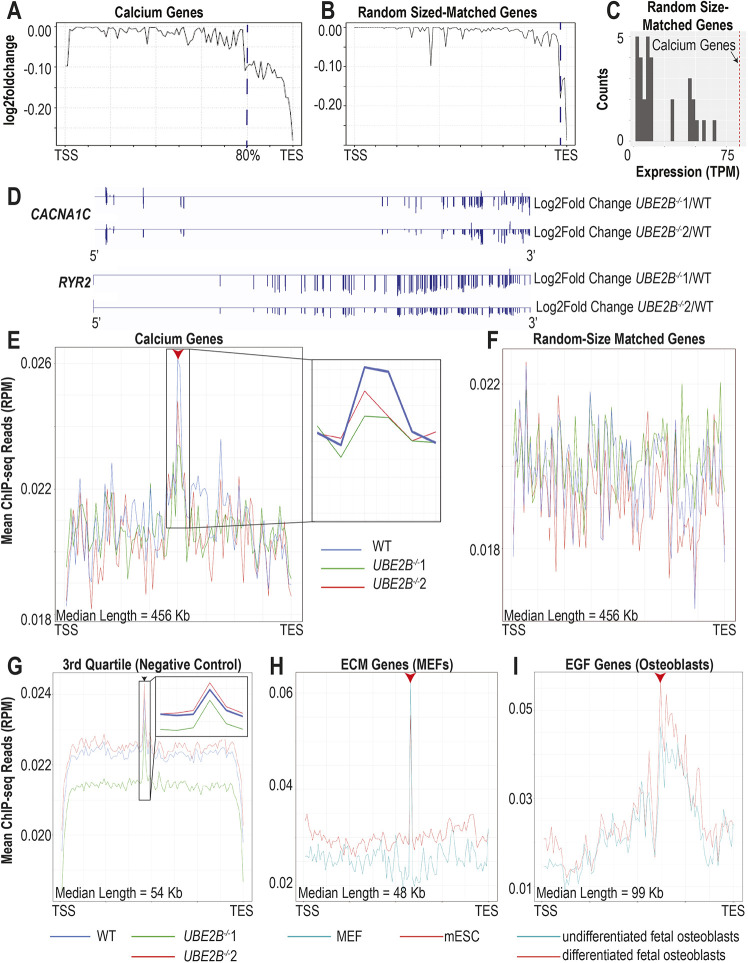
**Abundant full-length transcripts of tissue-specific genes require normal H2Bub1-deposition complex levels.** (A,B) Log2 of fold change in transcript abundance between wild-type and *UBE2B^−/−^* mutants is shown at each position. The genes shown in A are the calcium-signaling genes (*n*=28 genes) that are differentially expressed between wild type and *UBE2B^−/−^* mutants (*n*=3, for each of the two cell lines). The genes shown in B are ‘randomly’ selected quantity- and size-matched to the calcium-signaling gene set (*n*=3, for each of the two cell lines). Thirty ‘random’ plots were created from the calcium-signaling gene set: 10 from genes that are upregulated between wild-type and both *UBE2B^−/−^* cell lines; 10 that are non-regulated between wild-type and both *UBE2B^−/−^* cell lines; and 10 that are downregulated between wild-type and both *UBE2B^−/−^* cell lines. (C) A histogram showing the average expression in TPM of each of the ‘random’ quantity- and size-matched gene set described in B. The vertical dashed red line shows the average expression in TPM of the calcium-signaling genes. (D) Representative log2 of fold change gene traces for calcium-signaling genes (*CACNA1C* and *RYR2*) that have H2Bub1 accumulation near the center of the gene and decreased amounts of full-length transcripts. 5′ on the left of the diagram. (E-G) Metagene plots for H2Bub1 levels in wild-type (blue) and *UBE2B^−/−^* mutants (green and red) (*n*=3). The arrowheads in E and G indicate the accumulation near the center of the gene. The red arrowhead indicates UBE2B-dependent accumulation; the black arrowhead indicates UBE2B-independent accumulation. The genes shown in E are the calcium-signaling genes described in A; the genes shown in F are an example of a ‘random’ quantity- and size-matched gene set described in B; all of the 3rd quartile genes (greater than 33.940 kb and less than 93.323 kb) are shown in G. To the right of E and the inset in G are zoom-ins of the accumulation region. (H) Metagene plot for H2Bub1 levels in MEFs (teal) and mESCs (red) across the ECM genes that are differentially expressed between MEFs and mESCs. The red arrowhead indicates UBE2B-dependent accumulation. (I) Metagene plot for H2Bub1 levels in undifferentiated hFOBs (teal) and differentiated hFOBs (red) across the EGF-related genes that are differentially expressed between undifferentiated hFOBs and differentiated hFOBs. The red arrowhead indicates the accumulation near the center of the gene.

Calcium-signaling and sarcomeric genes, which are significantly longer than expected by chance (calcium-signaling genes are 415 kb longer, *P*<0.0001; sarcomere genes are 125 kb longer, *P*<0.0001), are downregulated in iPSC-derived cardiomyocytes with decreased H2Bub1 (calcium-signaling genes, *n*=28; sarcomere genes, *n*=70) (Fig. S15A,B). In addition, the median length of all downregulated transcripts in *UBE2B^−/−^* iPSC-derived cardiomyocytes (79 kb) is significantly greater than expected by random chance (46 kb longer, *P*<0.0001). Given that the H2Bub1-deposition complex is known to be involved in transcriptional elongation, we hypothesized that H2Bub1 may be modulating the amount of full-length transcripts of long genes. To test this hypothesis, we looked for abbreviated transcripts by evaluating whether there was disproportionate loss of the 3′ end of mRNA in *UBE2B*^−/−^ CMs compared with wild type. We found that mRNA transcript abundance across calcium-signaling and sarcomere genes is lower in the last 20% of the 3′ end of the gene in the *UBE2B^−/−^* cells compared with wild type. Thus, there are fewer full-length transcripts in the mutants (Fig. 5A, Fig. S15C). Example transcript traces for long calcium-signaling genes with reduced amounts of full-length transcripts (*CACNA1C* and *RYR2*) and example transcript traces for short genes with wild-type levels of full-length transcripts (*TMEM117*, *CCDC178* and *ERC1*) are shown (Fig. 5D, Fig. S15G,H). To validate that this conclusion is not a technical artifact, we repeated the same analysis on 60 ‘random’ sets of quantity- and sized-matched gene sets, which have only decreased abundance in the last 5-10% of the 3′ end. We provide one representative graph for each (Fig. 5B, Fig. S15D). It is important to note that these random genes have significantly lower expression than the calcium-signaling and sarcomere genes (Fig. 5C, Fig. S15E). Thus, these data suggest that H2Bub1 is specifically regulating the amount of full-length transcripts of tissue-specific genes in cardiomyocytes.

We next analyzed the H2Bub1 profile across the genes with decreased levels of full-length transcripts in *UBE2B^−/−^* cells. Whereas *UBE2B* loss leads to decreased total H2Bub1, ChIP-seq of CMs comparing H2Bub1 between wild-type and *UBE2B^−/−^* cells demonstrated that gene-specific H2Bub1 is decreased only in eight Ensembl genes (Fig. 4A, Fig. S13C, Table S7). To identify more-subtle differences in the distribution of H2Bub1 marks between *UBE2B^−/−^* and wild-type CMs, we created metagenes corresponding to the downregulated calcium-signaling and sarcomere genes (Table S7). This demonstrated H2Bub1 accumulation near the center of the metagenes in wild-type cells, which was either reduced or completely absent in *UBE2B*^−/−^ cells (Fig. 5E, Fig. S16A). To validate that this accumulation is not a technical artifact, we repeated the analysis on our ‘random’ gene sets and did not find accumulation at the center of the metagenes (examples are shown in Fig. 5F, Fig. S16B). To determine whether H2Bub1 accumulation near the center of genes is gene-length dependent, we generated metagenes based on gene length and found H2Bub1 accumulation near the center of genes in all genes in the 2nd and 3rd quartile for length (Fig. S16D,E). Genes in the 1st quartile (short genes) follow the previously published pattern of H2Bub1 occupancy and genes in the 4th quartile (very long genes) have accumulation closer to the 3′ end, which interestingly does appear to be UBE2B dependent (Fig. S16C,F). However, accumulation near the center of the gene is not UBE2B dependent except in tissue-specific long genes (Fig. 5E,G, Fig. S16A). In addition to the statistical average presented by the metagenes, representative H2Bub1 traces for single tissue-specific and non-tissue-specific genes are shown (Fig. S17). It is important to note that the 3rd quartile genes (the quartile containing the calcium-signaling and sarcomere genes) also have significantly lower expression than the calcium-signaling and sarcomere genes (Fig. S15F). These data support the conclusion that H2Bub1 accumulation near the center of calcium-signaling and sarcomeric genes in cardiomyocytes is UBE2B dependent and correlates with the amount of full-length transcripts.

We next asked whether this H2Bub1 accumulation is unique to human CMs. Previous literature identified H2Bub1 on cilia genes (which have an average length of 73 kb) in mouse oviducts (Robson et al., 2019), suggesting that this may be more generalizable. We thus analyzed previously published H2Bub1-ChIP-seq data of mouse ESCs and mouse embryonic fibroblasts (MEFs), and H2Bub1-ChIP-seq data of undifferentiated human fetal osteoblasts (hFOBs) and differentiated hFOBs (Najafova et al., 2017; Wang et al., 2021). We generated metagenes corresponding to MEF-specific genes [extra-cellular matrix (ECM) (*n*=130)] and hFOB-specific genes [epidermal growth factor related genes (EGF) (*n*=24)], with a median length within the 3rd quartile (median length is 48 kb for ECM and 99 kb for EGF) (Table S7). These metagenes show H2Bub1 accumulation near the center of the gene, which is higher in the MEFs and differentiated hFOBs, as expected (Fig. 5H,I). As in CMs, all the quantity and size-matched ‘random’ gene sets generated show no H2Bub1 accumulation near the center of the gene, as shown in a representative graph (Fig. S16G,H). The CM, MEF and hFOB data collectively suggest that H2Bub1 accumulation near the center of tissue-specific long genes is a general mechanism for regulating the amount of full-length transcripts, particularly in specialized tissues. In CMs, central H2Bub1 accumulation and transcription of complete transcripts of sarcomeric and calcium-signaling genes depend on UBE2B, providing a mechanism to explain the cardiac phenotype in the *Rnf20^fl/fl^*::*Nkx2.5*Cre mouse model.

## DISCUSSION

**Fig. 6.**
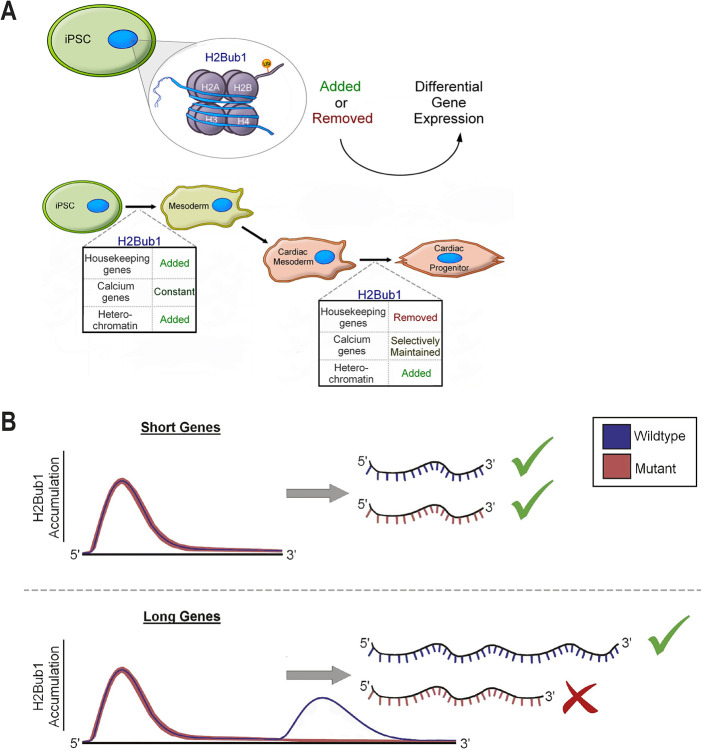
**Working model.** (A) During wild-type CM differentiation, housekeeping genes and heterochromatic regions have dynamic H2Bub1 levels. H2Bub1 is sparsely maintained during the transition from cardiac mesoderm to cardiac progenitor. Notably, selectively maintained genes are enriched for calcium-signaling genes. (B) Genes that are short in length have an increase in H2Bub1 at their 5′ end that decreases towards the 3′ end. However, longer tissue-specific genes have an accumulation in H2Bub1 near the center of the gene in wild-type cells but not in *UBE2B^−/−^*. In the mutants, when this accumulation is absent, the amount of full-length transcript is reduced.

Together, our data support a requirement for tight control of H2Bub1 levels in cardiogenesis. Decreased total H2Bub1 reduces the efficiency of CM differentiation *in vitro* and leads to reduced expression of calcium-signaling genes and structural abnormalities of the cardiac sarcomere, including a deficient H zone, *in vivo*. H2Bub1 is highly dynamic during human CM differentiation: during early stages, on housekeeping genes, the mark is increased, and at later stages it is decreased; the mark is selectively maintained on calcium-signaling genes in these later stages, occurring concordantly with a shift of H2Bub1 from euchromatic to heterochromatic regions (Fig. 6A). Finally, we show that the shape of the H2Bub1 mark changes between wild-type and *UBE2B^−/−^* CMs. The profile of H2Bub1 on short genes is as previously reported, with coverage over the entire gene body, higher at the 5′ end than the 3′ end of the gene (Jung et al., 2012). Conversely, long genes have a different profile: there is tissue-specific UBE2B-dependent H2Bub1 accumulation near the center of the gene, which correlates with amount of full-length transcripts (Fig. 6B).

We observed some notable differences between human patients and the mouse models. Most notably, individuals with CHD with haploinsufficiency of *RNF20* have major cardiac abnormalities, whereas mice have no observable phenotype (Robson et al., 2019). Mice are not perfect models for human disease, especially when modeling highly dosage-sensitive epigenetic modifications; even though there is extensive conservation between species, the lack of phenotype might be due to subtle species-specific differences that affect the phenotype (Hanna et al., 2018). The strength of the mouse model, however, is that it permits us to reduce the amount of RNF20 specifically in the heart and allows the mice to bypass early lethality. Each experiment alone suggested the role of an individual component (RNF20 in our mouse model and UBE2B in our iPSC-CM model) of the H2Bub1-deposition complex in cardiogenesis. It remains possible that the cardiogenesis phenotype in mice with cardiac-specific deletion of *Rnf20* is due to H2Bub1-dependent defect in cardiomyocyte differentiation/development coupled with a second, not yet understood, H2Bub1-independent function of RNF20 in another aspect of cardiogenesis. However, the convergence of the phenotype resulting from both cardiac-specific deletion of *Rnf20* in mice and the deletion of a second component of the H2Bub1-deposition complex, *UBE2B*, in human iPSC-derived cardiomyocytes on cardiomyocyte differentiation/development points strongly to a role for H2Bub1 in this process.

One mechanism to explain the phenotypic variability we observed between *Rnf20*^−/−^ mouse cardiomyocytes, *RNF20*^+/−^ humans and human iPSC-derived *RNF20*^+/−^ and *UBE2B*^−/−^ cardiomyocytes is that cardiomyocyte differentiation is exquisitely sensitive to H2Bub1 levels, which we propose are tightly regulated by the dose of the H2Bub1 deposition complex. Several previous observations also support epigenetic dose as a crucial developmental regulator. For example, haploinsufficiency for a broad range of chromatin regulators is one of the most significant contributors to human congenital heart disease (Zaidi et al., 2013), and dose regulation of H3K27me3 levels by altering levels of EED and JMJD3 have been shown to regulate the ratio of β-cell subtypes in the pancreas (Dror et al., 2023). One interesting future direction would be to see whether temporal inhibition could permit creation of *RNF20^+/−^* iPSC-derived CMs by circumventing their inability to form CMes. If the H2Bub1 dose and timing could be precisely titrated, these experiments could provide increased understanding of the role of the decrease in H2Bub1 during the transition from CP to CM.

Our data, in combination with previous H2Bub1 literature, suggest that the H2Bub1-deposition complex has multiple, distinct tissue- and time-specific functions. Focusing on heart development: at the left-right organizer stage, H2Bub1 is predicted to be located on cilia genes; then at the cardiogenesis stage, H2Bub1 is located on calcium-signaling genes; and finally, at the cardiac maturation stage, H2Bub1 is located on metabolic genes (Robson et al., 2019; VanDusen et al., 2021). These data support three separate functions for H2Bub1 in the heart: control of cardiac left-right asymmetry, establishing embryonic cardiac structure and function, and post-natal cardiomyocyte maturation; the first two of these have direct relevance to the mechanism underlying CHD and provide insight into the exceptionally broad range of CHD observed in individuals with genetic variants predicted to affect H2Bub1.

The observed dynamic levels and distribution of H2Bub1 during mouse heart development and hiPSC to CM differentiation support a model where tissue-specific transcriptional effects are determined by the retention, instead of deposition, of H2Bub1 on tissue-specific genes. At the level of individual genes, we observed H2Bub1 accumulation near the center of a subset of long genes, correlating with previous findings that RNA Pol II exhibits a similar accumulation near the center of all expressed genes (Fuchs et al., 2014; Young et al., 2011). We predict that H2Bub1 enhances transcriptional elongation of long tissue-specific genes. We hypothesize that this tissue specificity is achieved by H2Bub1 deposition occurring on highly expressed genes, due to changes in chromatin architecture. The genes that have this H2Bub1 accumulation and fewer full-length transcripts in response to mutations affecting the H2Bub1 deposition complex have significantly higher expression than the size- and quantity-matched random genes and all the 3rd quartile genes. We hypothesize that there are tissue-specific transcription factors that bind to the center of the gene and guide the H2Bub1-deposition complex to the correct location. The involvement of H2Bub1 in transcriptional elongation efficiency is supported by previous findings that long genes are more likely to depend on RNF20 to be induced upon differentiation into neuronal cells (Fuchs et al., 2012). Further evidence is the presence of H2Bub1 on long cilia genes in the multi-ciliated cells of oviducts (which require expression of long motile cilia genes), but not in the liver cells, which do not (Robson et al., 2019). Combined with our current data analyzing CMs, MEFs and hFOBs, we propose that targeted localized H2Bub1 accumulation is a more general mechanism that regulates tissue-specific transcriptional elongation efficiency on long genes, which is required for a subset of sarcomere and calcium-signaling genes during cardiogenesis.

The question remains of how H2Bub1 affects development of cardiac structure, as both individuals with variants affecting H2Bub1 and mouse embryos with cardiac-specific deletion of *Rnf20* have structural heart defects. A possible link to the observed structural heart defects in mice is that altered expression of calcium-signaling genes and abnormal sarcomeric structure observed in *Rnf20*^fl/−^::*Nkx2.5*Cre^+^ mouse embryos lead to defective cardiac function during embryonic development, and that the resulting hemodynamic derangement affects structural cardiac morphogenesis. Extensive evidence supports interdependence between embryonic hemodynamics and valve development, cardiac trabeculation, myocardial proliferation and formation of the epicardium (reviewed by Andrés-Delgado and Mercader, 2016). Genomic studies of individuals with CHD are beginning to provide further evidence of an overlap between genes classically linked to cardiomyopathy and individuals presenting with structural CHD. For example, dominant variants affecting myosin heavy chain 6 (*MYH6*) are associated with cardiomyopathy and atrial septal defects (Ching et al., 2005; Hershberger et al., 2010), whereas recessive variants in *MYH6* are found in 11% of individuals with Shone syndrome, which is characterized by valve defects and multiple levels of left ventricular obstruction (Jin et al., 2017). The shared role of H2Bub1 in CM differentiation and cardiogenesis in mouse and human provides further support for genetic overlap between cardiac structure development and myocardial function, and suggests that a subset of individuals with structural heart defects caused by genetic defects affecting cardiomyocytes may be more vulnerable to myocardial dysfunction. Although there are likely variations in the absolute H2Bub1 levels required for normal iPSC-derived CM development, and mouse embryo and human heart development, our observations in iPSC-derived CMs and mouse embryos indicate a shared requirement for the precise control of H2Bub1 in the heart.

## MATERIALS AND METHODS

### Mouse husbandry

The wild-type mouse data in Fig. S1 are on the CD1 background. The *Rnf20^+/−^*, *Rnf20^fl/fl^*, and all of the Cre lines used in this study are maintained on the C57BL/6 background. *Rnf20*^+/−^ were obtained from KOMP2 (Fig. S2A) (Valenzuela et al., 2003). *Rnf20^fl/fl^* mice were mated with actinCre to create *Rnf20^fl/−^* and mated with *Rnf20^+/−^* (KOMP2) to test that the two deletions were allelic. All Cre-expressing mice used were validated for correct expression and penetrance by testing their offspring with a ROSA^mT/mG^ mouse at E8.5 for GFP and tdTomato expression. Male *Rnf20^+/−^::Cre^+^* mice with validated Cre expression were mated to *Rnf20^fl/fl^* females. This research complies with all ethical regulations; mouse experiments were performed in a manner approved by the Yale University Institutional Animal Care and Use Committee.

### Cell lines and cardiomyocyte differentiation

PGP1 cells (a gift from the Seidman lab, Harvard Medical School, Boston, MA, USA) were maintained on Matrigel with mTESR media. Differentiation was performed in accordance with previously published work (Lian et al., 2013). Briefly, 0.5 million cells (or 0.7 million cells in the *UBE2B^−/−^* mutants) were plated on Matrigel about 4 days before the onset of differentiation. When the cells reached 100% confluency, differentiation was started using 12 µM of CHIR99021 in RPMI media supplemented with B-27 minus insulin. Twenty-four hours later, the media was replaced with RPMI media supplemented with B-27 minus insulin. Forty-eight hours later, the media was replaced with 5 µM of IWP4 in RPMI media supplemented with B-27 minus insulin. Forty-eight hours later, the media was replaced with RPMI media supplemented with B-27 minus insulin. On day eight and every 2-3 days after that, the media were replaced with RPMI media supplemented with B-27. After the onset of beating, the cardiomyocytes (CMs) were selected using 4 mM lactate in DMEM without glucose. A well was considered to be beating if two distinct regions on the plate contained beating cells. Before the collection of samples, mycoplasma testing was performed on all cell lines using the MycoAlert Plus Mycoplasma Detection Kit from Lonza (LT07-703).

As lactate selection is a metabolic selection, it can only work if the cells are CMs. Therefore, if a well was not beating, no lactate selection was carried out and the subsequent analyses were made on the non-selected cells.

### CRISPR-Cas9 editing of human iPSCs

Guides were created using the Massachusetts Institute of Technology's CRISPR tool to create mutations in *RNF20* and *UBE2B* (two each) near the transcriptional start site. These guides and the other necessary sequence were synthesized as gblocks and cloned using the TOPO TA cloning kit (Table S3). Two different protocols were used to create knockouts. *UBE2B^−/−^1*, *RNF20^+/−^1* and *RNF20^+/−^2* were created using the Lonza Nucleofector 2b with the Human Stem Cell Nucleofector Kit 2, according to the manufacturer's protocol. One-million cells were nucleofected using program B020 with 2 µg of a plasmid containing Cas9 (PX459, Addgene Adgene 62988) and 2 µg of a plasmid containing the sgRNA. *UBE2B^−/−^2* was created by adding 10 µg of Cas9 plasmid and 10 µg of sgRNA plasmid to 1 million cells using the Bio-Rad Gene Pulser XCell Eukaryotic System with a homemade nucleofection solution (5 mM KCl, 5 mM MgCl_2_, 15 mM Hepes, 102.94 mM Na_2_HPO_4_ and 47.06 mM NaH_2_PO_4_ monohydrate at pH=7.2) according to previously published work. The following parameters were used: 250 V, 500 µF and a 0.4 cm cuvette (Chen et al., 2015). These cells grew for 2 days with mTESR supplemented with 5 µM of Y27632. Selection for receiving the Cas9 plasmid then occurred for 2 days using 400 µg of puromycin. About 1 week after transfection, individual colonies were picked and screened for mutations using primers flanking the sgRNA (Table S1). If there was a possibility of a mixed colony, individual colonies were picked from that colony and these subcolonies were screened. This process was repeated until colonies were pure. Additionally, the DNA was cloned into TOPO TA cloning it (*RNF20*) or Zero Blunt TOPO Cloning Kit (*UBE2B*) and sequenced to verify the ratio of the two different alleles was 1:1.

#### Surrounding sequence to the gRNA

The sequence surrounding the gRNA is as follows: TGTACAAAAAAGCAGGCTTTAAAGGAACCAATTCAGTCGACTGGATCCGGTACCAAGGTCGGGCAGGAAGAGGGCCTATTTCCCATGATTCCTTCATATTTGCATATACGATACAAGGCTGTTAGAGAGATAATTAGAATTAATTTGACTGTAAACACAAAGATATTAGTACAAAATACGTGACGTAGAAAGTAATAATTTCTTGGGTAGTTTGCAGTTTTAAAATTATGTTTTAAAATGGACTATCATATGCTTACCGTAACTTGAAAGTATTTCGATTTCTTGGCTTTATATATCTTGTGGAAAGGACGAAACACC (Guide) GTTTTAGAGCTAGAAATAGCAAGTTAAAATAAGGCTAGTCCGTTATCAACTTGAAAAAGTGGCACCGAGTCGGTGCTTTTTTTCTAGACCCAGCTTTCTTGTACAAAGTTGGCATTA.

### Immunofluorescence of mice

Mouse embryos and hearts were fixed in 4% PFA overnight. They were placed in 30% sucrose until the tissue sank and then embedded in OCT. Section were cut at 12 µm. These slides were allowed to defrost and dry out for 3 h on a slide warmer. The sections were post-fixed for 10 min with 0.2% PFA, and then washed four times with PBS for 20 min. The sections were either blocked in 5% BSA, 10% goat serum, or Fish Gelatin Blocking agent in PBT (PBS+ 0.2% Triton X100). The samples were incubated with primary antibodies for either 4 h at room temperature or overnight (Table S2). The samples were washed four times in PBT for about 20 min, incubated with the corresponding chromophore-conjugated secondary antibody (Table S2) for about 45 min, and then washed four times in PBT for about 20 min. The second to last wash contained 1:2000 Hoechst. The samples were mounted on coverslips in ProLong Gold (Molecular Probes) and imaged on a Zeiss Observer Z1 equipped with Apotome optical interference imaging. Corrected total cell fluorescence (CTCF) was measured in ImageJ (Schneider et al., 2012).

### Immunofluorescence of cells

iPSCs and mesodermal (M) cells were passaged using 650 µl accutase per well in a six-well plate for 5-7 min at 37°C until the cells started to come off the plate. After spinning down at 200 ***g*** for 5 min, the cells were plated on coverslips. A 0.1% w/v solution of gelatin had been added to these coverslips overnight at 37°C. The next morning, these coverslips were rinsed with PBS, a 1/400 solution of Matrigel in media was added for 1 h, rinsed with PBS and then the passaged cells were added. The media the cells were grown in while they were on the coverslips was normal cell culture media for the respective stage with 5 µM of Y27632.

CMs were passaged using 2 ml Trypsin-EDTA (0.25%) added to each well of a six-well plate for 5 min at room temperature. After spinning down at 1000 rpm for 5 min, the cells were plated on coverslips. A 0.1% w/v solution of gelatin had been added to these coverslips overnight at 37°C. The next morning, these coverslips were rinsed with PBS and then the passaged cells were added. These cells were grown in RPMI20 (RPMI with 20% FBS) with 5 µM of Y27632 as per previous work (Lian et al., 2013).

After 2 days of culturing the iPSC, M or CMs on coverslips, they were fixed in 4% PFA for 10 min. Extreme caution was taken when adding and removing liquids in order not cause the cells to dissociate from the coverslips. The coverslips were rinsed four times for 20 min each with PBS. The sections were either blocked in 5% BSA or 10% goat serum in PBT (PBS+ 0.2% Triton X100). The samples were incubated with primary antibodies for either 4 h at room temperature or overnight (Table S2). The samples were washed four times in PBT for about 20 min each, incubated with the corresponding chromophore conjugated secondary antibody (Table S2) for about 45 min, and then washed four times in PBT for about 20 min. The second to last wash contained 1:2000 Hoechst. The coverslips were mounted on slides in ProLong Gold (Molecular Probes). The samples were imaged on the Zeiss Observer Z1 equipped with Apotome optical interference imaging.

### Histology

Whole embryos or adult hearts were fixed overnight in 4% buffered paraformaldehyde. The adult hearts were then cut in half sagittal sections. These embryos or half hearts were dehydrated, cleared and embedded in paraffin wax. Transverse sections (embryonic sections were 10 µm and adult heart sections were 3-6 µm) were cut using a microtome and were stained with Hematoxylin and Eosin following standard protocols. Images were taken on a Zeiss Axiovert microscope, using an Axiocam driven by Axiovision software. Measurements (heart size, interventricular septum length and compact myocardium thickness) were made using imageJ (Schneider et al., 2012). Compact myocardium thickness was determined by averaging three measurements of the compact myocardium.

### Transmission electron microscopy

The embryos were dissected in 4% PFA. A small hole was made in an atrium with a scalpel to allow the fixative to reach the inside the heart. Embryonic heart tissue was fixed in 2.5% glutaraldehyde and 2% paraformaldehyde in 0.1 M sodium cacodylate buffer (pH 7.4) for 1 h at room temperature and left at 4°C for 2 days. The samples were rinsed in buffer, then post fixed in 1% osmium tetroxide and en-bloc stained in 2% aqueous uranyl acetate for another 1 h. This was followed by rinsing in distilled water and dehydrating in an ethanol series, then resin infiltration in Embed 812 (Electron Microscopy Sciences) and baking overnight at 60°C. Hardened blocks were cut using a Leica UltraCut UC7. 60 nm sections were collected on formvar/carbon-coated grids and contrast stained using 2% uranyl acetate and lead citrate. The sections were viewed in an FEI Tencai Biotwin TEM at 80 kV. Images were taken using Morada CCD and iTEM (Olympus) software.

### Protein extractions

Mouse hearts were dissected and the blood was removed by flushing with PBS. The hearts were flash frozen in liquid nitrogen and homogenized on Mikro Dismembrator II. Cells were removed from the plates using accutase (for iPSC and M cells) or Trypsin-EDTA 0.25% (for CMs). Proteins were extracted using NE-PER Nuclear and Cytoplasmic extraction reagents (Thermo Fisher Scientific 78833) supplemented with proteinase inhibitors (Roche) according to the manufacturer's instructions. After the fractions were acquired, they were combined. The proteins were analyzed by western blotting.

### Histone extractions

Histones were extracted from the pellets from the protein extractions using the total histone extraction kit (Epigentek) according to manufacturer instructions. The extracted histones were analyzed by western blotting.

### Western blots

The protein samples were evenly loaded onto 4-12% gradient Bis-Tris gels (Invitrogen) (UBE2B) or 3-8% Tris-Acetate gels (NuPAGE) (RNF20 and RNF40), transferred to PVDF membrane, blocked overnight in 5% dry milk in TBST, and incubated with primary antibodies overnight rocking at 4°C (Table S2), followed by washes in TBST for 1 h, incubated with corresponding HRP-conjugated secondary antibodies (Abcam, ab6721; 1:20,000) for 1 h at room temperature, washed in TBST for 4.5 h. Images were acquired using film and scanned in to be quantified using imageJ (Schneider et al., 2012). For a loading control for the wild-type mice over time (Fig. S1C), we used a Mini-PROTEAN TGX Stain-Free precast gel (Bio-Rad). Total proteins were analyzed with ImageLab (Bio-Rad). For a loading control for the iPSCs, we used GAPDH (Fig. S11B).

The histone samples were evenly loaded onto 4-12% gradient Bis-Tris gels (Invitrogen), transferred to PVDF membrane, blocked for 1 h in 5% dry milk in TBST and incubated with primary antibodies overnight rocking at 4°C (Table S2). The next day, the blots were washed in TBST three times for 15 min each, incubated with corresponding HRP-conjugated secondary antibodies (Abcam, ab6721; 1:20,000) for 1 h at room temperature, washed in TBST three times for 15 min each. Images were acquired using film and scanned in to be quantified using imageJ (Schneider et al., 2012). Expression was normalized to levels of H2B.

### ChIP-seq

Samples were collected in triplicate, where each replicate came from a plate that started differentiation at a different time. Samples were harvested by adding 2 ml of 1% formaldehyde to the media for 10 min. The reaction was quenched by adding 664.0625 mM of glycine for 5 min. Two washes with ice-cold PBS were carried out before scraping the cells of the plate in PBS using a cell scraper. The tube was spun down at 450 ***g*** for 5 min and flash frozen in liquid nitrogen. The frozen samples were resuspended in 1 ml lysis buffer (10 mM Tris, 2 mM EDTA and 0.1% SDS) for 30 min on ice and one to six wells of a six-well plate (depending on the stage and how many cells were in each well) from one batch of cells were combined in the same tube. The DNA quantity was estimated by using a Qiagen PCR purification kit and the volumes were normalized to 6.25 ng/µl. The cells continued to lyse overnight. The samples were sonicated on a Bioruptor Pico (23 cycles of 30 s on and 30 s off) in 0.65 ml tubes with 120 µl of sample in each tube. The DNA quantity was adjusted to 1 µg per sample, diluted two parts dilution buffer to one part DNA with a dilution buffer, and incubated with 4 µl of H2Bub1 antibody or Igg antibody overnight (dilution; supplier, catalogue code Table S2). The sample was then incubated with 50 µl of protein G Dynabeads (Invitrogen) for 4 h at 4°C while rocking. The beads were washed twice with low-salt buffer, twice with high-salt buffer, twice with LiCl buffer and twice with TE buffer (changing tubes after the first TE wash). Each wash was 3 min long. The immunoprecipitated chromatin was eluted from the beads in 85 µl of elution buffer twice for 15 min at 70°C. The immunoprecipitated chromatin was then treated with RNase, underwent reverse cross-linking and was treated with proteinase K at 65°C overnight. The DNA was purified using a Qiagen PCR purification kit. qPCR was performed on these samples to verify that the immunoprecipitation worked using Quantabio PerfeCta syberGreen mix. The samples were run on either a 7900HT Fast Real-Time PCR Detection System (Applied Biosystems) or a CFX96 Touch Real-Time (BioRad) in triplicate. The results were analyzed by calculating percent input. The samples that passed quality control were sequenced at Yale Center for Genome Analysis using NovaSeq.

Buffers used were are follows: dilution buffer, 16.7 mM Tris (pH=8), 167 mN NaCl, 1.2 mM EDTA, 0.002% SDS, 1.1% Triton, 1× proteinase inhibitors (Roche) and 5 mM Na butyrate; low-salt buffer, 20 mM Tris (pH=8), 0.1% SDS, 1% Triton, 2 mM EDTA and 150 mM NaCl; high-salt buffer, 20 mM Tris (pH 8.0), 0.1% SDS, 1% Triton, 2 mM EDTA and 500 mM NaCl; LiCl buffer, 10 mM Tris (pH 8), 250 mM LiCl, 1 mM EDTA, 1% NP40 and 1% Na-deoxycholate; TE buffer, 10 mM Tris (pH=8) and 1 mM EDTA; elution buffer, 50 mM Tris (pH 8), 1 mM EDTA and 1% SDS.

### ChIP-seq analysis

The ChIP-seq reads were trimmed using the FASTX-Toolkit to remove poor-quality reads and were aligned to the human reference genome (hg38) using the BWA Mem algorithm (version 0.7.17). To call peaks, Model-based Analysis of ChIP-seq (MACS) (version 2.1.1.20160309) was used, where the input was used as the control, and reads were normalized using fold enrichment (Zhang et al., 2008). As H2Bub1 peaks are atypical, the BAMPE option, the no model option and the broad option (with a cutoff of 0.2) were used. The presence of H2Bub1 near the TSS (−2 kb, +10 kb) was computed using ‘computeMatrix’ in deepTools (version 2.5.0.1) and the genes were divided into four clusters using kmeans clustering based on the iPSC peaks (Fig. 3A,B) or the wild-type peaks (Fig. S13C) (Ramírez et al., 2016). Four clusters were selected unbiasedly by implementing the elbow method in Python (version 2.7.13). The plots that were generated in deepTools represent the average of the three replicates. To determine the differentially bound sites between samples, the ‘DiffBind’ package in R (version 3.6.1) was used with the following parameters: maximum q value of 0.05 and minimum absolute fold change of 1.5 (https://www.r-project.org/; http://bioconductor.org/packages/release/bioc/vignettes/DiffBind/inst/doc/DiffBind.pdf). This package takes the three replicate peak files as input, so it considers the spread of the data, not only the average. These differentially bound sites were matched to the nearest gene using the ‘ChIPseeker’ package in R (UCSC hg38) (https://www.r-project.org/; Yu et al., 2015). Importantly, a gene was only considered differentially bound (between the mutants and the wild-type cells) if there was a differentially bound region in the gene in both mutants. Additionally, a gene was considered significant only if it was observed in at least two out of the three replicates. The heat maps were made using the ‘pheatmap’ package in R (https://CRAN.R-project.org/package=pheatmap; https://www.r-project.org/). The Venn diagrams were made using the R package VennDiagram (Chen and Boutros, 2011; https://www.r-project.org/). GO enrichment analysis was carried out in DAVID (version 6.8) (Huang et al., 2009a,b). The gene traces were visualized using the UCSC genome browser (Kent et al., 2002).

### RNA-seq

Samples were collected in triplicate, where each replicate came from a plate that started differentiation at a different time. Each replicate consisted of two wells of a six-well plate that were differentiated at the same time. Total RNA was extracted using a Qiagen RNeasy mini kit with the RNase-Free DNase Set. The mRNA-seq libraries were prepared using a KAPA mRNA HyperPrep Kit (08098123702). Samples were sequenced at Yale Center for Genome Analysis using NovaSeq.

### RNA-seq analysis

The RNA-seq reads were trimmed using the FASTX-Toolkit to remove poor-quality reads and were aligned to the human reference genome (hg38) using Kallisto (version 0.45.0) in R (version 3.6.1) (Bray et al., 2016; https://www.r-project.org/). Differential expression analysis was carried out using the Wald test in Sleuth (version 0.30.0) in R with the following parameters: maximum q value of 0.05 and minimum absolute fold change of 1.5 (Pimentel et al., 2017; https://www.r-project.org/). BiomaRt (version 3.13) was used to match the transcripts with genes (Durinck et al., 2005, 2009; https://www.r-project.org/). As with ChIP-seq, a gene was only considered to be differentially expressed (between the mutant and the wild-type cells) if there was a differentially expressed transcript for the gene in both mutants. The heat maps were made by selecting the most differentially expressed regions in each GO term using the ‘pheatmap’ package in R (https://cran.r-project.org/web/packages/pheatmap/index.html; https://www.r-project.org/). GO enrichment analysis was caried out in DAVID (version 6.8) (Huang et al., 2009a,b). The gene traces were visualized in the Integrative Genomics Viewer (version 2.4.5) (Thorvaldsdottir et al., 2013).

### Analysis of compartment data

H2Bub1 peaks that overlapped by greater than or equal to 20 positions to a compartment interval, based on previously collected compartment data, were considered to be part of that interval (Bertero et al., 2019). Intervals that had a positive value were in the ‘A’ compartment, and intervals that had a negative value were in the ‘B’ compartment. The three ChIP-seq replicates were compared with the compartment data separately and then mean and s.e.m. were calculated.

To calculate expected values, 1000 bootstrap replicates were created for each stage of CM differentiation. A bootstrap replicate was created by sampling the compartment file with replacement until the bootstrap replicate file had the same number of rows as the H2Bub1 peak file. By doing this, we generated a ‘random’ collection of intervals from the ‘A’ and ‘B’ compartment that was of the same length as the H2Bub1 peak files. The mean and s.e.m. were calculated on these files to get the expected values.

To calculate *P*-values between observed and expected, the following equation was used for each replicate: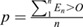, where *E* is the expected percentage, *O* is the observed percentage and *n* is the bootstrap replicate number. A sample was considered significant if every replicate had a *P*-value of less than 0.05.

To calculate *P*-values between observed values of different stages, the following equation was used to calculate a z-score between these two percentages: 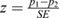, where *p*_1_ and *p*_2_ are the percentages and *SE* is the standard error. The z distribution was used to calculate a *P*-value from the z-score. All of this analysis was carried out in R (version 3.6.1) (https://www.r-project.org/).

### Analysis of histone marks

H3K27me3 and H3K4me3 peaks were downloaded from the Encode Consortium and generated by the Bernstein lab (ENCODE Project Consortium, 2012; Luo et al., 2020) (Table S4). Previously collected data on other histone marks from iPSC and CM stages, as well as the H2Bub1 data from the iPSC and CM stages, were matched to the nearest gene using the ‘ChIPseeker’ package in R (UCSC hg38) (ENCODE Project Consortium, 2012; Luo et al., 2020; https://www.r-project.org/; Yu et al., 2015). Table S4 indicates which ENCODE files were used. For each replicate of every mark at each stage, the presence or absence of a peak in a gene was recorded in a data frame (1 indicated presence; 0 indicated absence). A 2×2 contingency table was created to record how many genes contained both marks, how many genes had one mark and how many genes had neither mark.

To calculate expected values, fixed row and column sums were used. For example, in a 2×2 contingency table, the expected value of position (2,2) is 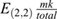, where *m* is the column sum and *k* is the row sum (Fu and Adryan, 2009). The graph was made by plotting the mean ratios of the observed values to the expected values. A χ^2^ test was carried out to calculate *P*-values between observed values and expected values. *P*-values comparing observed values between stages were calculated using an unpaired two-tailed, heteroscedastic *t*-test. All analyses were carried out in R (version 3.6.1) (https://www.r-project.org/).

### Mouse calcium qRT-PCR

E11.5 mouse hearts were dissected and flash frozen in liquid nitrogen separately. After genotyping, 10 hearts were pooled together to form each sample. RNA was extracted with the Qiagen RNeasy Plus mini kit. cDNA was synthesized with Quantabio qScript cDNA SuperMix. qPCR was carried out using Quantabio PerfeCta syberGreen mix on a CFX96 Touch Real-Time (BioRad) in biological duplicates and technical triplicates. See Table S1 for primers. Expression was normalized to the ribosomal protein *18s.*

### Movies

Movies of the CM stage cells were produced using bright-field imaging on a Zeiss Observer Z1. We collected 50 frames, one per second.

### Metagene analysis

We first assembled a list of regions. We decided to focus on calcium-signaling genes and sarcomeric genes, as those are the genes that are differentially expressed between the ChIP-seq and RNA-seq data. We combined the sets of genes that were selectively maintained and differentially expressed into two bed files (one for calcium-signaling genes and one for sarcomeric genes). We used the R package metagene2 (version 1.8.0) to create the plots with the following parameters: normalize with RPM, alpha=1 and paired_end is true (https://www.bioconductor.org/packages/release/bioc/html/metagene2.html; https://www.r-project.org/).

To generate a ‘random’ set of genes, we fit the lengths of the genes in the calcium-signaling bed file and the sarcomeric gene bed file to the poisson function in R (version 3.6.1) (https://www.r-project.org/). We then generated random numbers (the same number as there are lines in each bed file) from that distribution. For each random number, we found all the downregulated genes in *UBE2B^−/−^1* that are within 10% of that number. We then kept picking a gene at random until one was downregulated in both mutants. We repeated the procedure for upregulated and non-regulated genes. We created 10 sets of each (upregulated calcium-signaling, downregulated calcium-signaling, non-regulated calcium-signaling, upregulated sarcomeric genes, downregulated sarcomeric genes and non-regulated sarcomeric genes). To verify that there was no accumulation near the center, these 60 graphs were manually inspected.

To determine whether the calcium-signaling genes, the sarcomeric genes and the downregulated genes in the *UBE2B^−/−^* cells are longer than by chance, we created 10,000 random quantity-matched transcript sets, as described above. We calculated the *P*-value by determining the fraction of random gene sets that had a mean length greater than the corresponding gene set.

### Full-length transcripts

To calculate the amount of full-length transcripts, we used the R package CoverageView (version 1.30.0) (https://bioconductor.org/packages/release/bioc/html/CoverageView.html; https://www.r-project.org/). We modified the source code of draw.heatmap to produce a pdf file to make it compatible with the machine it was being run on. We used 100 windows, normalized with RPM and presented the graph as a log2ratio. We produced one graph per replicate per mutant. As all of the replicates look similar and both mutants look similar, we show one representative image. We used the same set of ‘random’ genes generated for the metagene analysis. We manually inspected the 360 resulting graphs (ten replicates of ‘random’ genes) for three classes of genes (upregulated, downregulated and non-regulated), for two bed files (calcium-signaling genes and sarcomeric genes) and for three replicates of two mutant cell lines. The gene traces were visualized in the Integrative Genomics Viewer (version 2.4.5) (Thorvaldsdottir et al., 2013).

### mESC/MEF and hFOB analysis

We downloaded the raw data from previously published work and put it through our ChIP-seq and RNA-seq pipelines described in the ChIP-seq and RNA-seq analysis sections (Najafova et al., 2017; Wang et al., 2021). To determine the tissue-specific genes, we used the Wald test in Sleuth (version 0.30.0) (maximum q value of 0.05 and minimum absolute fold change of 1.5) to compare the wild-type MEFs with the wild-type mESCs and the undifferentiated hFOBs to the differentiated hFOBs (Pimentel et al., 2017; https://www.r-project.org/). BiomaRt was used to match the transcripts with genes (Durinck et al., 2005, 2009; https://www.r-project.org/). GO enrichment analysis was carried out using DAVID (version 6.8) on the genes upregulated in the MEFs compared with the mESCs and the genes upregulated in the differentiated hFOBs compared with the undifferentiated hFOBs (Huang et al., 2009a,b). The GO terms for the MEFs were related to ECM and the GO terms for the hFOBs were related to EGF-related genes. Thus, we created metagenes for genes in those categories following the pipeline described in the metagene section. As this group did not knock out a component of the H2Bub1-deposition complex, we did not think it was necessary to create ‘random’ genes representing the upregulated, downregulated or non-regulated genes. Therefore, we created 20 ‘random’ gene sets for ECM and 20 ‘random’ gene sets for EGF-related genes.

### Statistics and reproducibility

Graphs and statistics were carried out either in R (version 3.6.1) or Prism (version 9.1.0) (https://www.r-project.org/). All experiments were carried out in replicate, with the number clearly indicated on the figure or in the figure legend. All statistical tests are defined in the figure legend and any non-traditional tests are described in detail in the appropriate methods section. Multiple comparison adjustments were carried out using the Bonferroni method. Biological replicates are samples analyzed from distinct samples. To account for off-target effects due to CRISPR, we created two cell lines for each mutant. For mouse experiments, as multiple mice were pooled together into a replicate, both sexes were mixed together in each sample. Additionally, littermate controls were used. The iPSC experiments were all carried out in a male cell line.

## Supplementary Material

10.1242/develop.201899_sup1 Supplementary information

Table S5. ChIP-seq wild-type iPSC-derived cardiomyocytes

Table S6. RNA-seq wild-type iPSC-derived cardiomyocytes

Table S7. ChIP-seq UBE2B^−/−^ cardiomyocytes

Table S8. RNA-seq UBE2B^−/−^ cardiomyocytes
