# Supplementary material for: The H2Bub1-deposition complex is required for human and mouse cardiogenesis
Source: Development. 2023 Dec 1;150(23):dev201899. doi: 10.1242/dev.201899 (PMC10730087; doi:10.1242/dev.201899)
Supplement: Supplementary information [file develop-150-201899-s1.pdf]

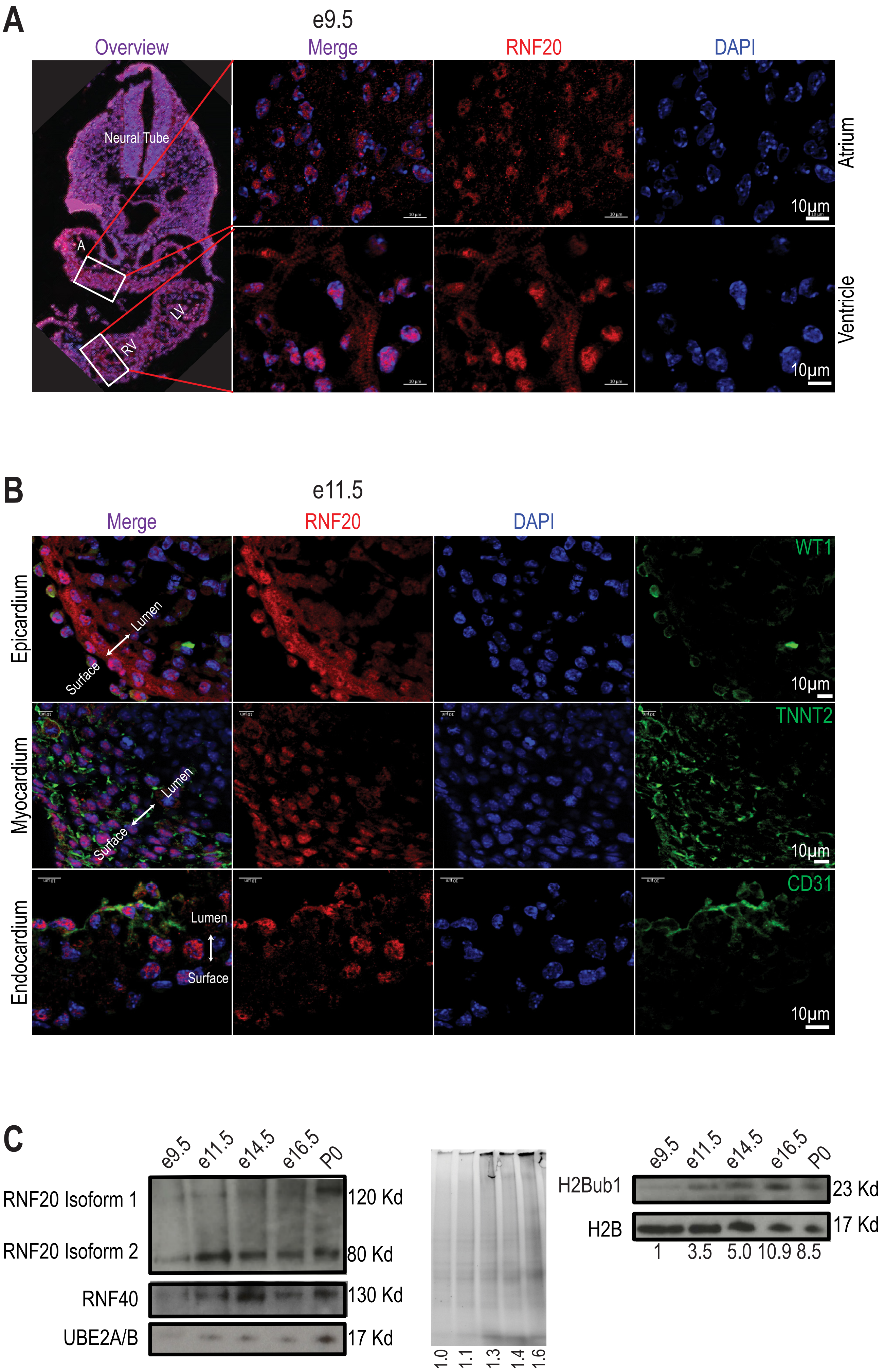

Fig. S1. RNF20 is dynamically expressed during mouse heart development. (A) Immunofluorescent staining for RNF20 in different sections (atrium and ventricle) of the e9.5 wild-type mouse heart. A – atrium, LV – left ventricle, RV – right ventricle. (B) Immunofluorescent staining for RNF20 and cell type markers (Epicaridum, WT1; Myocardium, TNNT2; Endocaridum, CD31) in e11.5 wild-type mouse hearts. (C) Western blot for H2Bub1-deposition complex components (RNF20, RNF40, and UBE2B) and H2Bub1 in wild-type mouse hearts (e9.5, e11.5, e14.5, e16.5, and P0). The loading control for the complex components is total protein (shown next to the western blots with relative intensities given underneath) and for H2Bub1 is H2B.

Development • Supplementary information

A

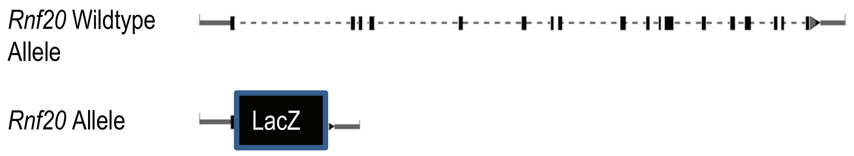

B

	Rnf20$^{+/-}$
	P0
+/+	33.1% (346/1045)
+/-	66.9% (699/1045)
-/-	0% (0/1045)

Fig. S2. Phenotype of *Rnf20*$^{-/-}$ mutants. (A) Diagram illustrating the *Rnf20* deletion (KOMP2). This mouse replaces the coding region of Rnf20 with LACZ. (B) Percent of each genotype of P0 mice born from the cross of two *Rnf20*$^{+/-}$ mice.

Development • Supplementary information

A

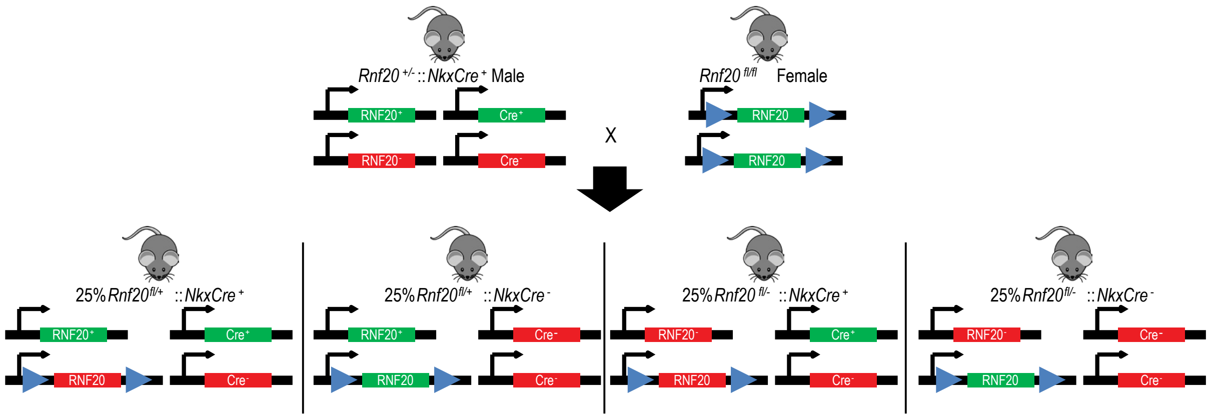

B

$Rnf20^{+/-}$::$Nkx2.5Cre^{+}$ X $Rnf20^{fl/fl}$					
	e9.5	e11.5	e12.25	e12.5	P0
fl/+ cre+	35% (7/20)	25.6% (46/180)	20.5% (34/166)	55% (11/20)	35% (21/60)
fl/+ cre-	10% (2/20)	27.8% (50/180)	35.5% (59/166)	25% (5/20)	43.3% (26/60)
fl/- cre+	25% (5/20)	26.7% (48/180)	20.5% (34/166)	**5% (1/20)**	**0% (0/60)**
fl/- cre-	30% (6/20)	20% (36/180)	23.5% (39/166)	15% (3/20)	21.7% (13/60)

C

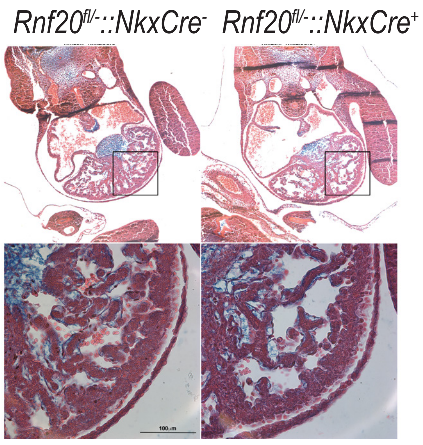

D

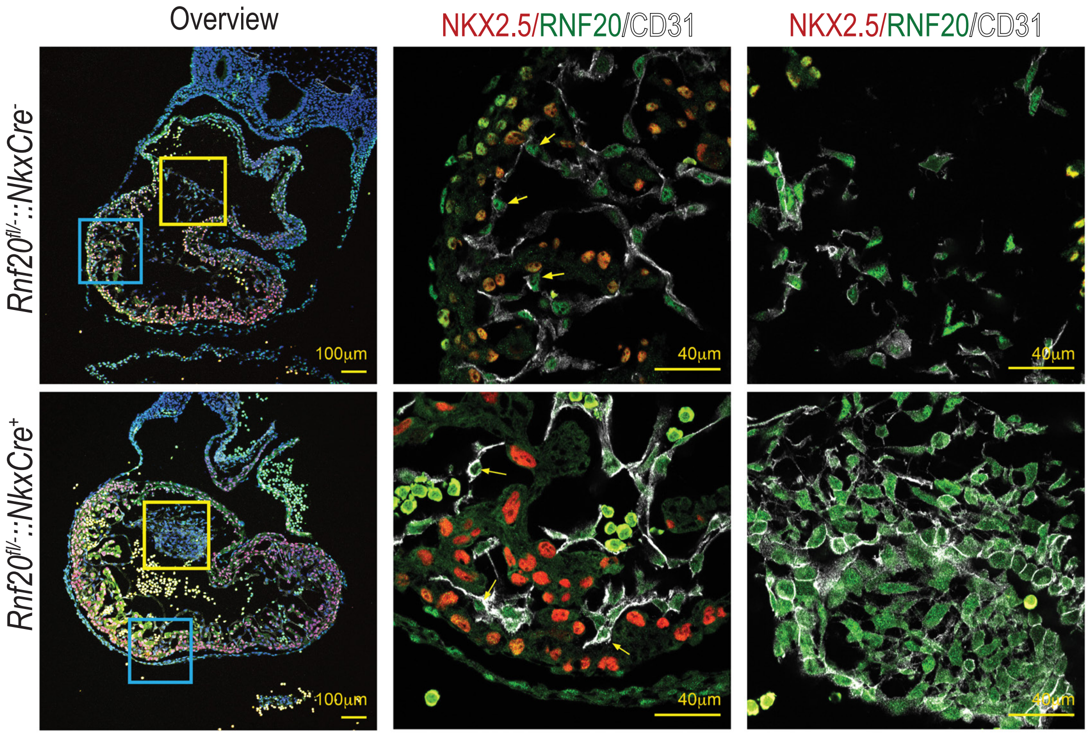

Fig. S3. Breeding strategy and phenotype of *Rnf20* conditional mutants (*Nkx2.5*Cre). (A) Diagram illustrating the mouse breeding scheme for the conditional knockout mice. (B) Percent of each genotype of e9.5, e11.5, e12.25, e12.5, and P0 from the cross of *Rnf20*$^{fl/fl}$ and *Rnf20*$^{+/-}$ ::*Nkx2.5*Cre^{+}. (C) Example hematoxylin and eosin stained e11.5 wild-type (*Rnf20*$^{fl/-}$::*Nkx2.5*Cre^{-}) and mutant (*Rnf20*$^{fl/-}$::*Nkx2.5*Cre^{+}). Lower panels are higher magnification views of the ventricles. (D) Immunofluorescent staining for NKX2.5 (red), RNF20 (green), and CD31-endocardium (white) in e10.5 wild-type (*Rnf20*$^{fl/-}$::*Nkx2.5*Cre^{-}) and mutant (*Rnf20*$^{fl/-}$::*Nkx2.5*Cre^{+}) mouse hearts. Corrected Total Cell Fluorescence (CTCF) for RNF20 in the compact myocardium is given.

Development • Supplementary information

A

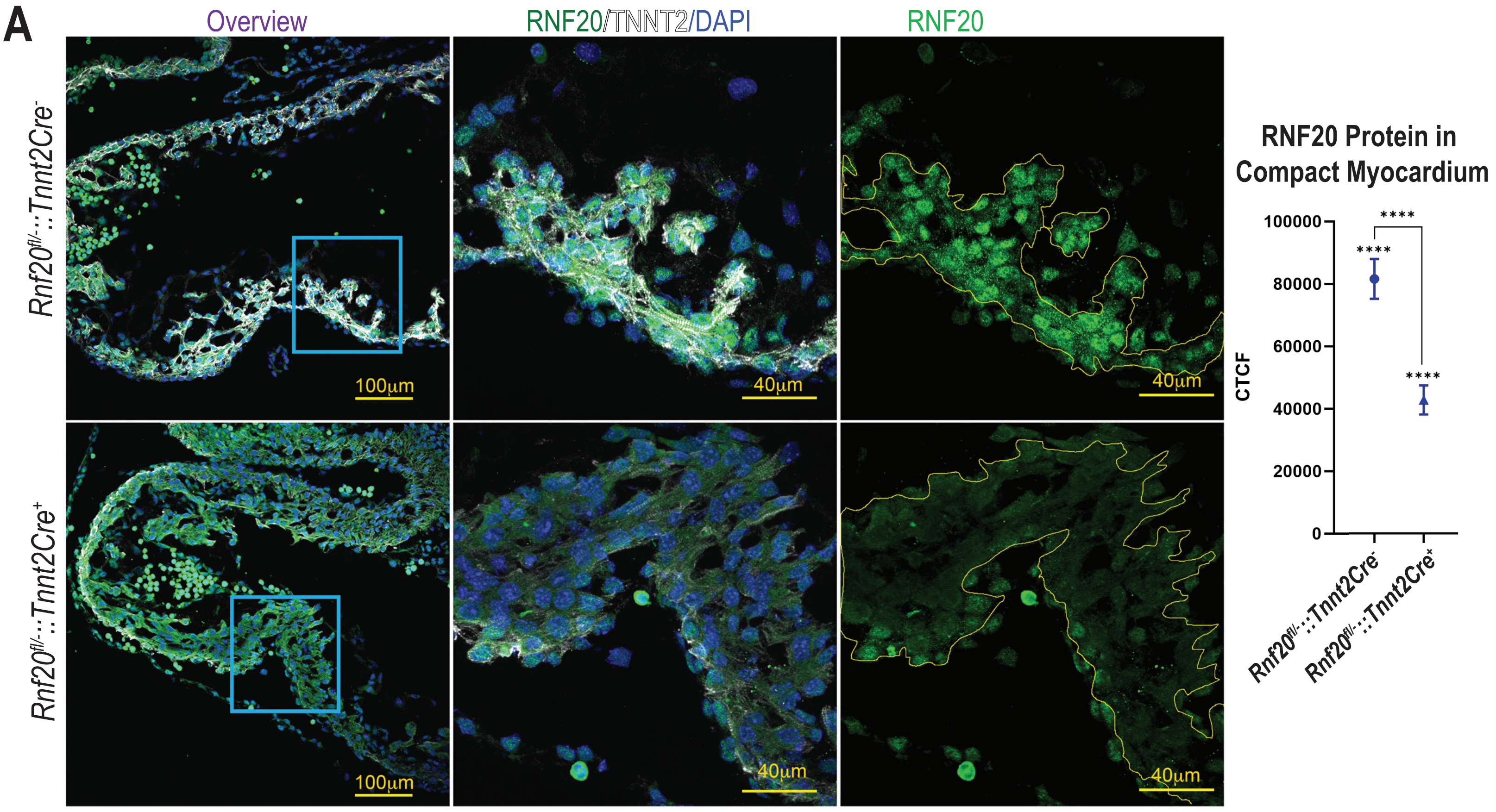

B

	$Rnf20^{+/-}$::$Tnnt2Cre^{+}$ X $Rnf20^{fl/fl}$			
	e12.5	e14.5	e15.5	P0
fl/+ cre+	50% (3/6)	41.3% (19/46)	50% (12/24)	36.8% (21/57)
fl/+ cre-	0% (0/6)	10.9% (5/46)	0% (0/24)	14% (8/57)
fl/- cre+	33.3% (2/6)	**10.9% (5/46)**	**0% (0/24)**	**0% (0/57)**
fl/- cre-	16.7% (1/6)	37% (17/46)	50% (12/24)	49.1% (28/57)

C

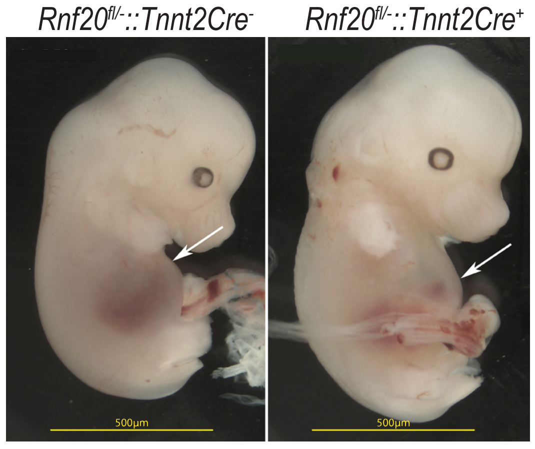

D

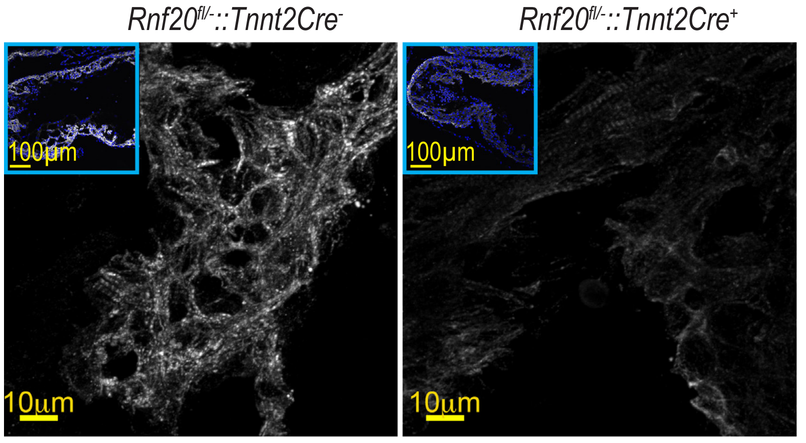

Fig. S4. Phenotype of *Rnf20* conditional mutants (*Tnnt2*Cre). (A) Immunofluorescent staining for TNNT2 (white) and RNF20 (green) in e10.5 wild-type ($Rnf20^{fl/+}$::*Tnnt2*Cre^{+}) and mutant ($Rnf20^{fl/-}$::*Tnnt2*Cre^{+}) mouse hearts. A – atria, LV – left ventricle , RV – right ventricle. Corrected Total Cell Fluorescence (CTCF) for RNF20 in the compact myocardium is given. (B) Percent of each genotype of e12.5, e14.5, e15.5, and P0 from the cross of $Rnf20^{fl/fl}$ and $Rnf20^{+/-}$ ::*Tnnt2*Cre^{+}. (C) Pictures of e14.5 wild-type ($Rnf20^{fl/+}$::*Tnnt2*Cre^{+}) and mutant ($Rnf20^{fl/-}$::*Tnnt2*Cre^{+}) mouse embryos. (D) Immunofluorescent staining for TNNT2-myocardium (white) in e10.5 wild-type ($Rnf20^{fl/-}$::*Tnnt2*Cre^{-}) and mutant ($Rnf20^{fl/-}$::*Tnnt2*Cre^{+}) mouse hearts. The same embryos are depicted in Figures S4a and S4d.

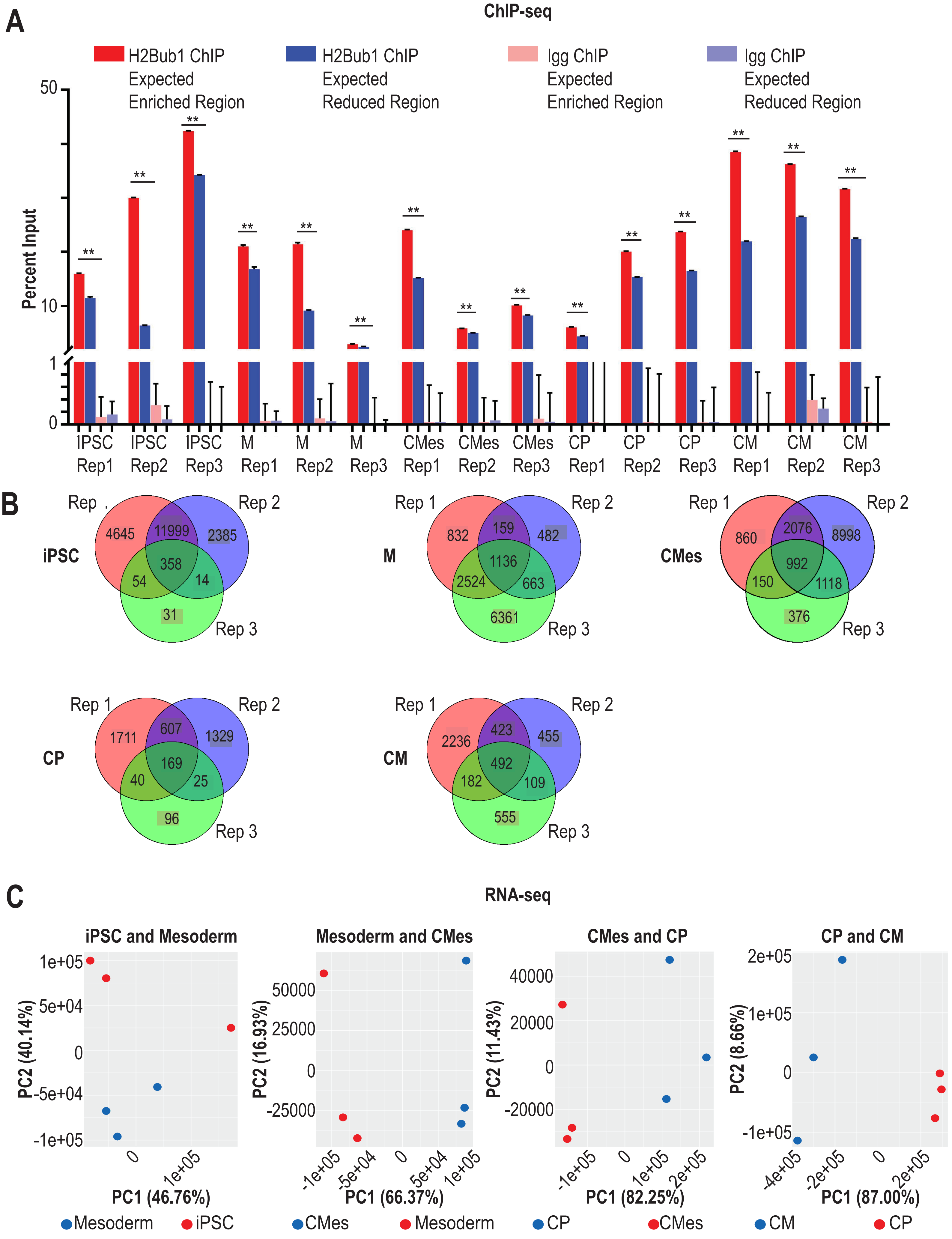

Development • Supplementary information

Fig. S5. WT ChIP-seq and RNA-seq Quality Control. (A) Percent input for a region that is known to be enriched in H2Bub1 (GAPDH promoter) and in a region that is known to be reduced in H2Bub1 (GAPDH gene body) for all of the H2Bub1 and Igg ChIP experiments. Data are shown as mean ± SD (n = 3). Dark red represents H2Bub1 ChIP in the expected enriched region (GAPDH promoter), dark blue represents H2Bub1 ChIP in the expected reduced region (GAPDH gene), light red represents Igg ChIP in the expected enriched region (GAPDH promoter), and light blue represents Igg ChIP in the expected reduced region (GAPDH gene). Unpaired 1-tailed t-test, ** $P < 0.01$. (B) Common and unique genes with peak calls across the three replicates (Replicate 1 is in red, replicate 2 is in purple, and replicate 3 is in green) in five stages of CM differentiation (iPSC, mesoderm (M), cardiac mesoderm (CMes), cardiac progenitor (CP), and cardiomyocyte (CM)). (C) PCA plots of RNA-seq experiments of CM differentiation comparing all neighboring stages (iPSC (red) v mesoderm (M) (blue), M (red) v CMes) (blue), CMes (red) v CP (blue), and CP (red) v CM (blue)) for three replicates of each stage.

Development • Supplementary information

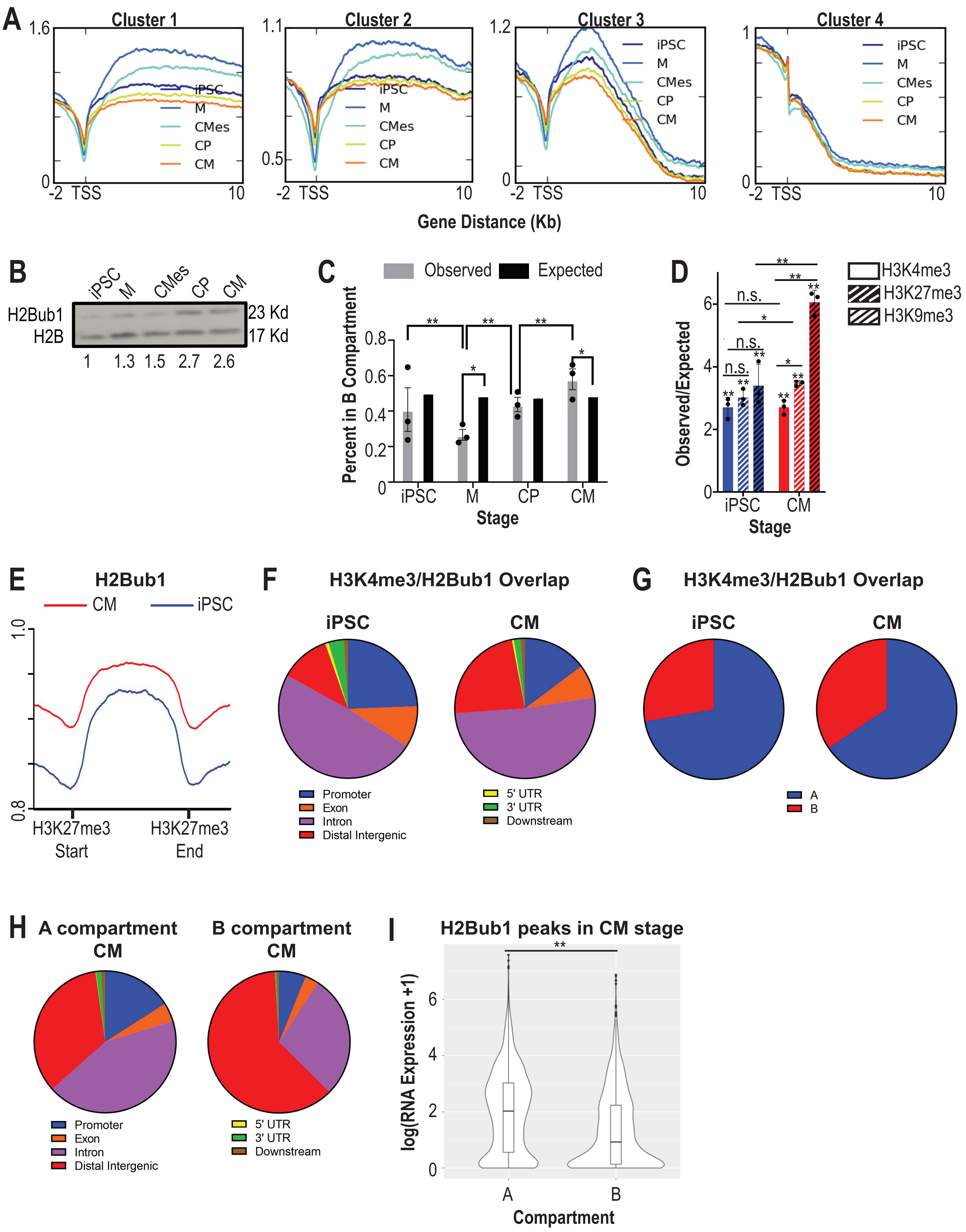
A
Cluster 1
Cluster 2
Cluster 3
Cluster 4
iPSC
M
CMes
CP
CM
-2 TSS 10
Gene Distance (Kb)
B
iPSC M CMes CP CM
H2Bub1
H2B
23 Kd
17 Kd
1 1.3 1.5 2.7 2.6
C
Observed
Expected
Percent in B Compartment
iPSC M CP CM
Stage
D
H3K4me3
H3K27me3
H3K9me3
Observed/Expected
n.s.
iPSC CM
Stage
E
H2Bub1
CM
iPSC
H3K27me3 Start
H3K27me3 End
F
H3K4me3/H2Bub1 Overlap
iPSC
CM
Promoter
Exon
Intron
Distal Intergenic
5' UTR
3' UTR
Downstream
G
H3K4me3/H2Bub1 Overlap
iPSC
CM
A
B
H
A compartment CM
B compartment CM
Promoter
Exon
Intron
Distal Intergenic
5' UTR
3' UTR
Downstream
I
H2Bub1 peaks in CM stage
log(RNA Expression +1)
A
B
Compartment

Fig. S6. H2Bub1 is located in the heterochromatic regions. (A) Graphs of the four clusters identified in Figure 3A across five stages of CM differentiation (iPSC, Mesoderm (M), Cardiac Mesoderm (CMes), Cardiac Progenitor (CP), and Cardiomyocyte (CM)). Cluster 1 indicates high H2Bub1 levels, cluster 2 indicates low H2Bub1 levels, cluster 3 indicates moderate H2Bub1 levels, and cluster 4 indicates no H2Bub1. (B) Western blot for H2Bub1 in five stages of CM differentiation (iPSC, M, CMes, CP, and CM). The loading control for H2Bub1 is H2B. Numbers indicate average quantification (imageJ) of H2Bub1 normalized to H2B over two replicates. (C) Percent of H2Bub1 peaks overlapping with the B compartment in each stage of CM differentiation (iPSC, M, CP, and CM) comparing observed (gray) and expected (black) values. Data are shown as mean ± SEM ($n = 3$). P-values comparing observed and expected values were manually calculated by comparing the actual values to the expected values, * $p < 0.05$, ** $P < 0.01$ (see Materials and Methods). P-values comparing observed values between stages were calculated by computing a z score, * $p < 0.05$, ** $P < 0.01$. (D) Percent of H2Bub1 peaks on the same gene as an activating chromatin mark (H3K4me3 shown in solid bars) and two repressive chromatin marks (H3K27me3 shown in bars with white dashes and H3K9me3 with black dashes) in iPSCs and CMs. Data are the ratio of observed to expected values. Data are shown as mean ± SEM ($n = 3$). P-values comparing observed values to expected values were calculated by Chi Square tests and adjusted for multiple comparisons,* $p < 0.05$, ** $P < 0.01$. P-values comparing observed values between stages were calculated using an unpaired 2-tailed, heteroscedastic t-test, * $p < 0.05$, ** $P < 0.01$, N.S. is non-significant. (E) H2Bub1 occupancy over H3K27me3 peaks in iPSCs (blue) and CMs (red). (F-G) Peak annotations (F) and Compartment information (G) for the H2Bub1 peaks on the same gene as H3K4me3. (H) Peak annotations for H2Bub1 peaks in the A compartment and in the B compartment. (I) Expression levels (from RNA-seq) for genes with H2Bub1 peaks in the A compartment and in the B compartment. 2-tailed, Mann-Whitney test, ** $p < 0.01$.

Development • Supplementary information

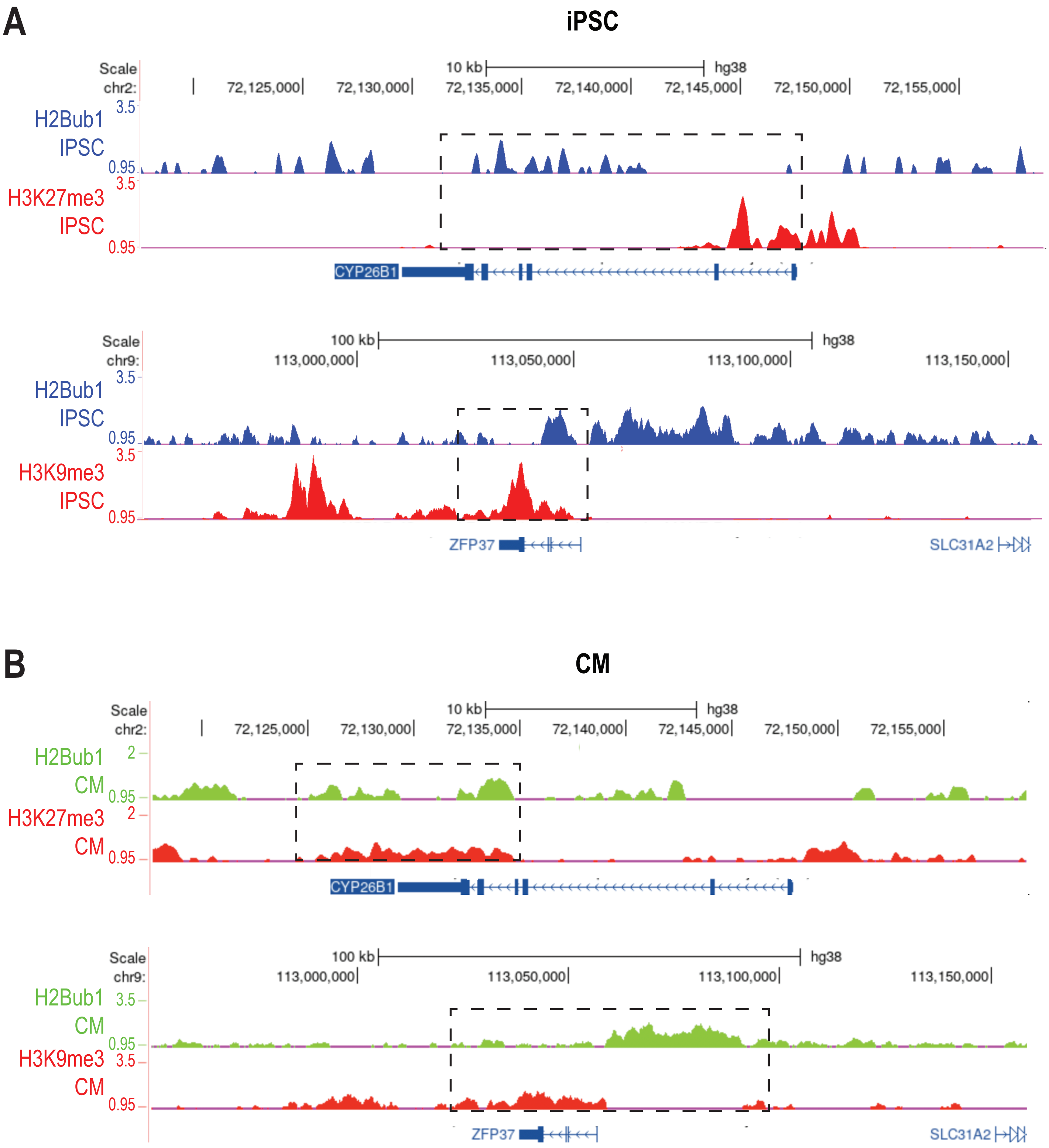

Fig. S7. Example genes illustration H2Bub1 overlap with repressive chromatin marks. Example H2Bub1 occupancy (iPSCs in blue in (A) and CMs in green in (B)), depicted using fold enrichment against random distribution, on the same gene as previously published H3K27me3 peaks (red) and previously published H3K9me3 peaks (red). Gene structure is indicated below the gene. The boxes highlight one gene that contains both H2Bub1 and H3K27me3 peaks and one gene that contains both H2Bub1 and H3K9me3 peaks.

Development • Supplementary information

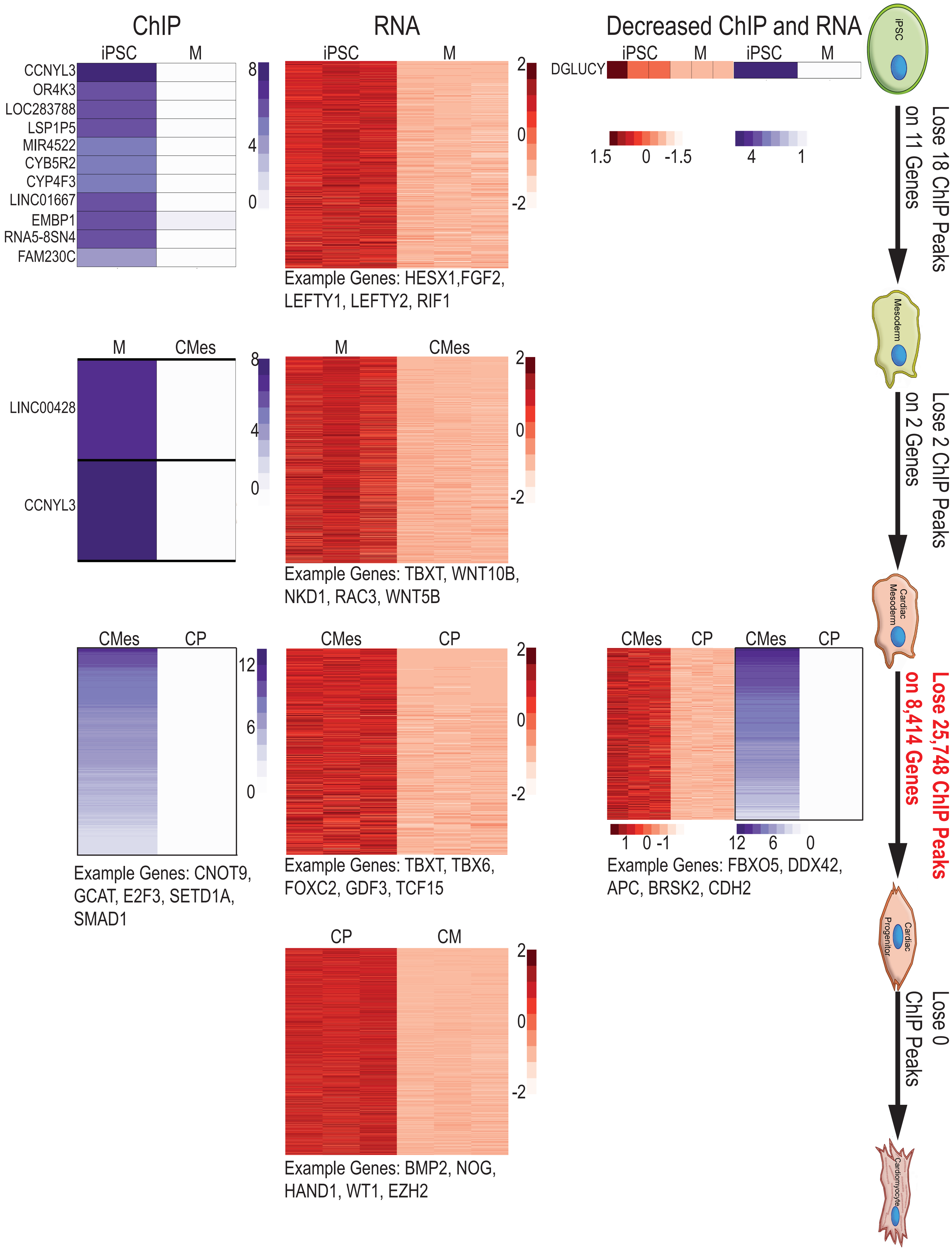
ChIP
RNA
Decreased ChIP and RNA
iPSC
M
CCNYL3
OR4K3
LOC283788
LSP1P5
MIR4522
CYB5R2
CYP4F3
LINC01667
EMBP1
RNA5-8SN4
FAM230C
8
4
0
2
0
-2
DGLUCY
1.5
0
-1.5
4
1
Example Genes: HESX1,FGF2, LEFTY1, LEFTY2, RIF1
Lose 18 ChIP Peaks on 11 Genes
M
CMes
LINC00428
CCNYL3
Example Genes: TBXT, WNT10B, NKD1, RAC3, WNT5B
Lose 2 ChIP Peaks on 2 Genes
CMes
CP
12
6
0
1
0
-1
Example Genes: CNOT9, GCAT, E2F3, SETD1A, SMAD1
Example Genes: TBXT, TBX6, FOXC2, GDF3, TCF15
Example Genes: FBXO5, DDX42, APC, BRSK2, CDH2
Lose 25,748 ChIP Peaks on 8,414 Genes
CP
CM
Example Genes: BMP2, NOG, HAND1, WT1, EZH2
Lose 0 ChIP Peaks
iPSC
Mesoderm
Cardiac Mesoderm
Cardiac Progenitor
Cardiomyocyte

Fig. S8. Genes with decreased H2Bub1 and RNA expression throughout cardiomyocyte differentiation. Heatmaps depicting the genes containing regions with decreased H2Bub1 occupancy, genes with decreased gene expression, and genes that have both decreased H2Bub1 occupancy and decreased gene expression when comparing iPSC and mesoderm (M), M and cardiac mesoderm (CMes), CMes and cardiac progenitor (CP), and CP and cardiomyocytes (CM). If there are less than 25 genes in a category, they are all listed. If there are more than 25 genes in a category, example genes are given below each heatmap. See supplemental data 1 and 2 for the complete list. The lost ChIP peaks were determined by a differential binding analysis for H2Bub1-ChIP-seq. There are 860 genes downregulated in M, 1364 genes downregulated in CMes, 1446 genes downregulated in CP, and 3141 genes downregulated in CM.

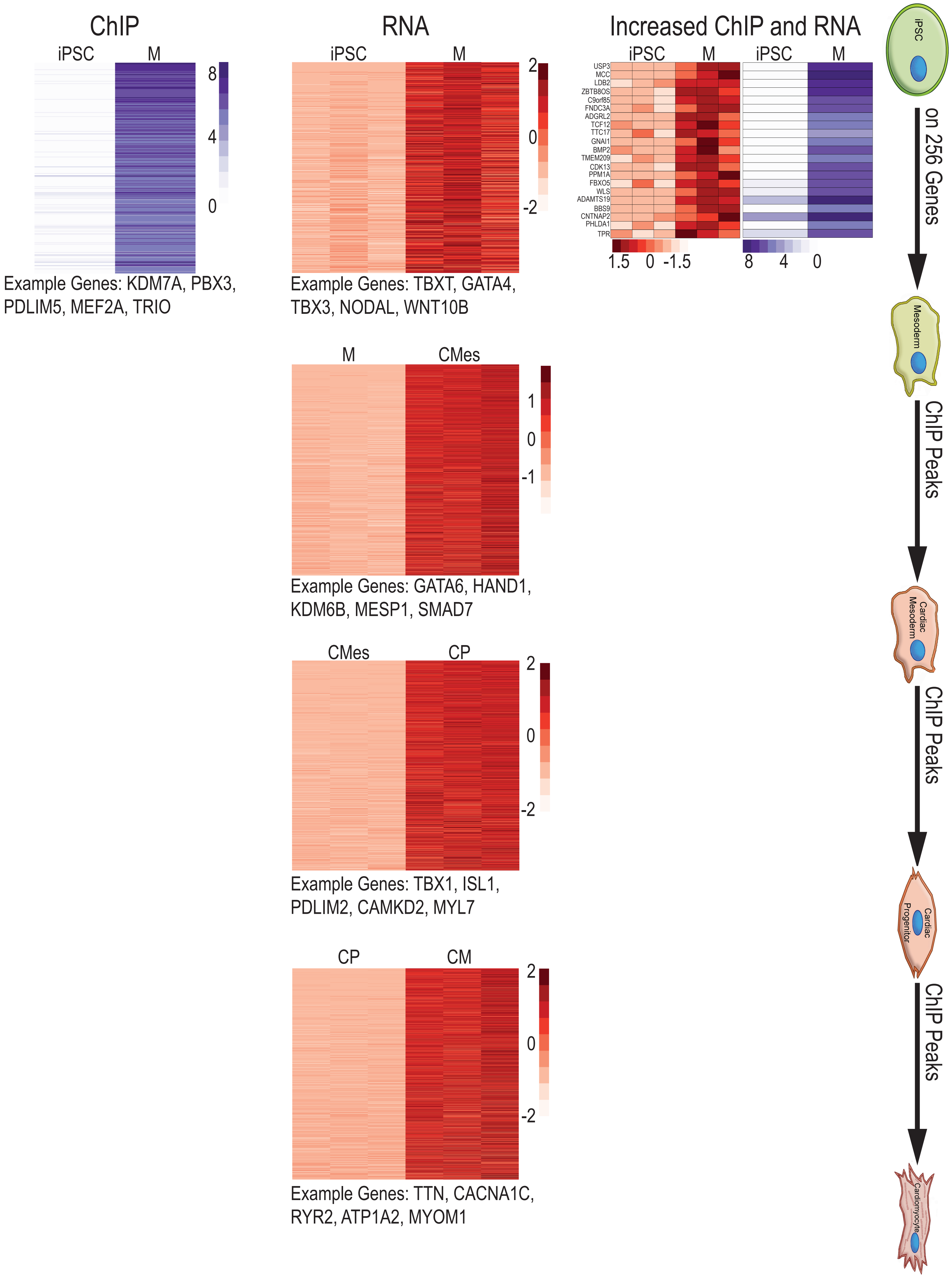

Development • Supplementary information

Fig. S9. Genes with increased H2Bub1 and RNA expression throughout cardiomyocyte differentiation. Heatmaps depicting the genes containing regions with increased H2Bub1 occupancy, genes with increased gene expression, and genes that have both increased H2Bub1 occupancy and increased gene expression when comparing iPSC and mesoderm (M), M and cardiac mesoderm (CMes), CMes and cardiac progenitor (CP), and CP and cardiomyocytes (CM). If there are less than 25 genes in a category, they are all listed. If there are more than 25 genes in a category, example genes are given below each heatmap. See supplemental data 1 and 2 for the complete list. The gained ChIP peaks were determined by a differential binding analysis for H2Bub1-ChIP-seq. There are 648 genes upregulated in M, 1844 genes upregulated in CMes, 1704 genes upregulated in CP, and 3490 genes upregulated in CM.

Development • Supplementary information

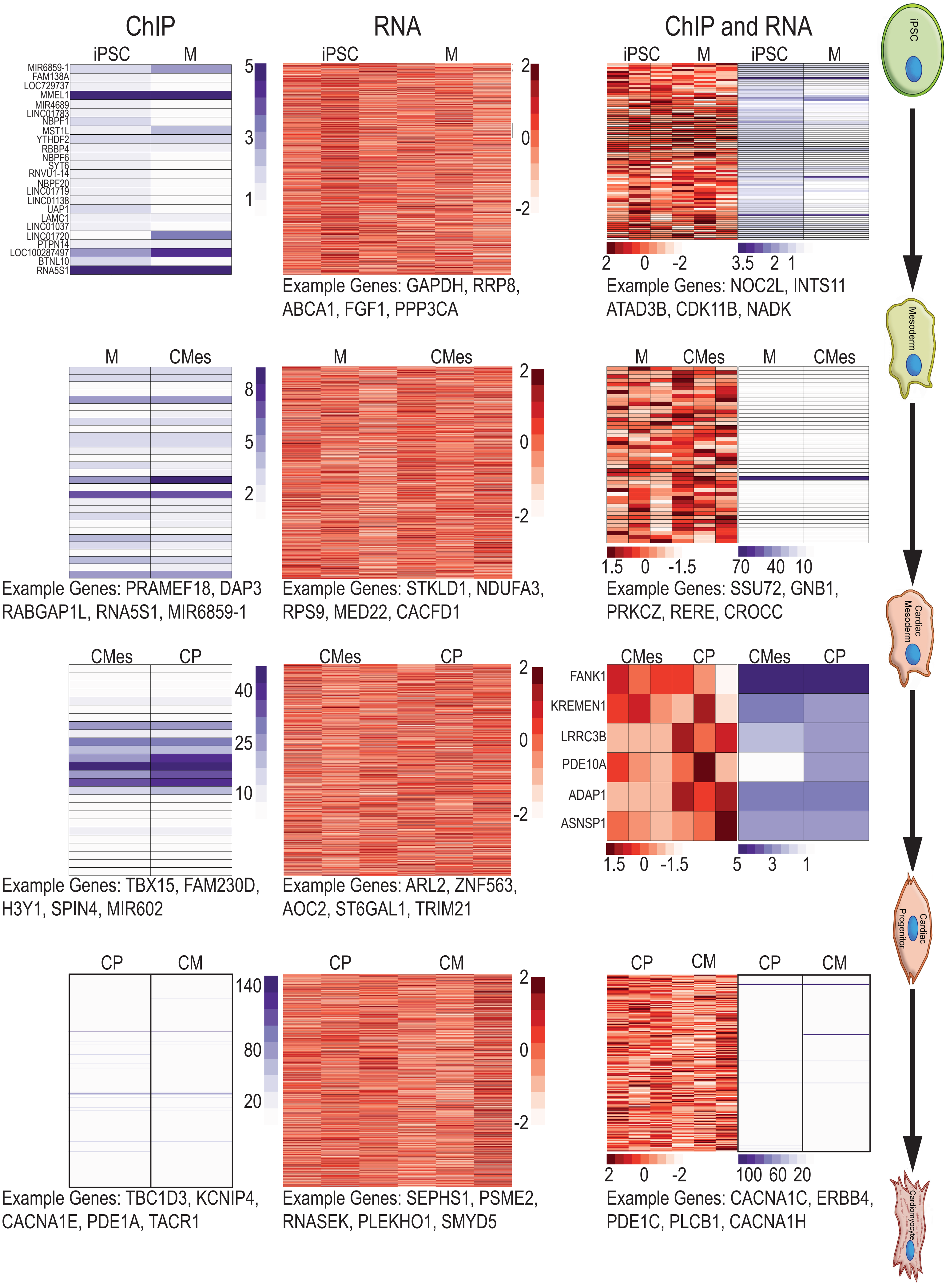

Development • Supplementary information

Fig. S10. Genes with constant H2Bub1 and RNA expression throughout cardiomyocyte differentiation. Heatmaps depicting the genes containing regions with constant H2Bub1 occupancy, genes with constant gene expression, and genes that have both constant H2Bub1 occupancy and constant gene expression when comparing iPSC and mesoderm (M), M and cardiac mesoderm (CMes), CMes and cardiac progenitor (CP), and CP and cardiomyocytes (CM). If there are less than 25 genes in a category, they are all listed. If there are more than 25 genes in a category, example genes are given below each heatmap. See supplemental data 1 and 2 for the complete list. The constant ChIP peaks were determined by a differential binding analysis for H2Bub1-ChIP-seq.

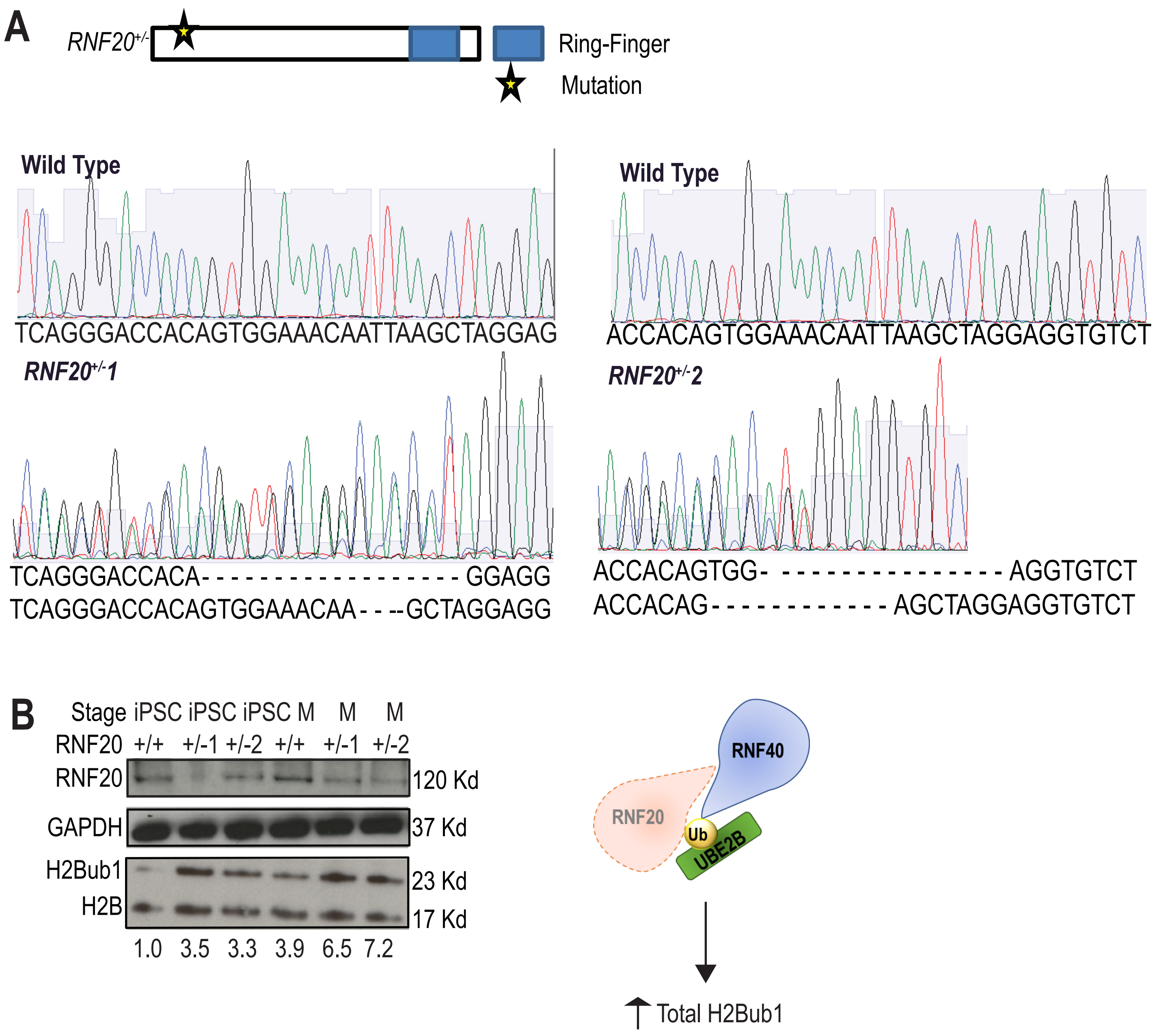

Fig. S11. Characteristics of the *RNF20*$^{+/-}$ mutants. (A) Diagram of the RNF20 protein with its domains (Ring-Finger domain is shown in blue) and where the created *RNF20* mutations are located (with a star). Lower four panels are sequencing traces of the wild-type and two independent *RNF20*$^{+/-}$ iPSC lines demonstrating CRISPR-generated mutations. (B) Western blot for RNF20 and H2Bub1 in two stages of CM differentiation (iPSC and Mesoderm (M)). The loading control for RNF20 is GAPDH and for H2Bub1 is H2B. Numbers indicate average quantification (imageJ) of H2Bub1 normalized to H2B over three replicates. Next to the western blot is a H2Bub1-deposition complex schematic illustrating the *RNF20*$^{+/-}$ iPSC mutant, which leads to increased total H2Bub1 levels.

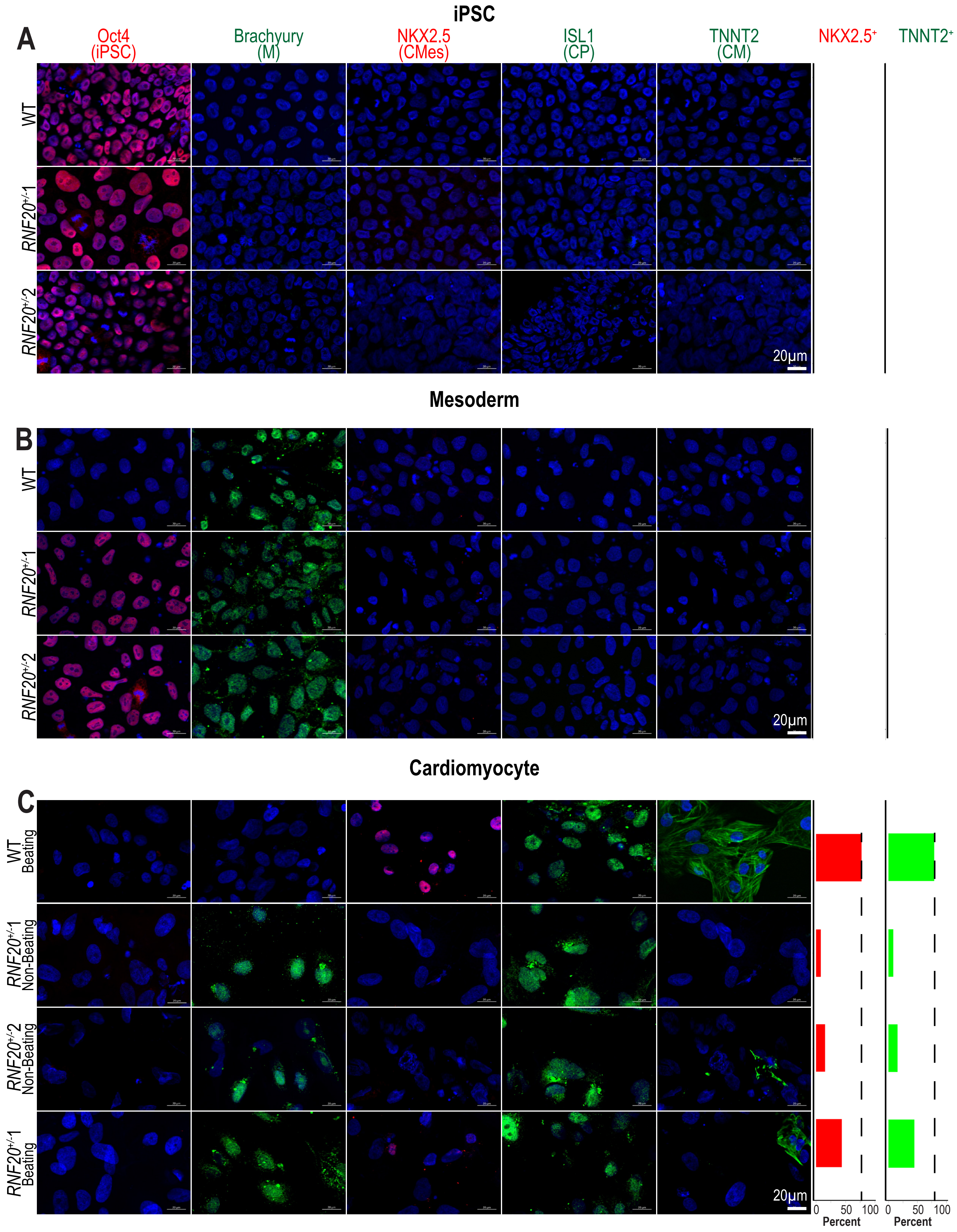

Development • Supplementary information

Fig. S12. Differentiation trajectory for *RN20*$^{+/-}$ mutants. Immunofluorescence staining for marker genes for each of five stages in CM differentiation (iPSC: OCT4 (red), mesoderm (M): Brachyury (green), cardiac mesoderm (CMes): NKX2.5 (red), cardiac progenitor (CP): ISL1 (green), and cardiomyocyte (CM): TNNT2 (green)) in the wild-type and both *RN20*$^{+/-}$ mutants at the iPSC stage (A), M stage (B) and CM stage (C). Since this immunofluorescence staining was done simultaneously with the immunofluorescence staining in Figure S14, the WT control images are reused in the figures. NKX2.5 and TNNT2 were assayed as a double labelling experiment. Since *RN20*$^{+/-}$*1* forms beating CMs some of the time, staining was done for beating and non-beating samples at the CM stage. Quantification of the percent of cells that are NKX2.5^{+} and TNNT2^{+} are displayed next to the images (WT n = 232, *RNF20*$^{+/-}$1 Non-beating n = 227, *RNF20*$^{+/-}$2 Non-beating n = 212, *RNF20*$^{+/-}$1 Beating n = 225).

Development • Supplementary information

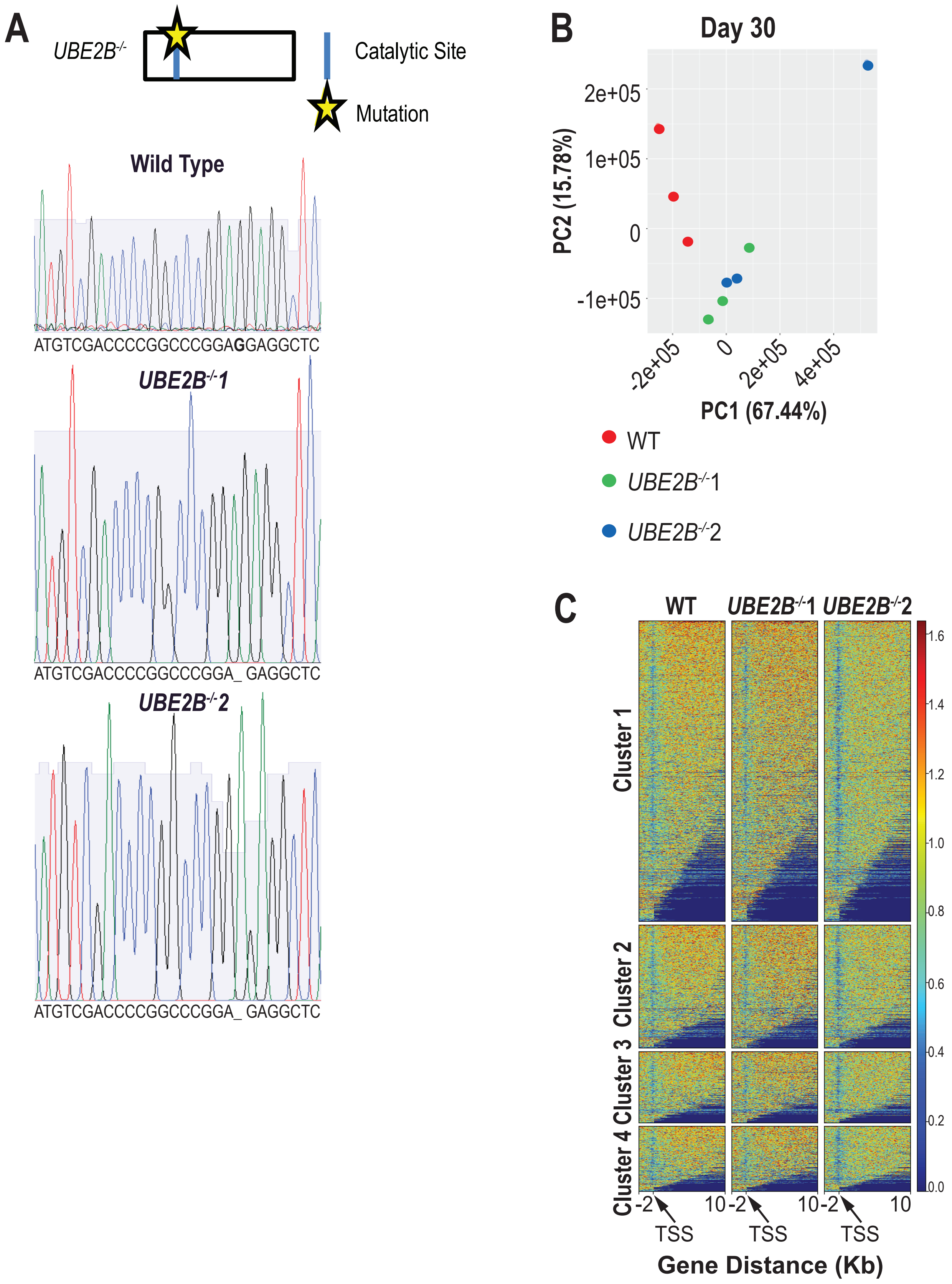

Fig. S13. Characteristics of the *UBE2B*$^{-/-}$ mutant. (A) Diagram of the UBE2B protein with its catalytic site, shown in blue, and where the created *UBE2B* mutations are located (with a star). Lower three panels are sequencing traces of the wild-type and two independent *UBE2B*$^{-/-}$ iPSC lines demonstrating CRISPR-generated mutations. (B) PCA plots of RNA-seq experiments comparing wild-type (red) and both *UBE2B*$^{-/-}$ mutants (green and blue) at the cardiomyocyte stage (n = 3). (C) H2Bub1 surrounding the transcriptional start site (TSS) (-2 Kb, +10 Kb) in the wild-type and *UBE2B*$^{-/-}$ mutants at the cardiomyocyte stage. Genes are grouped by H2Bub1 levels into four clusters. The average profile, depicted using fold enrichment against random distribution values, across this region for each cluster in each stage is shown as a heatmap. This represents the average of three replicates.

Development • Supplementary information

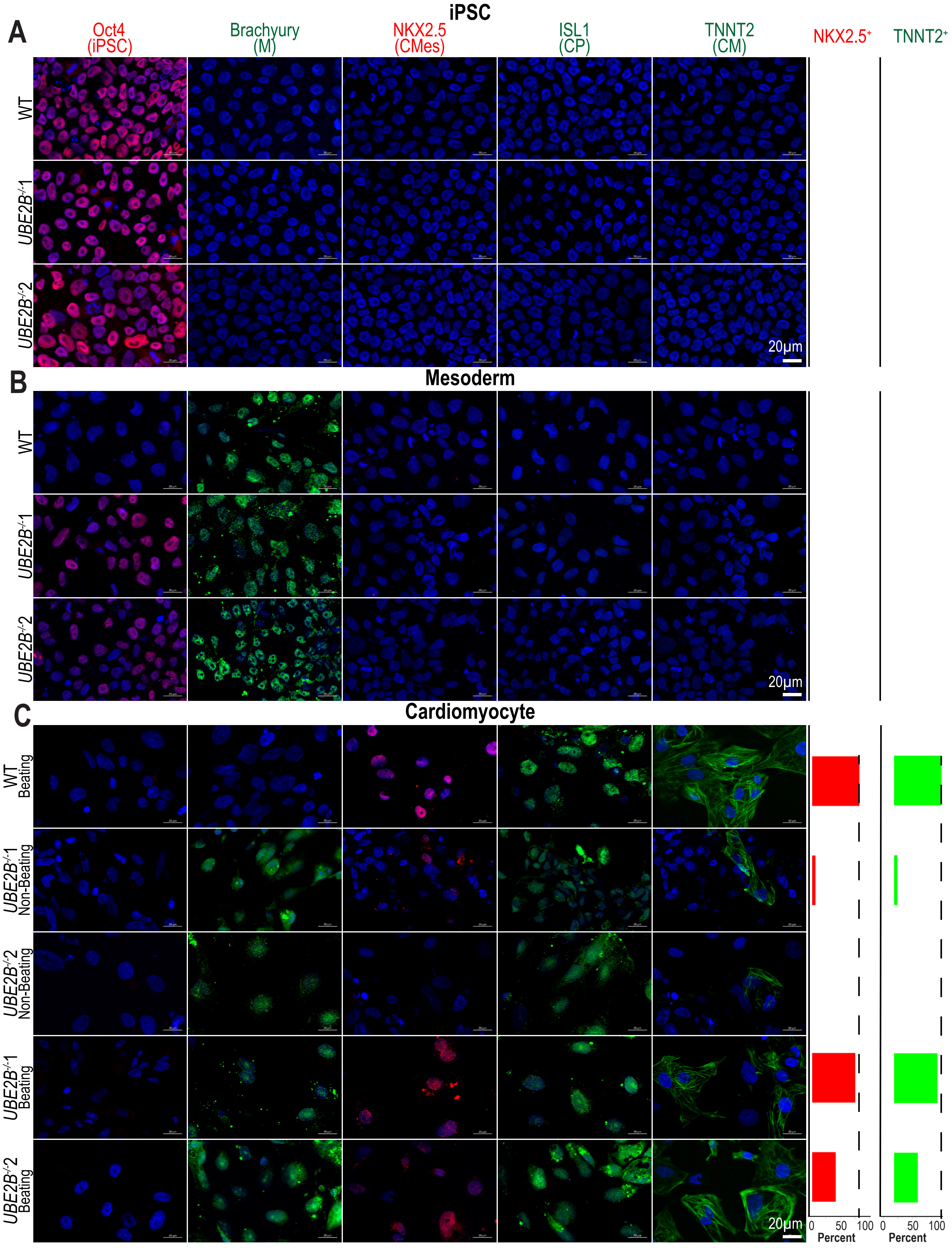
iPSC
A
Oct4
(iPSC)
Brachyury
(M)
NKX2.5
(CMes)
ISL1
(CP)
TNNT2
(CM)
NKX2.5+
TNNT2+
WT
UBE2B-/-1
UBE2B-/-2
20μm
B
Mesoderm
WT
UBE2B-/-1
UBE2B-/-2
20μm
C
Cardiomyocyte
WT Beating
UBE2B-/-1 Non-Beating
UBE2B-/-2 Non-Beating
UBE2B-/-1 Beating
UBE2B-/-2 Beating
20μm
0 50 100
Percent
0 50 100
Percent

Fig. S14. Differentiation trajectory for *UBE2B$^{-/-}$* mutants. Immunofluorescence staining for marker genes for each of five stages in CM differentiation (iPSC: OCT4 (red), mesoderm (M): Brachyury (green), cardiac mesoderm (CMes): NKX2.5 (red), cardiac progenitor (CP): ISL1 (green), and cardiomyocyte (CM): TNNT2 (green)) in the wild-type and both *UBE2B$^{-/-}$* mutants at the iPSC stage (A), M stage (B), and CM stage (C). Since this immunofluorescence staining was done simultaneously with the immunofluorescence staining in Figure S12, the WT control images are reused in the figures. NKX2.5 and TNNT2 were assayed as a double labelling experiment. Since both mutants form beating CMs some of the time, staining was done for beating and non-beating samples at the CM stage. Quantification of the percent of cells that are NKX2.5^{+} and TNNT2^{+} are displayed next to the images (WT n = 232, *UBE2B$^{-/-}$*1 Non-beating n = 231, *UBE2B$^{-/-}$*2 Non-beating n = 232, *UBE2B$^{-/-}$*1 Beating n = 231, *UBE2B$^{-/-}$*2 Beating n = 230).

Development • Supplementary information

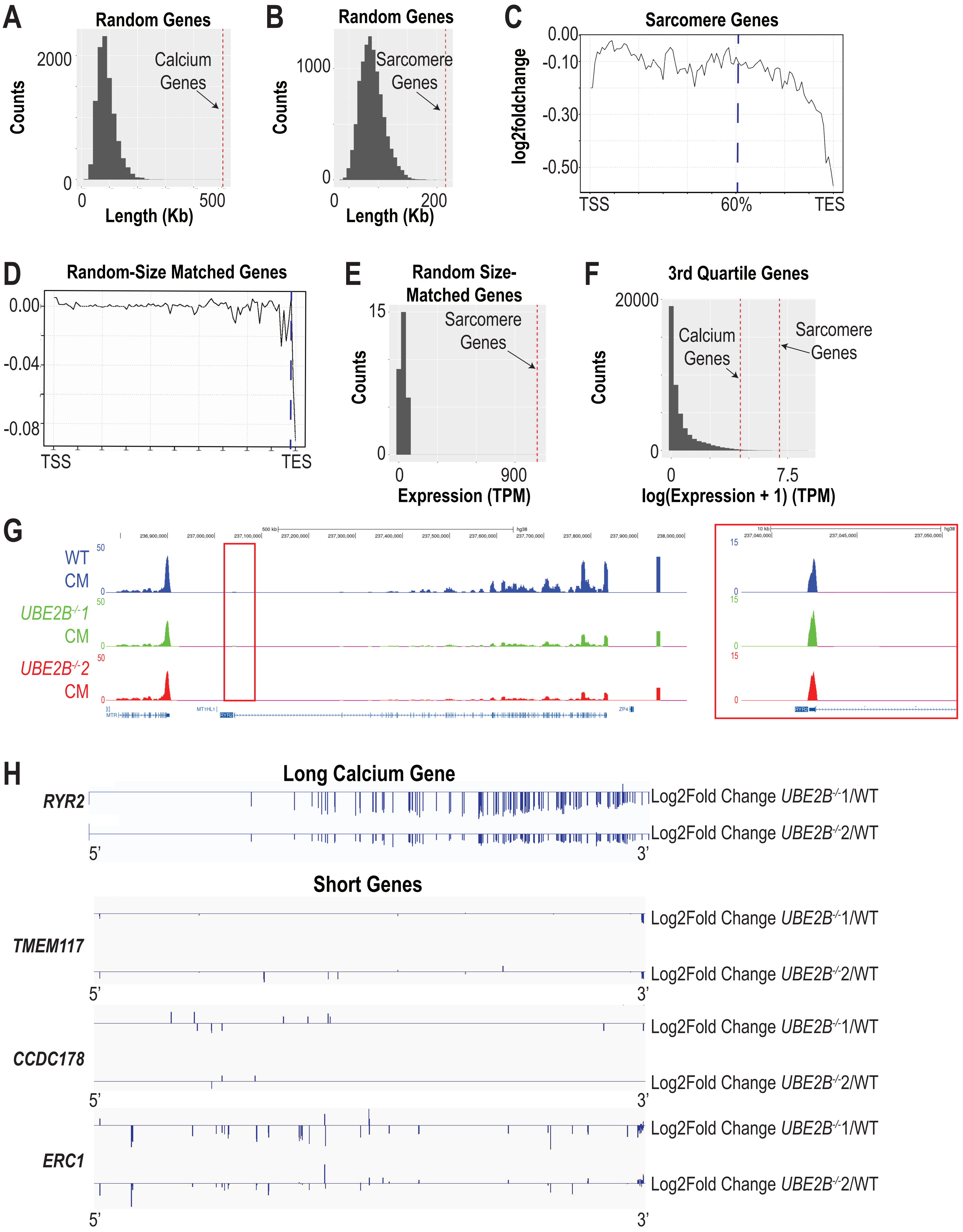

Development • Supplementary information

Fig. S15. Amount of full-length transcript controls. (A-B) Histograms showing the average length in Kb of each of 10,000 random quantity matched gene sets to the calcium and sarcomere gene sets. The vertical dashed red lines show the average length in Kb of the calcium genes (A) and the sarcomere genes (B). (C-D) Log2 of fold change in transcript abundance between wild-type and *UBE2B*$^{-/-}$ mutants is shown at each position. The genes shown in (C) are the sarcomere genes (n = 70 genes) that are differentially expressed between wild-type and *UBE2B*$^{-/-}$ mutants (n = 3, for each of 2 cell lines). The genes shown in (D) are "randomly" selected quantity and size-matched genes to the sarcomere gene set (n = 3, for each of 2 cell lines). 30 "random" plots were created from the sarcomere gene set: 10 from genes that are upregulated between wild-type and both *UBE2B*$^{-/-}$ cell lines, 10 that are non-regulated between wild-type and both *UBE2B*$^{-/-}$ cell lines, and 10 that are down-regulated between wild-type and both *UBE2B*$^{-/-}$ cell lines. (E) A histogram showing the average expression in TPM of each of the "random" quantity and size-matched gene sets described in (D). The vertical dashed red line shows the average expression in TPM of the sarcomere genes. (F) A histogram showing the log average expression in TPM of all of the 3rd Quartile genes (greater than 33.940 Kb and less than 93.323 Kb). The vertical dashed red lines show the average expression in TPM of the calcium genes (left) and sarcomere genes (right). (G) Expression of an example calcium gene, *RYR2*, shown in Figure 5D. 5' on the left of the diagram (WT (blue), *UBE2B*$^{-/-}$*1* (green), and *UBE2B*$^{-/-}$*2* (red)). Zoom in of exon 1 (red box region) is shown on the right. (H) Example log2 of fold change gene traces for one calcium gene (RYR2) and non-calcium short genes (*TMEM117, CCDC178* and *ERC1*). The short genes do not have accumulation of H2Bub1 near the center of the gene and do not have decreased full-length transcripts. 5' on the left of the diagram.

Development • Supplementary information

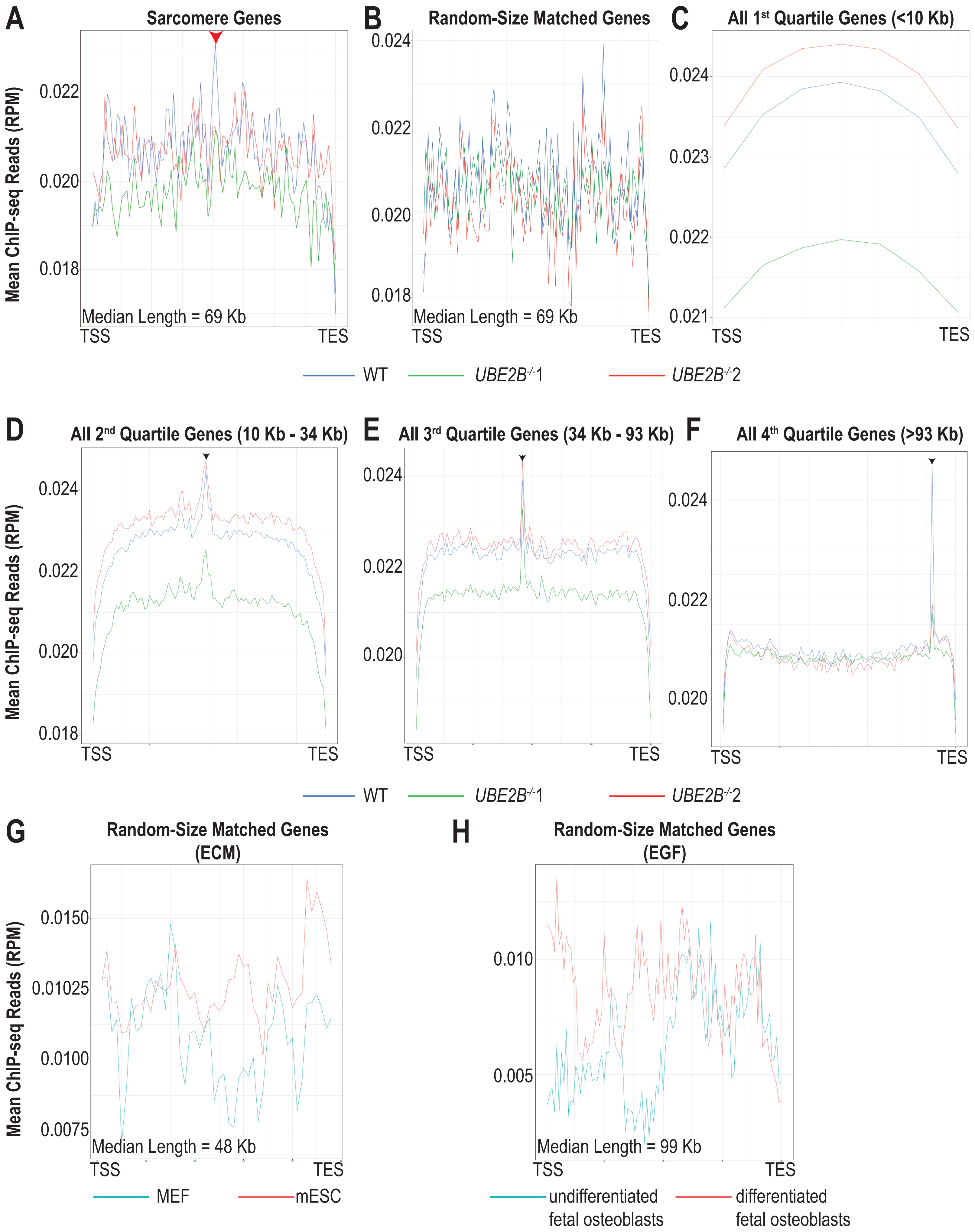

Development • Supplementary information

Fig. S16. Metagene controls. (A-F) Metagene plots for H2Bub1 levels in wild-type (blue) and *UBE2B*$^{-/-}$ mutants (green and red) (n = 3). The arrows indicate the accumulation near the center of the gene. The red arrow indicates UBE2B-dependent accumulation, while the black arrow indicates UBE2B-independent accumulation. The genes shown in (A) are the sarcomere genes described in Figure S15C and the genes shown in (B) are an example of a “random” quantity and size-matched gene set described in Figure S15D. The 1st quartile genes (less than 9.784 Kb) are shown in (C), the 2nd quartile genes (greater than 9.784 Kb and less than 33.940 Kb) are shown in (D), the 3rd quartile genes (greater than 33.940 Kb and less than 93.323 Kb) are shown in (E), and the 4th quartile genes (greater than 93.323 Kb) are shown in (F). (G) Example metagene plot for H2Bub1 levels in MEFs (teal) and mESCs (red) across “randomly” selected quantity and size-matched genes to the ECM gene set. 20 “random” metagene plots were created from the ECM gene set. (H) Example metagene plot for H2Bub1 levels in undifferentiated hFOBs (teal) and differentiated hFOBs (red) across “randomly” selected quantity and size-matched genes to the EGF related gene set. 20 “random” metagene plots were created from the EGF related gene set.

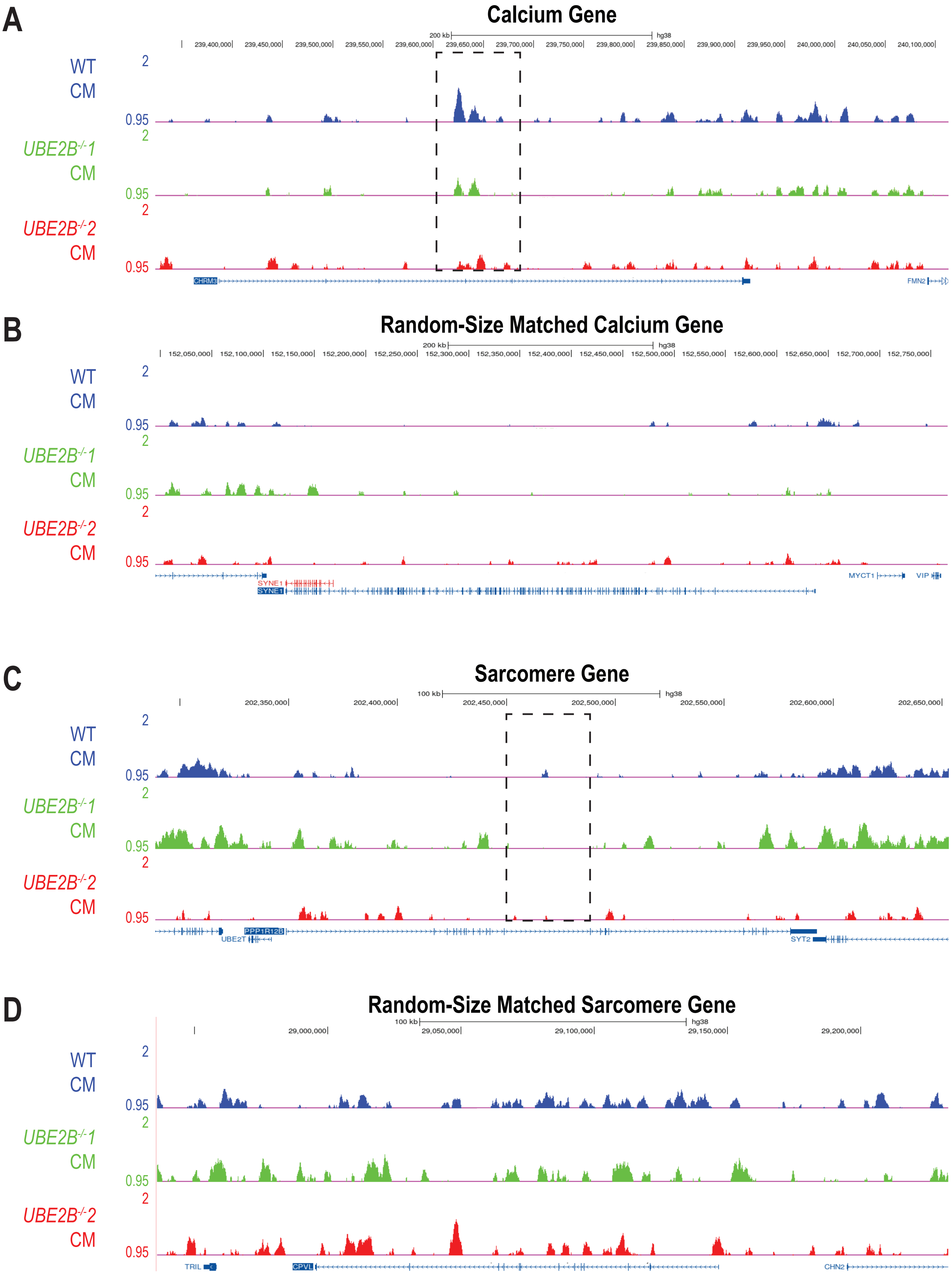

Fig. S17. Metagene examples. Example long tissue-specific genes (A and C) with H2Bub1 accumulation near the center of the gene and example random-size matched genes (B and D) without H2Bub1 accumulation near the center of the gene. 5' on the left of the diagram (WT (blue), *UBE2B*$^{-/-}$*1* (green), and *UBE2B*$^{-/-}$*2* (red)). The boxes highlight the accumulation regions.

Development • Supplementary information

Table S1. Primers A list of all of the primers used in this study and their application.

Primer Name	Sequence	Species	Application
18s F	CTTAGAGGGACAAGTGGCG	Mouse	Calcium qRT-PCR Experiment
18s R	ACGCTGAGCCAGTCAGTGTA	Mouse	Calcium qRT-PCR Experiment
*Cacna1c*F (Gomez-Ospina et al., 2013)	CCTAATGGGTTCGTTTCAGAAGT	Mouse	Calcium qRT-PCR Experiment
*Cacna1c*R (Gomez-Ospina et al., 2013)	TCCGGTTACCTCCAGGTCA	Mouse	Calcium qRT-PCR Experiment
Ncx F (Santulli et al., 2015)	AGATCAAGCATCTGCGTGTG	Mouse	Calcium qRT-PCR Experiment
Ncx R (Santulli et al., 2015)	CTCCACAACTCCAGGAGAGC	Mouse	Calcium qRT-PCR Experiment
Ryr2 F (Santulli et al., 2015)	CCTTGCCTGAGTGCAGTTG	Mouse	Calcium qRT-PCR Experiment
Ryr2 R (Santulli et al., 2015)	TTGAGGTATCAACAGGTTGTGG	Mouse	Calcium qRT-PCR Experiment
Serca2a F (Chung et al., 2019)	GGGCAAAGTGTATCGACAGG	Mouse	Calcium qRT-PCR Experiment
Serca2a R (Chung et al., 2019)	TCAGCAGGAACTTTGTCACC	Mouse	Calcium qRT-PCR Experiment
GAPDH Exon F	GCAGGCCGGATGTGTTC	Human	Control for H2Bub1 ChIP-seq
GAPDH Exon R	AGAACAGTGAGCGCCTAGT	Human	Control for H2Bub1 ChIP-seq
GAPDH Promoter F	GGGCTCTCCAGAACATCATC	Human	Control for H2Bub1 ChIP-seq
GAPDH Promoter R	CAGTGAGCTTCCCGTTCAG	Human	Control for H2Bub1 ChIP-seq
RNF20 F	ACGACTGTCTTCTTCTGCCA	Human	Genotyping iPSC Mutants
RNF20 R	ACCACCTTCCCAGATTCTCG	Human	Genotyping iPSC Mutants
RNF20 Sequence	GCTTCCCCATACTCCAGAAA	Human	Genotyping iPSC Mutants
UBE2B F	ACGTCATTGCAGGGTTGTTT	Human	Genotyping iPSC Mutants
UBE2B R	CGGCAAAGCTTATGGGAGTA	Human	Genotyping iPSC Mutants
UBE2B Sequence	GGCCCTTACCGCTTGAAATC	Human	Genotyping iPSC Mutants
Rnf20$^{+/-}$ F Null allele	CCATTACCAGTTGGTCTGGTGTC	Mouse	Mouse genotyping
Rnf20$^{+/-}$ F WT allele	GACCTTCACCTCAAGTCTAGCAGAG	Mouse	Mouse genotyping
Rnf20$^{+/-}$ R Null allele	TCCTCACTATGTTCTCTCGCTACTG	Mouse	Mouse genotyping
Rnf20$^{+/-}$ R WT allele	TCCTCACTATGTTCTCTCGCTACTG	Mouse	Mouse genotyping
UBE2B F	CAGCTGCGGAGCATGTCG	Human	Sequencing cDNA
UBE2B R	CAACAATGGCCGAAACTCTT	Human	Sequencing cDNA

Development • Supplementary information

Table S2. Antibodies A list of all of the antibodies used in this study, their species, their dilution, and their application.

Antibody	Catalogue Number	Species	Dillution	Application
H2Bub1	Cell Signaling 5546s	Rabbit	4 μL	ChIP
Igg	Cell Signaling 2729s	Rabbit	4 μL	ChIP
Brachyury	R&D systems AF2085-SP	Goat	1:20	IF cells
Cardiac Troponin T	Invitrogen MA5-12960	Mouse	1:200	IF cells
ISL1	DSHB 39.4D5 supernatant	Rat	1:10	IF cells
NKX2.5	Santa Cruz sc-8697	Goat	1:200	IF cells
OCT-4a	Cell Signaling 2840S	Rabbit	1:200	IF cells
CD31	BD biosciences 550274	Rat	1:500	IF sections
NKX2.5	Santa Cruz sc-8697	Goat	1:100	IF sections
RNF20	Cell Signaling 11974	Rabbit	1:200	IF sections
WT1	Novus 6f-h2	Mouse	1:250	IF sections
alpha-tubulin	Invitrogen 62204	Mouse	1:1000	Western
GAPDH	Invitrogen MA515738HRP	Mouse	1:20,000	Western
H2B	Millipore 07-371	Rabbit	1:12,000	Western
H2Bub1	Cell Signaling 5546s	Rabbit	1:200,000	Western
RNF20	Cell Signaling 11974	Rabbit	1:500	Western
RNF40	Abcam ab191309	Rabbit	1:1000	Western
UBE2B	GenTex GTX100416	Rabbit	1:500	Western
A488 anti-Goat	Invitrogen A21467	Chicken	1:500	IF (cells)
A594 anti-Goat	Invitrogen A21468	Chicken	1:500	IF (section, cells)
A594 anti-Rabbit	Invitrogen A32754	Donkey	1:500	IF (section, cells)
A488 anti-Rabbit	Invitrogen A21206	Donkey	1:500	IF (section)
A647 anti-mouse	Invitrogen A31571	Donkey	1:500	IF (section)
A488 anti-Mouse	Invitrogen A21200	Chicken	1:500	IF (section, cells)
A488 anti-Rat	Invitrogen A21470	Chicken	1:500	IF (section, cells)
A647 anti-Rat	Invitrogen A21472	Chicken	1:500	IF (section)

Table S3. Guide RNAs A list of all of the guide RNAs used for CRISPR in iPSCs.

Guide	Sequence	Cell Line(s) Developed
RNF20_1	ACAGTGGAAACAATTAAGCT	*RNF20$^{+/-}$1* and *RNF20$^{+/-}$2*
RNF20_2	GTGGAAACAATTAAGCTAGG	N/A
UBE2B_1	AATCCCGCATGAGCCTCCTC	*UBE2B$^{-/-}$1*
UBE2B_2	ATCCCGCATGAGCCTCCTCC	*UBE2B$^{-/-}$2*

Table S4. Encode Accession Numbers A list of all of the Encode Data that was used in this study.

Experiment	Bedfile	Target	Tissue
ENCSR864LRY	ENCFF289NJD	H3K27me3	cardiac muscle originated from RUES2
ENCSR000ALU	ENCFF296RYM	H3K27me3	Human h1-hESC
ENCSR652QNW	ENCFF905NTT	H3K4me3	cardiac muscle originated from RUES2
ENCSR000AMG	ENCFF668YOE	H3K4me3	Human h1-hESC
ENCSR000APZ	ENCFF654ZZO	H3K9me3	Human h1-hESC
ENCSR269ULZ	ENCFF677NKI	H3K9me3	cardiac muscle originated from RUES2

Table S5. ChIP-seq wild-type iPSC-derived cardiomyocytes

Tab 1: contains the genes in Clusters 1-4 displayed in Fig. 3a.

Tabs 2-21: contain the gene ontology terms for the genes in Clusters 1-4 in iPSC, mesoderm (M), cardiac mesoderm (CMes), cardiac progenitor (CP), and cardiomyocyte (CM) in Fig. 3a.

Tabs 22-29: contain the differentially occupied regions between all of the consecutive stages (iPSC v. M, M v. CMes, CMes v. CP, and CP v. CM) of CM differentiation (Fig. 3, S8, S9).

Available for download at
https://journals.biologists.com/dev/article-lookup/doi/10.1242/dev.201899#supplementary-data

Table S6. RNA-seq wild-type iPSC-derived cardiomyocytes

Tabs 1-8: contain the genes that are differentially expressed between all of the consecutive stages (iPSC v. mesoderm (M), M v. cardiac mesoderm (CMes), CMes v. cardiac progenitor (CP), and CP v. cardiomyocyte (CM)) of CM differentiation (Fig. S8, S9).

Tab 9: contains the RNA-seq quality metrics.

Available for download at
https://journals.biologists.com/dev/article-lookup/doi/10.1242/dev.201899#supplementary-data

Table S7. ChIP-seq *UBE2B*$^{-/-}$ cardiomyocytes

Tab 1: contains the genes in Clusters 1-4 displayed in Fig. S13c.

Tabs 2-5: contain the differentially occupied regions between wild-type and *UBE2B*$^{-/-}$ cardiomyocytes (Fig. S16).

Tabs 6-9: contain the genes used for each metagene plot (Fig. 5, S16).

Available for download at
https://journals.biologists.com/dev/article-lookup/doi/10.1242/dev.201899#supplementary-data

Table S8. RNA-seq *UBE2B*$^{-/-}$ cardiomyocytes

Tabs 1-4: contain the genes that are differentially expressed between wild-type and *UBE2B*$^{-/-}$ cardiomyocytes (Fig. 4c, S13).

Tab 5: contains the RNA-seq quality metrics.

Available for download at
https://journals.biologists.com/dev/article-lookup/doi/10.1242/dev.201899#supplementary-data

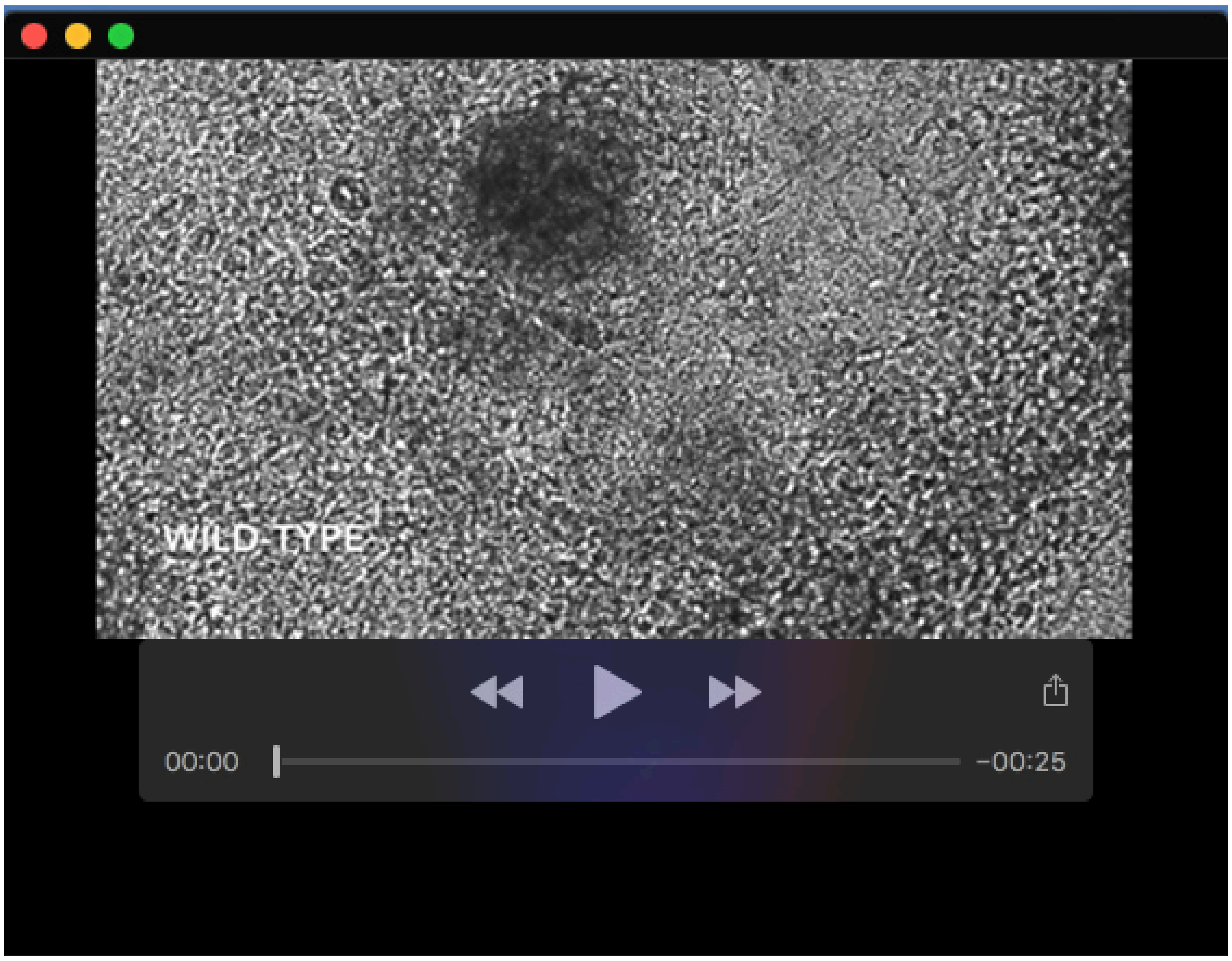

Movie 1. Movie shows wild-type cardiomyocytes under bright-field microscopy.

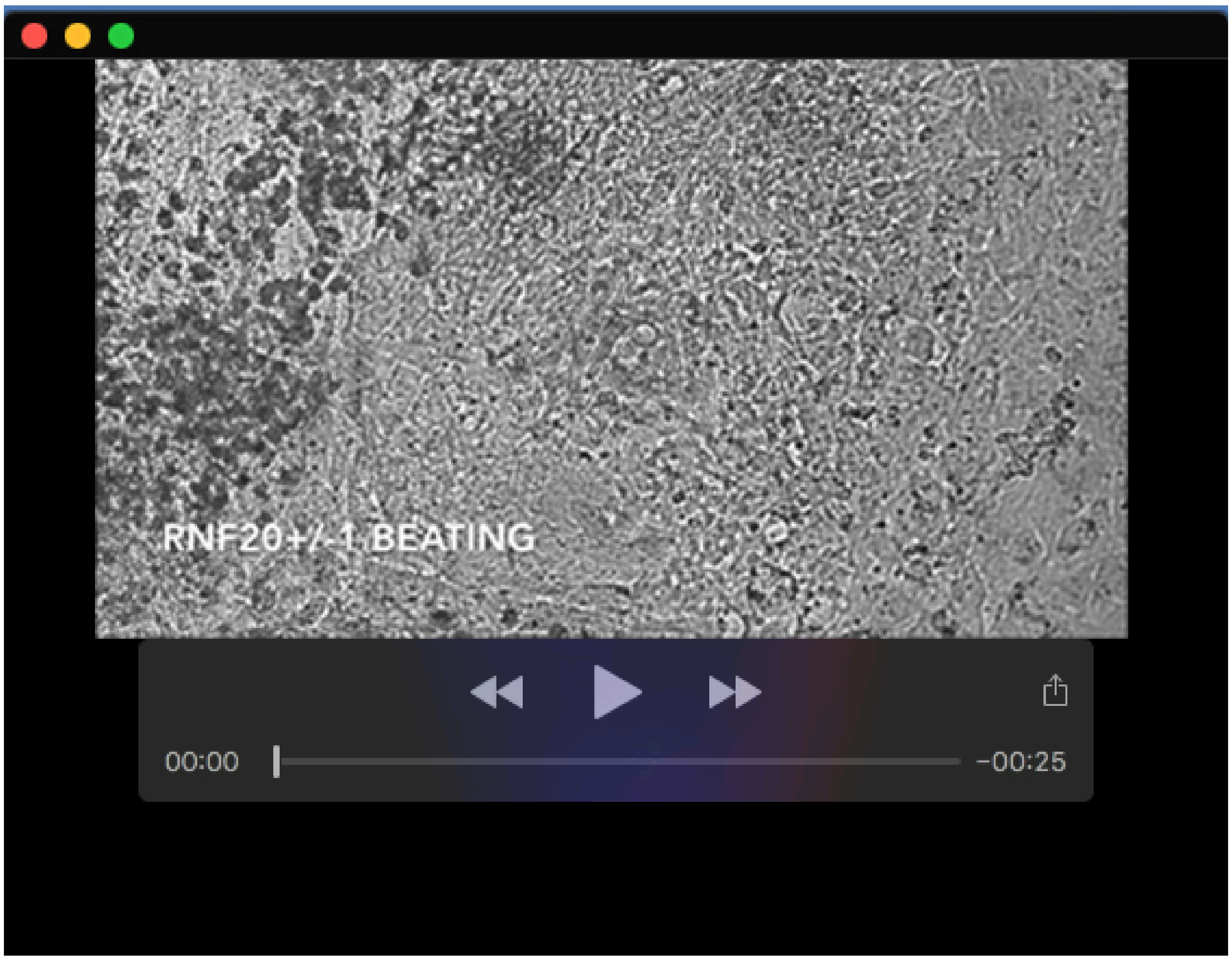

Movie 2. Movie shows *RNF20*$^{+/-}$*1* cardiomyocytes beating under bright-field microscopy.

Development • Supplementary information

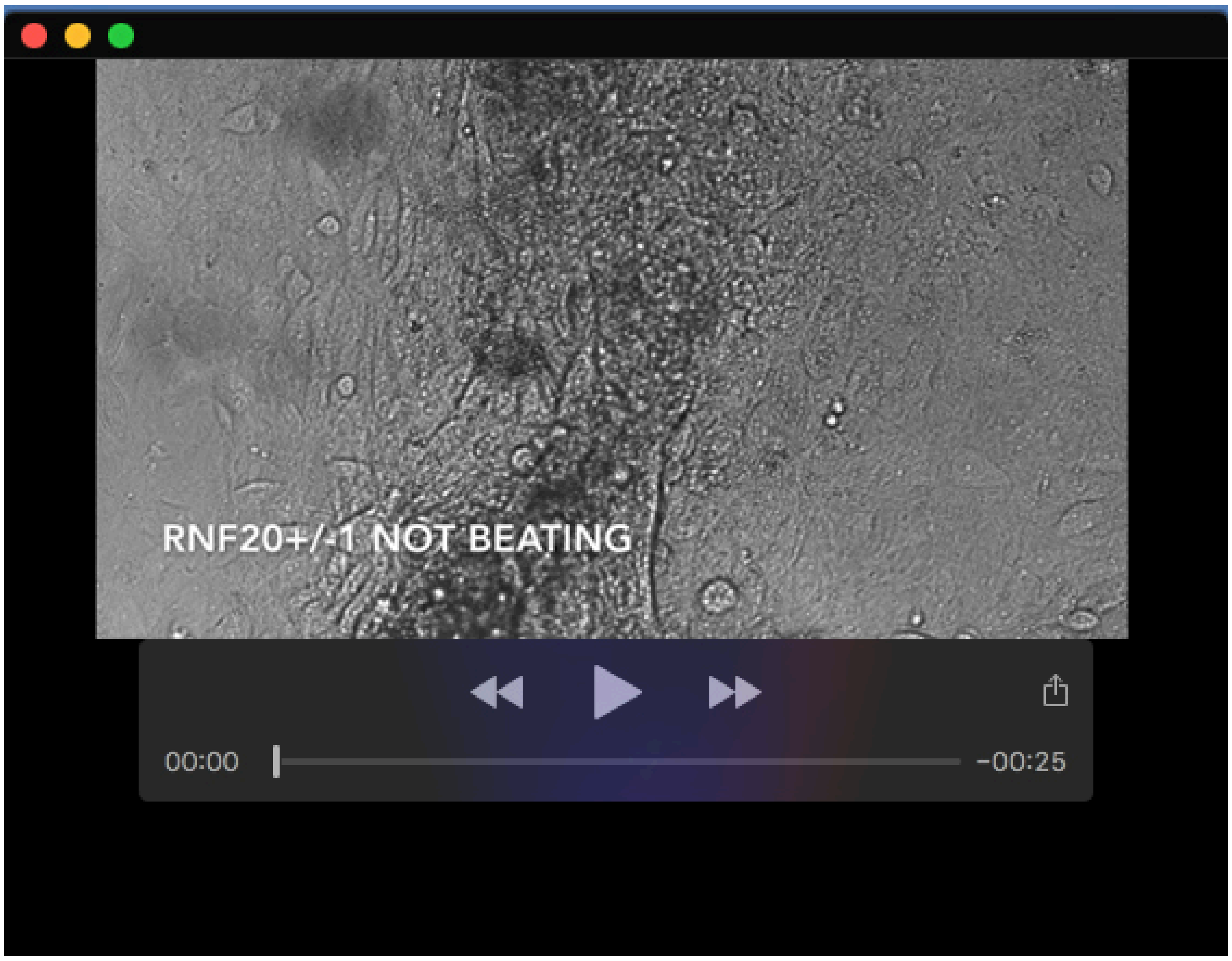

Movie 3. Movie shows *RNF20*$^{+/-}$*1* cardiomyocytes not beating under bright-field microscopy.

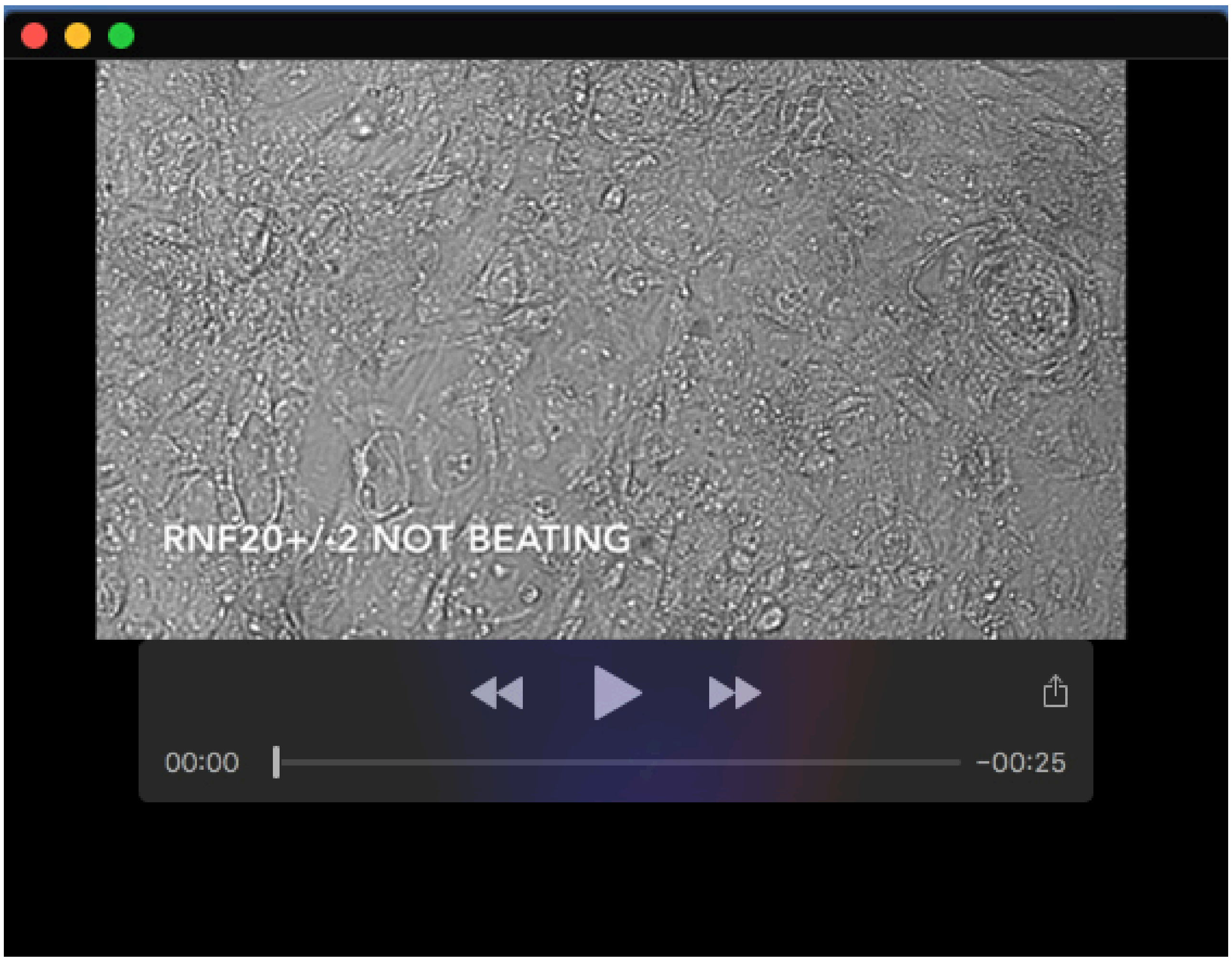

Movie 4. Movie shows *RNF20*$^{+/-}$*2* cardiomyocytes not beating under bright-field microscopy.

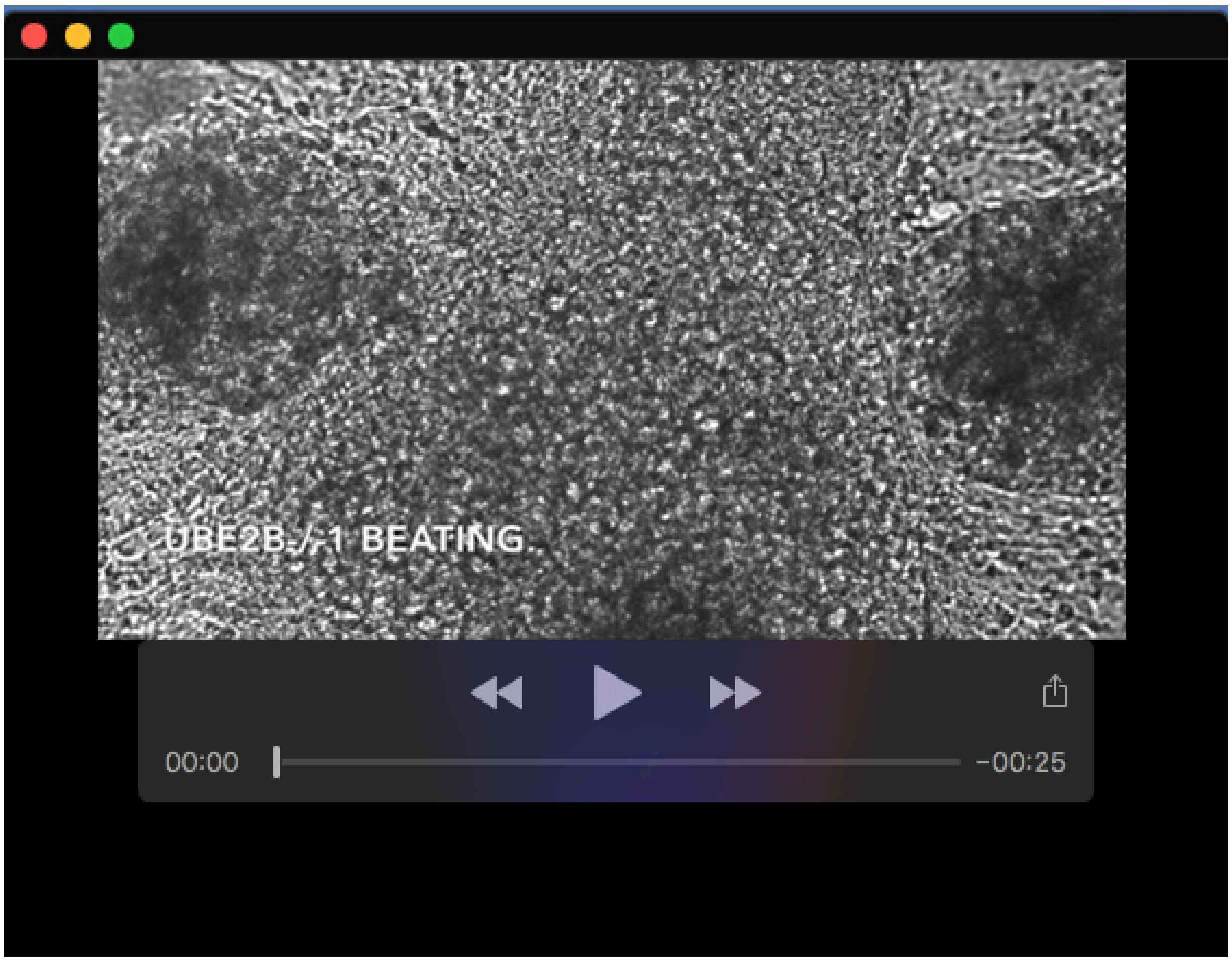

Movie 5. Movie shows *UBE2B*$^{-/-}$*1* cardiomyocytes beating under bright-field microscopy.

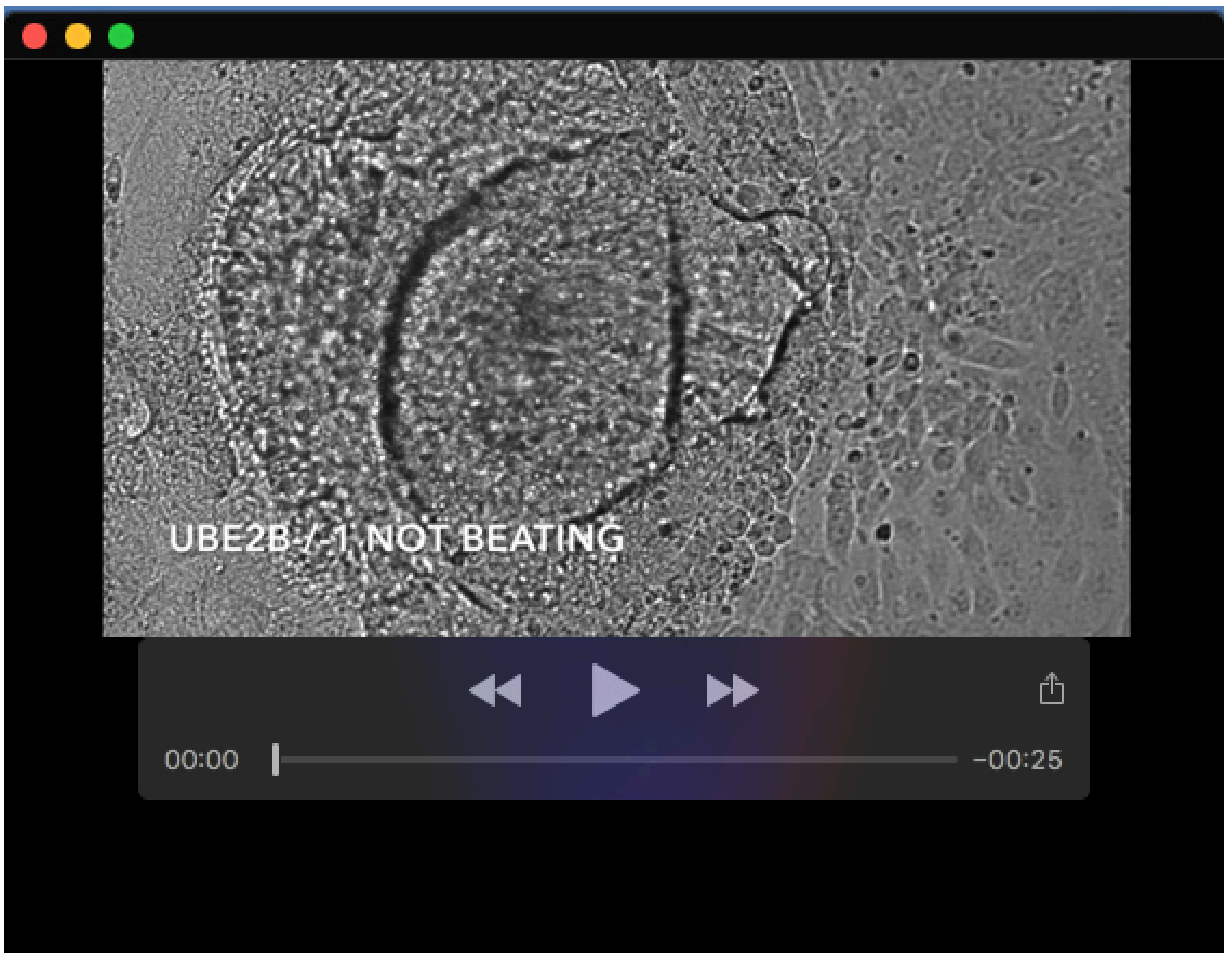

Movie 6. Movie shows *UBE2B*$^{-/-}$*1* cardiomyocytes not beating under bright-field microscopy.

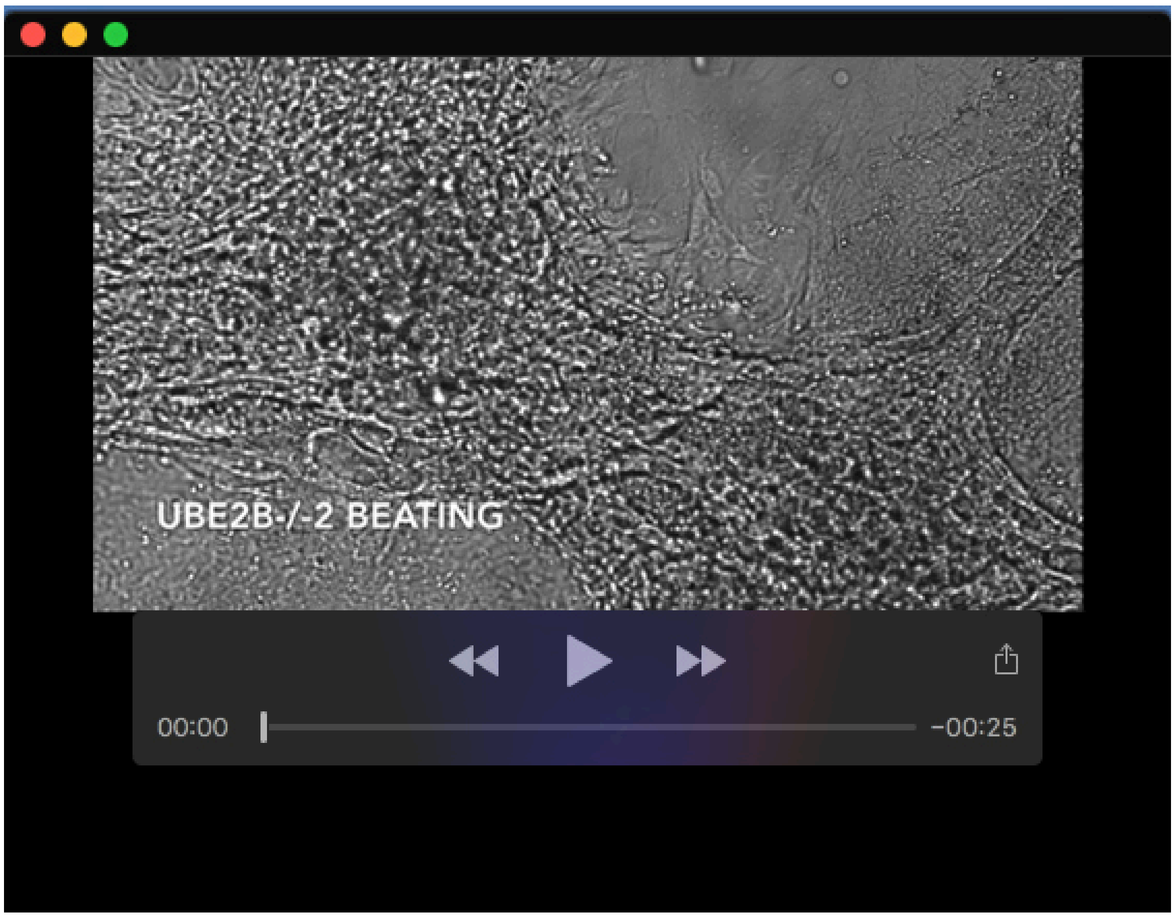

Movie 7. Movie shows *UBE2B*$^{-/-}$*2* cardiomyocytes beating under bright-field microscopy.

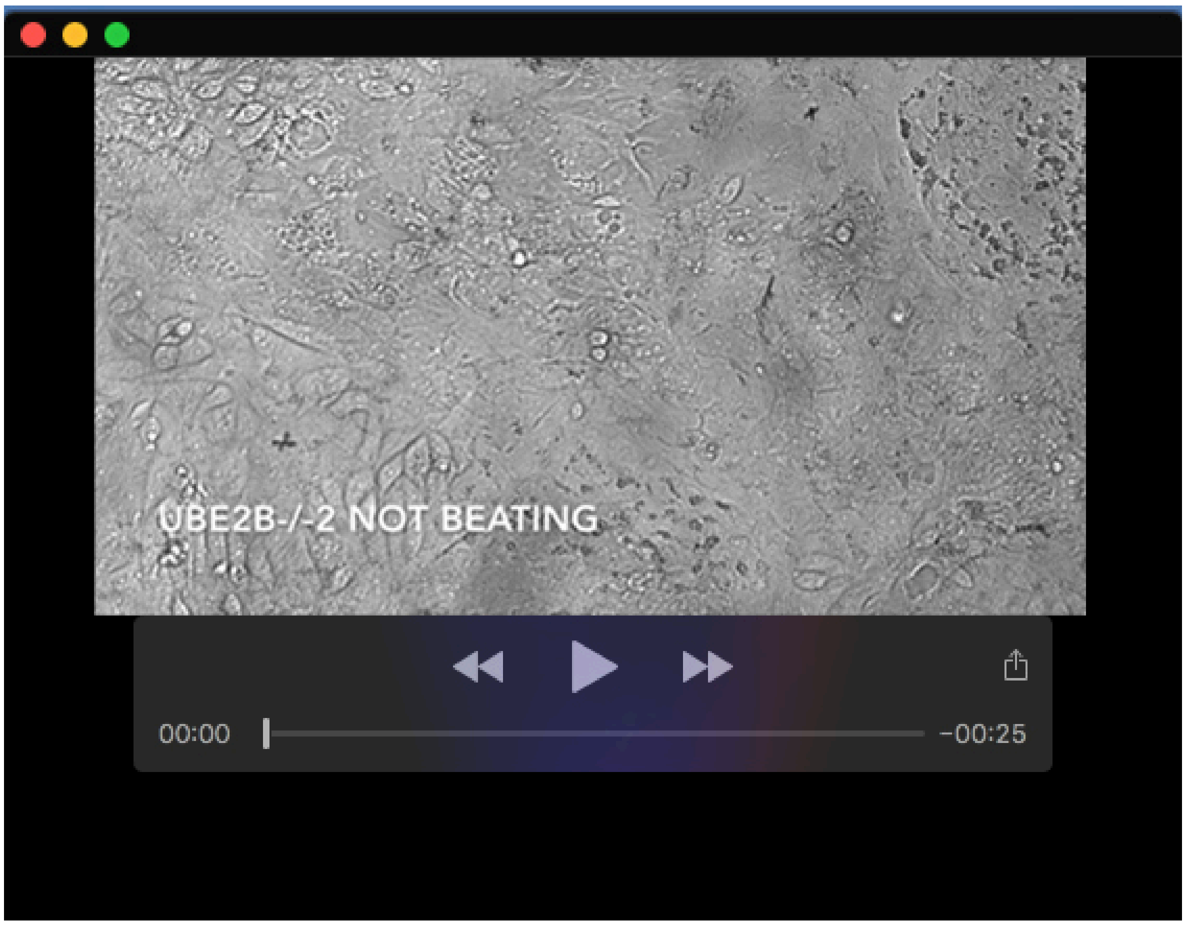

Movie 8. Movie shows *UBE2B*$^{-/-}$*2* cardiomyocytes not beating under bright-field microscopy.

Development • Supplementary information

References

Chung, Y.J., Luo, A., Park, K.C., Loonat, A.A., Lakhal-Littleton, S., Robbins, P.A., Swietach, P., 2019. Iron-deficiency anemia reduces cardiac contraction by downregulating RyR2 channels and suppressing SERCA pump activity. JCI Insight 4.

Gomez-Ospina, N., Panagiotakos, G., Portmann, T., Pasca, S.P., Rabah, D., Budzillo, A., Kinet, J.P., Dolmetsch, R.E., 2013. A promoter in the coding region of the calcium channel gene CACNA1C generates the transcription factor CCAT. PLoS One 8, e60526.

Santulli, G., Pagano, G., Sardu, C., Xie, W., Reiken, S., D'Ascia, S.L., Cannone, M., Marziliano, N., Trimarco, B., Guise, T.A., Lacampagne, A., Marks, A.R., 2015. Calcium release channel RyR2 regulates insulin release and glucose homeostasis. J Clin Invest 125, 1968-1978.
